# State of the Evidence Traffic Lights 2019: Systematic Review of Interventions for Preventing and Treating Children with Cerebral Palsy

**DOI:** 10.1007/s11910-020-1022-z

**Published:** 2020-02-21

**Authors:** Iona Novak, Catherine Morgan, Michael Fahey, Megan Finch-Edmondson, Claire Galea, Ashleigh Hines, Katherine Langdon, Maria Mc Namara, Madison CB Paton, Himanshu Popat, Benjamin Shore, Amanda Khamis, Emma Stanton, Olivia P Finemore, Alice Tricks, Anna te Velde, Leigha Dark, Natalie Morton, Nadia Badawi

**Affiliations:** 1 Cerebral Palsy Alliance Research Institute, Discipline of Child & Adolescent Health, Faculty of Medicine & Health, The University of Sydney, PO Box 6427, Frenchs Forest, Sydney, NSW 2086 Australia; 2 Department of Paediatric Neurology, Monash Health, Clayton, Victoria Australia; 3 Department of Paediatrics, Monash University, Clayton, Victoria Australia; 4 Grace Centre for Newborn Care, Children’s Hospital at Westmead, Westmead, New South Wales Australia; 5 Department of Paediatric Rehabilitation, Kids Rehab WA, Perth Children’s Hospital, Perth, Australia; 6 Department of Orthopedic Surgery, Boston Children’s Hospital, Harvard Medical School, Boston, MA USA; 7 Allied and Public Helath, Faculty of Health Sciences, Western Sydney University, Sydney, New South Wales Australia; 8 Allied and Public Helath, Faculty of Health Sciences, Western Sydney University, Sydney, New South Wales Australia; 9 School of Allied Health, Australian Catholic University, North Sydney, New South Wales Australia

**Keywords:** Cerebral palsy, Systematic review, Traffic light system, Evidence based, GRADE

## Abstract

**Purpose of Review:**

Cerebral palsy is the most common physical disability of childhood, but the rate is falling, and severity is lessening. We conducted a systematic overview of best available evidence (2012–2019), appraising evidence using GRADE and the Evidence Alert Traffic Light System and then aggregated the new findings with our previous 2013 findings. This article summarizes the best available evidence interventions for preventing and managing cerebral palsy in 2019.

**Recent Findings:**

Effective prevention strategies include antenatal corticosteroids, magnesium sulfate, caffeine, and neonatal hypothermia. Effective allied health interventions include acceptance and commitment therapy, action observations, bimanual training, casting, constraint-induced movement therapy, environmental enrichment, fitness training, goal-directed training, hippotherapy, home programs, literacy interventions, mobility training, oral sensorimotor, oral sensorimotor plus electrical stimulation, pressure care, stepping stones triple P, strength training, task-specific training, treadmill training, partial body weight support treadmill training, and weight-bearing. Effective medical and surgical interventions include anti-convulsants, bisphosphonates, botulinum toxin, botulinum toxin plus occupational therapy, botulinum toxin plus casting, diazepam, dentistry, hip surveillance, intrathecal baclofen, scoliosis correction, selective dorsal rhizotomy, and umbilical cord blood cell therapy.

**Summary:**

We have provided guidance about what works and what does not to inform decision-making, and highlighted areas for more research.

**Electronic supplementary material:**

The online version of this article (10.1007/s11910-020-1022-z) contains supplementary material, which is available to authorized users.

## Introduction

Cerebral palsy is the most common physical disability of childhood. In the last decade, major discoveries have been made in early diagnosis, prevention, and treatment, altering incidence, prognosis, and treatment responsivity. In high-income countries such as Australia, motor severity has lessened and the incidence of cerebral palsy has fallen by a staggering 30% [1]. Non-ambulant forms of cerebral palsy, co-occurring epilepsy, and co-occurring intellectual disability are less frequent, meaning more children than ever before can walk [2]. Epidemiologists propose that the reduction in incidence and severity is likely due to a combination of comprehensive obstetric and neonatal intensive care interventions.

In recent years, the cerebral palsy treatment evidence base has continued to expand rapidly, providing clinicians and families with the possibility of newer, safer, and more effective interventions. Since we last provided a comprehensive summary of the cerebral palsy intervention evidence in 2013, another 200+ systematic reviews have been published [3•]. This increasing volume of research evidence makes keeping up-to-date challenging for busy clinicians and overwhelming for families. Furthermore, the introduction of new interventions extends what clinicians need to know to allow sound clinical reasoning and decision-making [4].

This paper aimed to systematically describe the best available evidence for cerebral palsy interventions in 2019. We searched for the best available evidence published after 2012 and aggregated the new findings with our previous 2013 summary of evidence, using the updated GRADE system and the Evidence Alert Traffic Light System [5, 6]. The purpose of the paper was to describe what treatments have demonstrated evidence and highlight areas for more research. We rated the whole cerebral palsy intervention evidence base within the one paper to provide families, clinicians, managers, and policy makers with a helicopter view of best available intervention evidence to (a) inform decision-making by succinctly describing effective, emergent, and ineffective care; (b) aid comparative clinical decision-making about alike interventions and indications; and (c) provide a comprehensive resource to aid the creation of knowledge translation tools to promote evidence implementation.

## Methods

### Study Design

We conducted a systematic overview of best available evidence using the systematic review of systematic reviews methodology in order to provide an overview of the current state of the evidence [7].

### Search Strategy

Our review was carried out using a protocol based upon recommendations from the Cochrane Collaboration and reported according to the PRISMA statement [8, 9••]. Relevant articles were identified by searching: CINAHL (2012 to 2019); Cochrane Database of Systematic Reviews [www.cochrane.org]; EMBASE (2012 to 2019); ERIC (2012 to 2019); PubMED (2012 to 2019), PsycINFO (2012 to 2019), MEDLINE (2012 to 2019), OTSeeker [www.otseeker.com]; Physiotherapy Evidence Database (PEDro) [www.pedro.fhs.usyd.edu.au]; Psychological database for Brain Impairment Treatment Efficacy (PsycBITE) [www.psycbite.com]; PsycINFO (1935 to 2012); PubMED; and Speech Pathology Database for Best Interventions and Treatment Efficacy (speechBITE) [www.speechbite.com]. We sought to update and amalgamate the findings of our 2013 original paper [3•]. Searches were supplemented by hand searching. The search was performed in March–July 2019. Search terms for investigation replicated the same search strategy as our original paper and were supplemented by contributing authors’ knowledge of the field, e.g., names of new interventions published since 2012 to add to the search. We also searched for the intervention evidence about preventative treatments in the obstetric or neonatal period, given the considerable reduction in the incidence of cerebral palsy since our last publication.

Electronic databases were searched with OVID host software using PICOs search terms. The full search strategy is available from the authors on request.

### Inclusion Criteria

Published studies about interventions for children with cerebral palsy or at risk of cerebral palsy fulfilling the following criteria were included:

#### Type of Study

First, systematic reviews were preferentially sought [10]. Where multiple systematic reviews existed and newer evidence superseded the findings of earlier evidence, GRADEs were assigned based on the most recent high-quality evidence. We also searched for randomized controlled trials published after the latest systematic review, to account for new trials that might increase our confidence in the estimate of the treatment effect. For interventions where no systematic reviews existed, randomized controlled trials were preferentially sought, and where no randomized controlled trials existed, lower levels of evidence were included and appraised. New data (2012–2019) was then aggregated together with our data published in 2013 in order to review the entire evidence base. Second, retrieved bodies of evidence were appraised using the GRADE and Evidence Alert Traffic Light System using two independent raters, with unanimous agreement. GRADE is the evidence rating system endorsed by the World Health Organization [5, 6]. GRADE rates both (1) the *quality* of the evidence on a 4-point continuum of High–Moderate–Low–Very Low. Randomized trials start at a score of 4/4 (High) and can be downgraded based on methodological flaws; observational studies start at a score of 2/4 (Low) but can be upgraded based on methodological strengths or downgraded if methodological flaws exist; and (2) the *strength of the recommendation* for use, which weighs up the balance between the benefits and harms, the resource usage’ cost effectiveness, health equity, acceptability to consumers, and implementation feasibility [5]. When available, published outcomes of benefits were used to inform the strength of the recommendation. If no published literature was available, expert opinion was used. Recommendations were developed by the panel using the GRADE updated Evidence to Decision framework [5]. The Evidence Alert Traffic Light System was also applied to assist clinicians in obtaining clear, clinically useful answers within minutes [6]. The Evidence Alert uses a three-level traffic light color coding that recommends a course of action for implementation of the evidence within clinical practice. Green means go, because high-quality evidence from RCTs and systematic reviews indicates intervention effectiveness. Red means stop, because high-quality evidence from RCTs and systematic reviews indicates ineffectiveness or harm. Yellow means measure clinical outcomes, because either (i) promising evidence suggests possible effectiveness, but more research would increase our confidence in the estimate of the effect; or (ii) no research exists and therefore effects are unknown; or (iii) conflicting findings exists and therefore it is unclear how a patient might respond.

#### Types of Intervention

Studies that involved the provision of intervention either by a medical practitioner, an allied health professional, or an alternative and complementary medicine practitioner.

#### Types of Participants

Studies that explicitly involved human subjects. In the cerebral palsy preventative treatments evidence base, the participants were pregnant mothers or neonates. In the intervention evidence base, the participants were children living with cerebral palsy, in which > 25% of the participants had a diagnosis of cerebral palsy. We used a low cut off because many allied health interventions are provided using the exact same approach across multiple diagnostic groups (e.g., dysphagia management for stroke, brain injury, and cerebral palsy). We did not want to overlook important evidence that had been shown feasible and efficacious in the cerebral palsy population that was published within mixed population studies.

Studies were excluded from the review if (a) they were diagnostic, prognostic, or instrumentation studies; (b) they had lower levels of evidence, unless no systematic review or clinical trial had been published; (c) participants were adults; (d) they reviewed generic preventative interventions, e.g., good parenting; (e) they reviewed an entire discipline (e.g., physiotherapy, occupational therapy, speech pathology) and did not specify or sub-analyze individual named interventions but rather aggregated them together; (f) a second publication of the same study published the same results or participants; and/or (g) studies were unpublished or non-peer reviewed.

### Data Abstraction

A data abstraction sheet based on the Cochrane’s recommendations was used [8]. Abstracts identified from searches were screened by two independent raters to determine eligibility for further review. Abstracts were retained for full review if they met the inclusion criteria or if more information was required from the full-text to confirm the study met all eligibility criteria. Two independent reviewers then reviewed full-text versions of all retained articles and all additional articles identified by hand searching. Full-text articles were retained if they met inclusion criteria. Agreement on inclusion and exclusion assignment of the full-text articles was unanimous. Data extracted from included studies comprised citations, domains of impact of the intervention, level of the International Classification of Disability and Function (ICF) the intervention was aimed at, participants, study design, and dose. All the data required to answer the study questions were published within the papers, so no contact with authors was necessary.

### Ethics and Registration

The study did not involve contact with humans, so the need for ethical approval was waived by the Cerebral Palsy Alliance’s Human Research Ethics Committee. This systematic review was not registered.

## Results

**Fig. 1:**
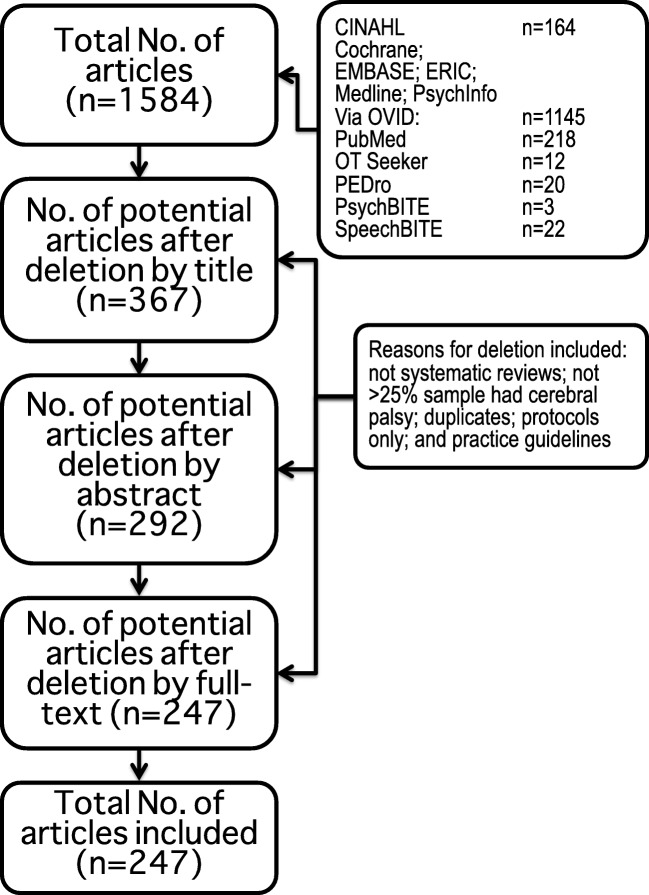
Flow diagram of included articles

One thousand five hundred eighty-four citations were identified using the search strategy, of which 247 articles met the inclusion criteria for review [9••, 10, 11••, 12–248]. The study flow is summarized in the PRISMA diagram (Fig. 1) [249].

We identified 182 interventions using our search strategy, an increase of 118 interventions from our 2013 review. Of these interventions, 41/182 (23%) were strategies aiming to prevent cerebral palsy and 141/182 (77%) were interventions aiming to manage cerebral palsy. The prevention strategies were categorized into antenatal prevention strategies (11/41, 27%) and neonatal prevention strategies (30/41, 73%). The interventions were categorized into allied heath interventions (83/141, 59%), pharmacological interventions (25/141, 18%), surgical interventions (19/141, 13%), regenerative medicine interventions (4/141, 3%), and complementary and alternative medicine (10/141, 7%). From these 182 interventions, we identified 393 intervention outcome indicators that had been studied in children with cerebral palsy. In five indications, two separate gradings were assigned, because the quality of the evidence was different in two sub-populations (e.g., ambulant versus non-ambulant) for the same intervention aim. This took the GRADE count by indication to a total of 398 indications.

Some of the included systematic reviews had already conducted quality ratings on the body of evidence using the GRADE system. As per the GRADE process, we confirmed whether or not we agreed with these findings and also carried out assignment of GRADE coding for all other included papers. Across the 398 intervention outcomes, the GRADE ratings were as follows: 14% of outcomes assessed (54/398) were graded “do it” (i.e., Green light, go interventions); 66% (264/398) were graded “probably do it” (i.e., Yellow light, weak positive); 17% (68/398) were graded “probably don’t do it” (i.e., Yellow light, weak negative); and 3% (*n* = 12/398) were graded “don’t do it” (i.e., Red light, stop interventions).

Each intervention was coded using the ICF by the intervention’s desired outcome. Out of the 383 intervention outcomes for children with CP identified in this study, *n* = 241/383 (62%) were aimed at the body structures and function level; *n* = 49/383 (13%) were aimed at the activity level; *n* = 12/383 (3%) were aimed at the participation level; *n* = 11/383 (3%) were aimed at the environmental factors level; *n* = 1/383 (< 1%) were aimed at the personal factors level; *n* = 58/383 (15%) were aimed at a combined body structures and activities level; and *n* = 11/383 (3%) were aimed at a combined activities and participation level.

### Participants

This study included participants with cerebral palsy, a complex and heterogeneous condition. We included studies involving any motor sub-type [spastic, dyskinetic, or ataxic], any topography [hemiplegic/unilateral, diplegic/bilateral, or quadriplegic/bilateral], and any functional ability level [Gross Motor Function Classification System (GMFCS) levels I–V and Manual Ability Classification System (MACS) levels I–V [250, 251]]. In the detailed supplementary extraction table (Online Resource, Table 1), we noted which interventions had been tested in the various sub-groups and severities.

The main results are detailed in the online table, which outlines the citation, the name of the intervention, the intervention indicator, the types of participants the intervention had been tested on, the dose/intensity used within the research studies, the GRADE ratings, the panels reflections on the evidence to decision recommendation process, and the clinical nuance of findings and considerations for interpretation. We strongly urge readers to read the detailed online resource to gain the necessary specifics for understanding the evidence base.

**Fig. 2:**
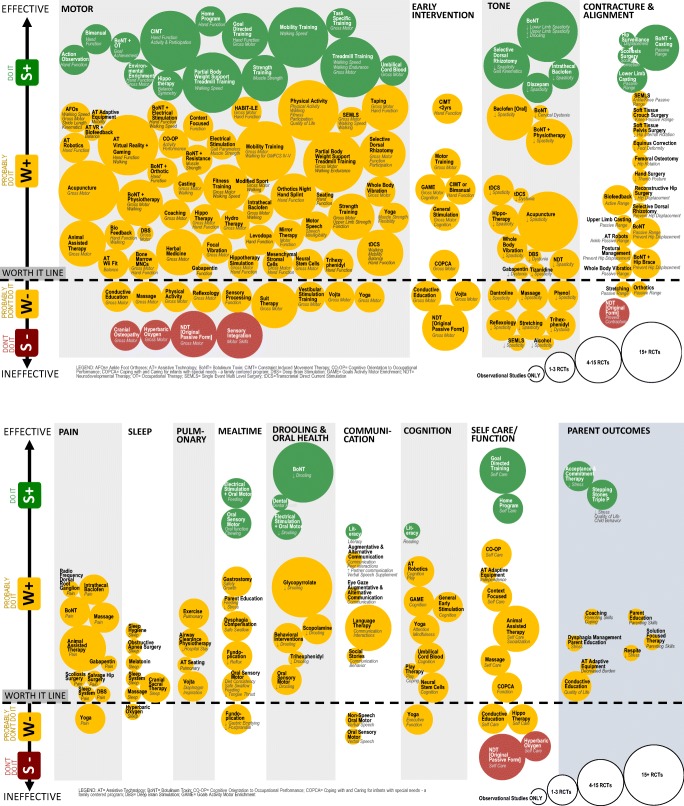

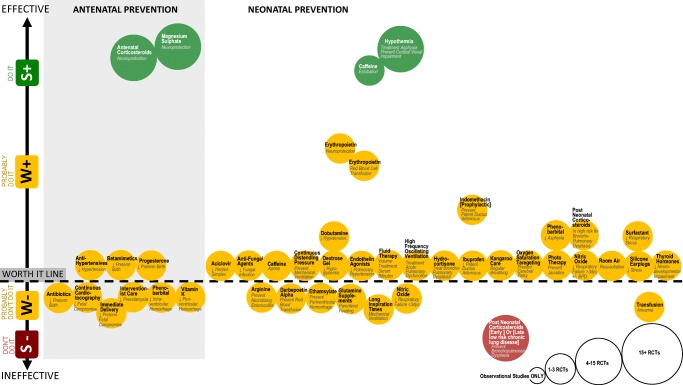
Evidence Alert System. AFOs ankle-foot orthoses, AT assistive technology, BoNT botulinum toxin, CIMT constraint-induced movement therapy, CO-OP cognitive orientation to occupational performance, COPCA coping with and caring for infants with special needs—a family centered program, DBS deep brain stimulation, GAME goals activity motor enrichment, NDT neurodevelopmental therapy, OT occupational therapy, SEMLS single-event multi-level surgery, tDCS transcranial direct current stimulation

To provide a summary of the online table and to assist with comparative clinical decision-making amongst intervention options for the same desired outcome, we mapped the interventions that seek to provide analogous outcomes, using bubble charts. In the bubble charts, the name of the circle is the intervention, and the italics under the title is the outcome measured and obtained. The size of the circle correlates to the volume of published evidence. The circle size was calculated by the amount and quality of evidence published. Bubble size 1, observational studies (OBS) only; size 2, 1–3 RCTs; size 3, 4–15 RCTs; and size 4, 15+ RCTs. The location of the circle on the *y*-axis of the graph corresponds to the GRADE system rating and estimate of effect (i.e., no effect was placed close to the worth it line, whereas a large treatment effect was placed further away from the worth it line). The color of the circle correlates to the Evidence Alert System (Fig. 2)

.

## Discussion

High levels of evidence exist in the literature summarizing effective preventive strategies and intervention options for children with cerebral palsy. There was an exponential increase in the number of systematic reviews and clinical trials published about cerebral palsy interventions since our last review. We observed a substantial increase in the number of systematic reviews published about acupuncture, pharmacological agents for managing tone, orthopedic surgery, dysphagia management, physical activity, participation, and clinical trials in regenerative medicine.

### Prevention of Cerebral Palsy

Undoubtedly, the most notable breakthroughs in the field of cerebral palsy research in the last decade have been made in the area of prevention [9••, 10, 11••, 12–18]. The rate of cerebral palsy has fallen by 30% in some high-income countries, bringing the prevalence down to 1.4 per 1000 [1, 2]. Babies born preterm constitute 43% of all cerebral palsy [2]. Antenatal magnesium sulfate before delivery of an infant less than 30 weeks’ gestation prevents 30% of cerebral palsy (green light) [9]. Antenatal corticosteroids decrease intracranial hemorrhage and thereby also act as an effective neuroprotectant (green light) and have become the standard of care [9]. More research would increase our confidence in the estimate of the effect, but further trials are not feasible as it would be unethical to withhold antenatal corticosteroids in premature birth. Once an infant is born preterm and is mechanically ventilated, prophylactic caffeine (methylxanthines) prior to extubation effectively prevents cerebral palsy (green light) [11••]. For babies born at term with neonatal encephalopathy or asphyxia, therapeutic hypothermia commenced within 6-h of delivery is neuroprotective and prevents 15% of cerebral palsy associated with intrapartum hypoxia (green light) [11••]. There is now a pressing ethical imperative to translate prevention breakthroughs and a range of public health initiatives from high-income countries to low-income and middle-income countries, where the disease burden is high [252]. For example, in Bangladesh, the rate of cerebral palsy is more than double that of Australia (3.4 versus 1.4 per 1000). Twice as many Bangladeshi children have severe motor impairments (GMFCS IV–V = 43.6%, compared with 26% in Australia), and 78.2% do not receive any rehabilitation [252]. Delayed umbilical cord clamping is also under investigation. As yet there is no specific data pertaining to whether delayed clamping prevents cerebral palsy, but we anticipate this will change in the near future and clinicians should stay abreast of this evidence base.

In recent years, our understanding of the genetic basis for cerebral palsy has advanced substantially [253]. A genetic contribution is likely in one-third of all children with cerebral palsy, especially in those without traditional risk factors such as prematurity and hypoxia [253]. As our understanding of neurobiology and genomics expands, the revolutionized field will result in the development of new prevention and treatment targets [253]. Experts also predict that future neuroprotective interventions will take advantage of trimester-specific brain development knowledge and that development of novel treatments will be informed by advances in biomarkers of brain injury, genetics, and neuroimaging [254].

The field has also started to critically examine whether repair of a brain injury might be possible using regenerative medicine treatments, paving the way towards finding a cure. Our review found that erythropoietin has promising effects as a neuro-regenerative treatment in the preterm population (yellow light, weak positive) and erythropoietin trials are underway in a population with hypoxic ischemic encephalopathy [11••]. In addition, there is now moderate-quality evidence that umbilical cord blood as a cell therapy, coupled with rehabilitation, is slightly more effective than rehabilitation alone for improving motor skills in children with cerebral palsy (green light) [221, 222]. The lack of legislation allowing access to autologous (patient’s own) and/or matched allogeneic (donor) cord blood makes the feasibility of this treatment challenging.

### Management of Cerebral Palsy

An intervention may target multiple desirable treatment outcomes, e.g., reduction of spasticity and improvement in function, and thus outcomes and levels of evidence could vary between outcomes. For most instances, the treatment outcome matched with the appropriate mechanism of action, e.g., pharmacological agent to reduce spasticity effectively reduced spasticity. There was often less convincing evidence (both in quality and volume) to support upstream effects for other treatment outcomes for other levels of the ICF, e.g., improvement in functional mobility. These are not surprising results; however, they provide an important reminder to clinicians to select interventions that address a child’s specific goal based on the intervention’s mechanism of action. Also, to be cognizant that applying more than one intervention simultaneously might be beneficial to achieve a goal where multiple goal-limiting factors are present. If the goal is to improve functional mobility: a pharmacological agent to reduce background spasticity (green light) [185] might make it easier to learn to move. Similarly, increasing lower limb muscle strength via strength training (green light) may improve related strength and endurance [151, 152], but principally targeted functional mobility training intervention will be required to establish an improvement in functional mobility (green light) [123, 127]. In all likelihood, the outcomes will be better if a combination of interventions are used. Some families believe that certain therapeutic approaches work for their child, but this was not possible to address within this review; however, we do not dismiss their views.

### Motor Interventions

All children with cerebral palsy have, by definition, a motor impairment and difficulties with tasks involving motor performance [255]. In high-income countries, severity is lessening, and the rate of co-occurring epilepsy and intellectual disability is falling [2]. Three in four will now walk [2]. This decline in severity is encouraging. Children with cerebral palsy may be more likely than ever to be treatment responsive to motor interventions, because smaller brain injuries result in improved baseline motor, sensory, and perceptual skills and learning capabilities. Thus, understanding current evidence for effective motor interventions is critically important. There is now a clear dichotomy in the evidence base for what works and what does not for improving function and performance of tasks. Substantive clinical trial data support the efficacy of training-based interventions, including action observation training [20, 21], bimanual training [54–56], constraint-induced movement therapy [46, 62–67], functional chewing training [137], goal-directed training [98], home programs using goal-directed training [112], mobility training [123, 127], treadmill training [65, 123, 127], partial body weight support treadmill training [123, 127, 169], and occupational therapy post botulinum toxin [190] (green lights). Moreover, environmental enrichment to promote task performance is effective (green light) [95] and adapting the environment and task to enable task performance via context-focused therapy (yellow light) [77] is a potent modulator of effective care. All these interventions have the following features in common: practice of real-life tasks and activities, using self-generated active movements, at a high intensity, where the practice directly targets the achievement of a goal set by the child (or a parent proxy if necessary). The mechanism of action is experience-dependent plasticity [256]. Motivation and attention are vital modulators of neuroplasticity, and successful task-specific practice is rewarding and enjoyable to children, producing spontaneously regular practice [256]. In stark contrast, bottom-up, generic, and/or passive motor interventions are less effective and sometimes clearly ineffective for improving function and movement for children with cerebral palsy. These include craniosacral therapy [239–241], hyperbaric oxygen [234, 235], neurodevelopmental therapy in the original passive format [108, 129–132], and sensory integration [3] (red lights). When viewed through the lens of neuroplasticity, these results are logical. A passive experience of a movement, provided via a hands-on therapeutic approach from a carer or therapist, does not involve any child-initiated problem solving or any child activation of their motor circuity.

There are also several adjunctive interventions that when combined with task-specific motor training may augment the positive effects of training. These include electrical stimulation [65, 92–94], hydrotherapy [108, 110, 111], taping [159–164], transcranial direct current stimulation [101, 166–168], and virtual reality serious gaming [33–47] (yellow lights, weak positive). These interventions warrant more research as children reported finding gaming interventions rewarding and normalizing, and preferred electrical stimulation to wearing ankle-foot orthoses from a comfort perspective [93]. Also, taping is better tolerated than traditional orthotics with children often reporting discomfort and dissatisfaction with these interventions or disliking the cosmetic effect [73, 140]. Other benefits from these adjunctive interventions include cardiorespiratory fitness and social integration, and the importance of which cannot be underestimated. Adjunctive suit therapy does not appear to have any additive benefit over and above motor training [156, 157]. Some children experience respiratory compromise, overheating, and peripheral cyanosis which resolve after removing the suit (yellow light, weak negative) [156, 157]. Suit therapy is therefore not recommended as a front-line treatment, or stand-alone treatment, nor should it be unsupervised [156, 157]. However, it is very important to recognize that for some families, the process and routine of donning a suit may mean they engage in more intensive therapies and active practice, which may produce positive results. We know that intensive task-specific motor practice is effective and works in a variety of treatment modalities [98]. The theory behind transcranial direct current stimulation having an augmentative effect to motor training, through provision of an additional targeted stimulation of the motor cortex, is logical, and more research is warranted [166–168].

The available studies about complementary and alternative medicine interventions for childhood cerebral palsy aimed to improve motor skills. Trials suggested efficacy with acupuncture [227, 228] and animal-assisted therapy [102] (yellow lights, weak positive). In contrast, conductive education [231, 232], massage [238], reflexology [243], Vojta [244–246], and Yoga [248] were probably ineffective for improving motor skills (yellow lights, weak negative), and cranial sacral osteopathy [239–241] and hyperbaric oxygen [234] showed no between-group differences for motor skills in moderate-quality trials and serious side effects occurred (red lights). Proponents of conductive education would claim that because the approach is holistic, that it is not reasonable to analyze indicators in isolation; nevertheless, these are the motor outcome results from published clinical trials. It is therefore important to note, conductive education may have benefits for social skills and quality of life outcomes [231]. The manual therapies, including massage (green light) [237] and cranial sacral osteopathy [241] and reflexology [243] (yellow lights, weak positive), appeared to help reduce constipation. Massage also appeared to help reduce pain [3•] (yellow light, weak positive), whereas Yoga did not [248] (yellow light, weak negative). However, Yoga did appear to improve attention, muscle flexibility, and balance (yellow light, weak positive) [248].

### Tone Management

Eighty-five percent of children with cerebral palsy have spasticity as their primary motor type and 7% have dyskinesia (including either dystonia or athetosis) as their primary motor type [2]. Many children have a mixed presentation involving both motor types [2]. Spasticity and dystonia cause involuntary movements and postures that affect motor control and can be painful. Our review identified that the following pharmacological agents and neurosurgical procedures effectively reduce spasticity: botulinum toxin [185], intrathecal baclofen [175, 176], diazepam [3•], and selective dorsal rhizotomy [209] (green lights), plus dantrolene [3•] and tizanidine [3•] are probably effective (yellow light). Supplementary local injections of alcohol probably reduce spasticity [3] (yellow light, weak positive), and local injections of phenol also probably reduce spasticity very short-term, but side effects are common (yellow light, weak negative) [195]. Less research involves dystonia management, given the lower prevalence and under-recognition of this motor disorder. Probably effective pharmacological agents for reducing dystonia include local injections of botulinum toxin [3•], oral gabapentin [193], intrathecal baclofen via a pump [177] (yellow light, weak positive), and oral trihexyphenidyl, which may reduce dystonic and athetoid involuntary movements and improve participation, but side effects may outweigh the benefits for some children (yellow light, weak negative) [177, 196]. There is as much art as there is science to prescribing pharmacological agents, especially for children with cerebral palsy that have multiple medical comorbidities. For example, in a child with combined dystonia and epilepsy, may benefit from using one medication that addresses both symptoms such as gabapentin, instead of two medications targeting the symptoms individually. Additionally, botulinum toxin [187], intrathecal baclofen [179, 180], and gabapentin [179] appear to reduce pain (yellow light, weak positive), which may further support the clinical decision to trial these agents, despite this not being the primary mechanism of these agents, as the multiple benefits may make them an acceptable intervention to children and parents. Deep brain stimulation appeared promising for children with dystonia that caused pain and severely limited daily participation and more research is warranted [177, 198].

Against the backdrop of spasticity management, there is a now an intense research focus on improved understanding of pathology, histochemistry, and muscle architecture in cerebral palsy [257]. Children with cerebral palsy appear to have elevated proinflammatory cytokines and genes involved in the extracellular matrix of their skeletal muscles, combined with increased intramuscular collagen and reduced ribosomal production [258]. Newer understandings of these pathophysiological muscle changes have led some clinicians to call for a reconsideration of botulinum toxin treatment, which induces therapeutic weakness and potential muscle fibrosis [259]. We do not yet know whether the observed atrophy and insertion of replacement fat and connective tissue observed in muscles of children with cerebral palsy is the result of a direct or accelerated adverse event from botulinum toxin or whether these changes are the natural history of cerebral palsy. We anticipate that more research into muscle pathology will both alter treatment recommendations over time and, more importantly, lead to the discovery of new interventions.

### Contracture Prevention and Management

Contracture is a common complication, particularly for children with spastic cerebral palsy. A longitudinal population-based study in Sweden has demonstrated that comprehensive multidisciplinary intervention at the right time can prevent contracture [260]. Contracture prevention and management should be thought of as a continuum, which will now be described. (a) In the early years, experts recommend high intensity self-generated active movement to prevent the onset of weakness, disuse and contracture [261]. While no clinical trial data is currently published supporting this idea, the hypothesis is currently being tested in clinical trials. (b) In Sweden, before contracture develops, the following interventions are used as part of comprehensive care: active movement, standing in standing shells (custom molded standing frames) for children GMFCS IV-V, and spasticity management using botulinum toxin where indicated. (c) Our review has shown that once a contracture has begun to develop, serial casting can be applied to effectively reduce or eliminate early/moderate contractures in the short term (green light). Notably, the skill of the practitioner in correctly aligning the joint and applying the cast is known to affect the result. For example, it is possible to perceive that increased range of motion has been achieved from casting, when in fact a further loss of biomechanical alignment of the midfoot (known as a midfoot break) has been induced, with no improvements in the hind foot. Casting effects can be enhanced by applying casts four weeks after botulinum toxin injections when the spasticity has been reduced (green light). Data indicates that children tolerate casting better when it is applied four weeks post toxin injection rather than immediately. The secondary weakness and altered proprioception induced from casts (with or without botulinum toxin) must be considered. Emergent evidence suggests that changing the casts at 3-day intervals rather than weekly intervals can shorten the total duration of the casting series and thus lower the amount of weakness induced. After casting, active strength training [60] (green light) and goal-directed training [98] (green light) are recommended to make functional use of the new range gained. (d) Once a contracture is severe (e.g., greater than 20°) and longstanding, casting will no longer be sufficient in isolation, and orthopedic surgery requires consideration. Some children and some muscles do not ever respond well to casting and proximal muscles cannot ever be cast; thus, surgical decision-making will be different in these scenarios. Moreover, casting requires regular appointments at specialist centers which may not be feasible for families in rural and remote locations. Orthopedic surgery may also be considered well before a contracture is severe, in order to maintain alignment, muscle length, and optimize biomechanics. The treating surgeon will consider the clinical examination, functional level, and child’s age, optimizing the timing of the surgery and minimizing the number of repeat procedures they will need over a childhood. Biomechanically, all joints in the lower limb work together in gait, meaning surgical lengthening of muscles at one joint impacts available range and control at other joints. Therefore, single-event multi-level surgery is a powerful intervention to simultaneously address the biomechanics of gait and minimize repeat surgeries (yellow light) [216, 217]. Our review has shown that traditional interventions for contracture management, including neurodevelopmental therapy [3•] (red light) and passive stretching in isolation [155] (yellow light), appear ineffective and the panel assigned negative recommendations since effective substitutes exist. In contrast, we found emergent low-quality evidence suggesting ankle robotics [32], biofeedback [30], botulinum toxin plus electrical stimulation [190], and whole-body vibration [97] may help manage contracture, by eliciting antagonist muscle activity that counterbalance involuntary agonist muscle contractions (yellow light), though more research is needed in this area.

### Hip Surveillance

One in three children in high-income countries experience progressive hip displacement as a complication of their cerebral palsy, except in the Nordic countries where rates are substantially lower [260, 262]. There is moderate-quality evidence and a strong recommendation to use comprehensive hip surveillance practices to facilitate early detection and management of hip displacement (green light). It may initially seem contradictory that hip surveillance is allocated a green light while the orthopedic and physiotherapy interventions designed to prevent hip displacement are coded yellow. This paper reports purely on the best available evidence, coded using the GRADE framework. We observe that interventions in isolation (including botulinum toxin, intrathecal baclofen, selective dorsal rhizotomy, obturator nerve blocks, positioning, and bracing) have small effect sizes for preventing hip migration [145, 147]. In contrast, important clues arise from longitudinal population-based studies in Sweden, which have shown that comprehensive multidisciplinary intervention (including botulinum toxin, weight-bearing, motor training, and orthopedic surgery) at the right time and the right dose can prevent hip dislocation [260]. We, therefore, conclude that management of the hip surveillance must be early, timely, and comprehensive, and clinical practice guidelines exist to inform and guide best management (https://www.ausacpdm.org.au/resources/australian-hip-surveillance-guidelines/).

### Physical Activity

Physical activity is essential for improving health but designing and implementing moderate to vigorous exercise programs for children with severe physical disabilities, who have limited movement and move slowly, is complex [263]. Recommendations to concurrently increase moderate to vigorous physical activity and replace sedentary behavior with light physical activity have been proposed to improve health [263]. New trials indicate that physical activity interventions (including exercise, activity training, strength training, and behavioral change strategies) probably improve fitness [144], physical activity [142–144], ambulation [144], mobility [144], participation, and quality of life [142] (yellow lights, weak positive). However, they do not appear to improve gross motor skills (yellow light, weak negative) [96, 144].

### Participation

We observed a shift in interventions that affected a child’s participation within their community. Most importantly, we noticed that interventions had been developed since 2013, which were specifically designed to target participation, and address barriers that prohibit participation and their effects were being studied in trials underway [264]. In other words, the targeted participation intervention was acting directly at the participation level of the international classification of function. There was a shift away from anticipating non-participation-based interventions might confer participation gains upstream.

### Dysphagia Management

Half of all children with cerebral palsy have dysphagia and the prevalence is even higher in the infant population [265]. One in 15 will require non-oral tube feeding [262]. Dysphagia management is extremely important because aspiration resulting in respiratory complication is a leading cause of death in individuals with cerebral palsy (45%) [266]. Experts have called for greater attention to respiratory health given the lack of preventative strategies and low levels of evidence for management strategies (airway clearance techniques, oral sensorimotor therapy, compensatory strategies such as positioning and thickening fluids, sialorrhea management, upper airway interventions, antibiotics, gastro-intestinal interventions, and spinal surgery) (yellow lights) [22]. We identified two newer dysphagia management approaches in the evidence base which positively address feeding skills and potentially lower the risk of aspiration: (a) Electrical stimulation plus oral sensorimotor therapy conferred better lip closure during swallowing, the ability to swallow food without excess loss, the ability to sip liquid, the ability to swallow liquid without excess loss, and the ability to swallow without cough than sham electrical stimulation plus oral sensorimotor therapy (green light) [138]. No adverse effects were reported in the studies included in this review; however, immediate and longitudinal safety concerns have not yet been well documented. As such, given that this intervention approach yields only modest benefits above and beyond oral sensorimotor therapy alone, a considered approach is warranted within a pediatric population. (b) A new motor learning–based oral sensorimotor intervention called functional chewing training (FuCT) appeared to improve chewing and reduce tongue thrust and sialorrhea better than traditional oral sensorimotor treatment alone [137] (yellow light), suggesting the direct training component was important. The FuCT findings are consistent with current thinking about motor learning. However, it must be noted that FuCT uses a combination of direct interventions, utilizing food or fluid; indirect interventions, utilizing non-nutritive tools to develop chewing skills; and sensory stimulation such as passive massage. Translation of this principle within the dysphagia management evidence base is becoming more prominent. Further research that compares direct, indirect, sensory, and compensatory interventions would be helpful in determining which approach results in greater skill development.

### Early Interventions

Rates of cerebral palsy following prematurity, encephalopathy, and neonatal surgery are well understood. It is now possible to accurately detect and diagnose cerebral palsy as early as three months of age (corrected), enabling much earlier intervention [267]. Previously only 61–64% of infants with cerebral palsy were referred for intervention before 12 months of age due to late diagnosis [267, 268]. This directly affected the volume and methodological quality of early intervention clinical trials conducted and published for infants with cerebral palsy. An important turning point in the field was the publication of a systematic review identifying that child-active motor learning early interventions appeared to confer improved movement and cognition (yellow light, weak positive), whereas passive approaches such as neurodevelopmental therapy produce no better movement skills than untreated controls (yellow light, weak negative) [79, 80]. Recently, there has been a burst of small pilot trials conducted, testing the feasibility, acceptability, and preliminary efficacy of a range of novel motor learning training-based interventions adapted to be infant-friendly. These novel interventions (baby-CIMT [85], baby-bimanual [86], GAME [83, 84], small steps [82]) have reported positive gains in movement skills (yellow light, weak positive) confirming the findings of Morgan et al.’s (2016) systematic review [79]. More extensive replication trials are underway in these early interventions using rigorous designs with adequate statistical power, meaning more will be known in the next few years about the efficacy of motor learning training-based early intervention for cerebral palsy.

In contrast, the feasibility and preliminary efficacy trials of a novel parent coaching-based approach (COPCA) disappointingly did not confer any gains over and above passive neurodevelopmental therapy within traditional physiotherapy (yellow light, weak positive) [87–90]. Likewise, conductive education [91] and Vojta therapy [79, 80] for infants with cerebral palsy also appear ineffective for improving movement skills (yellow light, weak negative). Neither of these approaches are based upon motor learning theory, and thus seem to further confirm the findings of the pivotal systematic review which identified motor learning to be key [79]. Trials into early interventions targeting other developmental domains which can be affected in cerebral palsy including cognition, feeding, and communication will emerge in the near future.

### Cognitive Interventions

Almost half of all children with cerebral palsy have co-occurring intellectual disability (46%) of varying severities, but notably, the prevalence of this comorbidity declining [1, 2, 262]. Co-occurring intellectual disability, coupled with severe physical disability, is known to elevate the risk for premature death during childhood [266]. With the shift in thinking about early motor interventions, the field has also started to explore whether the cognition of children with cerebral palsy can be modified and optimized. Early interactive reading and participation in early education settings, such as preschool, is known to improve intelligence in the typically developing and social risk populations, especially if these interventions include specific language development components [269]. In the cerebral palsy field, there is a shift towards actively recommending and testing these cognitive interventions with children with cerebral palsy. Our review found newer evidence of literacy interventions tailored for children with cerebral palsy using communication devices were effective (green light) [117, 118]. Infants that received GAME intervention (a combination of motor training, environmental enrichment, and coaching) had better cognition at 1 year of age than age-matched peers on a norm-referenced test (yellow light, weak positive) [83, 84]. More research on enriching the cognitive skills of infants with cerebral palsy is warranted.

Another innovation has been to test the feasibility, acceptability, and preliminary efficacy of a cognitive-based intervention known as cognitive orientation to occupational performance (CO-OP) [270]. CO-OP was originally designed for the developmental coordination disorder population where dyspraxia is the most important clinical sign [270], but now has promising evidence of efficacy for cerebral palsy, especially the dystonic type where treatment options are lacking [73–76]. In CO-OP, children set their own goals and are guided to discover and individualize strategies for successfully carrying out their goals, via a global problem-solving strategy “goal-plan-do-check” [270]. Once the child has self-identified a successful strategy, they practice the real-life task at high intensity, similar to other motor learning approaches [98]. Four studies have now been conducted in the cerebral palsy population suggesting CO-OP improves function at a low dose and low cost with large effect sizes (yellow light, weak positive) [73–76]. The conduct of a definitive trial is warranted.

### Parent Interventions

Parenting a child with cerebral palsy is known to be isolating and stressful. Supporting parents is essential both to optimize the child’s development and to protect a parent’s mental health. We observed that two interventions for parents of children with cerebral palsy, stepping stones triple P and acceptance and commitment therapy (ACT), now have empirical evidence of effectiveness (green lights) [19]. Stepping stones focuses on enhancing parenting skills and ACT focuses on increasing parental flexibility and enhancing a parent’s ability to use their parenting skills in a stressful context [19]. The early and intentional support of parents offers important possibilities for improving children’s outcomes.

## A Guide to Interpretation

This paper is not the personal opinions of the authors; instead, it is a summary of the best available published evidence in 2019. This paper does not, therefore, invalidate observations of a child’s response to interventions, even if they differ to average treatment responses measured in trials. Furthermore, it does not seek to criticize therapy choices of families or critique health care providers. Where evidence is not available, more well-designed trials are necessary. As cerebral palsy is a heterogeneous condition, the interpretation of the results from randomized controlled trials is complex.

Randomized controlled trials by their nature summarize the average response to an experimental treatment compared with that of a controlled comparison. In any given trial or real-world clinical scenario, an individual with cerebral palsy may respond better, or worse, than the average trial data. Heterogeneity is why many of the included trials have wide confidence intervals, indicating varied responses. We observed that often the trials with most robust treatment effects focused on homogeneous sub-groups of cerebral palsy (e.g., hemiplegia). In the future, alternative methodologies such as the *n* of 1 trial may accommodate the issue of heterogeneity.

To use the findings of this paper within clinical practice, we recommend the following: First, ask the child and family to define intervention goals. Second, match their goals to the outcome indicator headings and look up the corresponding intervention options with the associated levels of evidence. Third, select the intervention with the highest level of evidence and explain to families that on average, X intervention is most likely to help someone achieve their goals, and offer it. Monitor the individual effects of the intervention for the goal. Fourth, if the intervention is ineffective or unavailable, or the family declines (e.g., tried previously or side effects occurred), select the second most effective intervention and explain that on average, Y intervention is next most likely to help reach goals. Continue with this transparent conversation, compassionately acknowledging the disappointment if the child does not respond. Collectively problem solve a plan that matches the child’s capabilities and optimizes inclusion.

## Study Limitations

Our study has several limitations. First, a systematic review of systematic reviews is a study limitation in its own right because the methodology does not create any new knowledge that was not already published. In addition, the methodology of systematic reviews established by Cochrane favors the inclusion of randomized controlled trials, which may mean important observational studies are excluded or under-emphasized. Second, any summary lacks key details. Our helicopter view synthesis means that specific details about intervention fidelity, key ingredients, and best responders or non-responders are not reported or described in depth. We therefore advise clinicians and researchers to read additional literature to obtain this information, especially when adopting new interventions not previously used. Third, systematic reviews, despite being the highest level of evidence, are not without bias. Even though our review aimed to be unbiased, it included inherent biases. Publication bias may be at work within the included data we appraised, since trials with no between-group differences are less likely to be published in the first place, positively weighting the evidence base towards interventions that work. In addition, systematic reviews can be of varying methodological quality. Review authors may elect to include and review lower level evidence within their reviews to provide a more comprehensive picture of the evidence, but in doing so, provide a summary of highly biased data. We then have further summarized potentially biased data. The review authors may also have excluded relevant data, based on their inclusion criteria and the question they were seeking to answer. Fourth, in some of the included systematic reviews, we identified statistical errors, which we reinterpreted or reanalyzed where possible. For example, an accidental reversal of forest plots meaning the analysis was the opposite of the way the authors reported it. Another example was a misinterpretation of meta-analyses, where the confidence intervals around the standardized mean difference crossed the line of no effect, but the authors had made their interpretations based on the standardized mean difference alone and erroneously interpreted the intervention as effective. Fifth, despite our thorough search strategy, there is no guarantee that we retrieved and included all relevant systematic reviews, or important data published after the included reviews that might have changed our confidence in the estimate of the effects. Sixth, as we excluded articles not published in English and adhered to strict inclusion criteria regarding % of participants identified as having cerebral palsy, we may have overlooked important data and/or excluded recent reviews exploring relevant, non-CP-specific interventions (for example *Augmentative and Alternative Communication*) due to participant numbers not reaching the required threshold. Some of the studies included in the reviews have reported on cerebral palsy but that may not be the primary outcomes of those studies.

## Conclusion

Our paper systematically describes the best available evidence for cerebral palsy interventions in 2019, and highlights areas for more research. We found compelling evidence from systematic reviews to suggest the following: Green light prevention strategies: antenatal corticosteroids, magnesium sulfate, caffeine, and hypothermia. Green light allied health interventions: acceptance and commitment therapy, action observations, casting, constraint-induced movement therapy, environmental enrichment, fitness training, goal-directed training, hippotherapy, home programs, literacy interventions, mobility training, oral sensorimotor, oral sensorimotor plus electrical stimulation, pressure care, stepping stones triple P, strength training, task-specific training, treadmill training, partial body weight support treadmill training, and weight-bearing. Green light medical, surgical, pharmacological, and regenerative therapy interventions: anti-convulsants, intrathecal baclofen, bisphosphonates, botulinum toxin, botulinum toxin plus occupational therapy, botulinum toxin plus casting, diazepam, dental care, selective dorsal rhizotomy, scoliosis correction, hip surveillance, and umbilical cord blood cell therapy. In the last six years, many additional interventions have been researched, and the following interventions have been upgraded from emergent (yellow) to effective (green): Botulinum toxin plus adjunctive casting for increasing range of motion; goal-directed training for improving gross motor skills; hippotherapy for increasing symmetry; stepping stones triple P for improving child behavior; and strength training for improving muscle strength. There is a lack of robust clinical efficacy evidence for a large proportion of the interventions in use within standard care for people with cerebral palsy, and more research would increase our confidence in the estimate of effect. Thus, we have highlighted the need for more research using rigorous methodologies to advance the evidence base about interventions for cerebral palsy, to better inform decision-making by families and clinicians.

## Electronic Supplementary Material

ESM 1 (PDF 1569 kb)
