# Supplementary material for: State of the Evidence Traffic Lights 2019: Systematic Review of Interventions for Preventing and Treating Children with Cerebral Palsy
Source: Curr Neurol Neurosci Rep. 2020 Feb 21;20(2):3. doi: 10.1007/s11910-020-1022-z (PMC7035308; doi:10.1007/s11910-020-1022-z)
Supplement: Supplementary file 1 — (PDF 1569 kb) [file 11910_2020_1022_MOESM1_ESM.pdf]

Online Resources Table 1: Main Results

Intervention	Outcome [ICF Level]	Citations	Population	Dose	Panel Comments	GRADE		Traffic Light
						Quality	Recommendation	
**ANTENATAL INTERVENTIONS FOR THE PREVENTION OF CEREBRAL PALSY**								
**1 Antenatal Cortico-steroids**	1 Prevention of CP via lung development & neuro-protection [BS]	Shepherd 2017[9]	Pregnant mothers with risks for preterm labour	–	5 RCTs. Antenatal corticosteroids conferred a reduction in the rate of CP compared to placebo (RR 0.60, 95%CI 0.34-1.03). (-1 Risk of Bias; -1 Imprecision). Evidence to Decision Considerations: Methodological issues with studies including power. Intervention has become standard care and trials can no longer be conducted ethically. Status update since 2013: Newly listed intervention.	Low	Strong +	Green
	2 Prevention of CP via repeat doses for lung development & neuro-protection [BS]	Shepherd 2017[9]	Pregnant mothers with risks for preterm labour	–	4 RCTs. No between group differences for repeat doses of antenatal corticosteroids v single course of antenatal corticosteroids for prevention of CP (RR 1.03, 95%CI 0.71-1.50) (-1 Imprecision). Evidence to Decision Considerations: Methodological issues with studies including power. Intervention has become standard care and trials can no longer be conducted ethically. Status update since 2013: Newly listed intervention.	Moderate	Weak +	Yellow
**2 Antibiotics**	3 Prevention of CP via prevention of preterm birth [BS]	Shepherd 2017[9]	Pregnant mothers with intact membranes	–	1 RCT. Prophylactic antibiotics conferred higher rates of CP than no antibiotics (RR 1.82 95%CI 0.99-3.34) (-1 Imprecision). Weak negative (rather than strong negative) assigned as confidence intervals crossed the line of no effect. Status update since 2013: Newly listed intervention.	Moderate	Weak -	Yellow
**3 Anti-hypertensives**	4 Prevention of CP via treatment of hypertension [BS]	Shepherd 2017[9]	Pregnant mothers with hypertension	–	1 RCT. No between group differences for anti-hypertensives v placebo for prevention of CP (RR 0.33, 95%CI 0.01-8.01) (-1 Risk of Bias; -2 Imprecision). It is difficult to make conclusions with certainty; more research is needed. Status update since 2013: Newly listed intervention.	Very low	Weak +	Yellow
**4 Betamimetics**	5 Prevention of CP via prevention of preterm birth [BS]	Shepherd 2017[9]	Pregnant mothers with risks for preterm labour	–	1 RCT. No between group differences for betamimetics v placebo for prevention of CP (RR 0.19, 95%CI 0.02-1.63) (-1 Risk of Bias; -1 Imprecision). Status update since 2013: Newly listed intervention.	Low	Weak +	Yellow
		Neilson 2014[10]	–	–	Superseded by Shepherd 2017.			
**5 Continuous Cardiotocography (CTG)**	6 Prevention of CP via prevention of fetal compromise [BS]	Shepherd 2017[9]	Pregnant mothers during labour	–	2 RCTs. No between group differences for continuous CTG v intermittent auscultation for prevention of CP (RR 1.75, 95%CI 0.84-3.63) (-1 Risk of Bias; -1 Imprecision). Status update since 2013: Newly listed intervention.	Low	Weak -	Yellow
**6 Immediate**	7 Prevention	Shepherd	Pregnant	–	1 RCT. Immediate delivery conferred higher rates of CP than deferred delivery (RR 5.88	Moderate	Weak -	Yellow

	Delivery with Fetal Compromise		of CP via management of fetal compromise [BS]	2017[9]	mothers with preterm suspected fetal compromise		95%CI 1.33-26.02) (-1 Risk of Bias). Status update since 2013: Newly listed intervention.			
7	Interventionist Care	8	Prevention of CP via treatment of pre-eclampsia [BS]	Shepherd 2017[9]	Pregnant mothers with pre-eclampsia	–	1 RCT. No between group differences for interventionist care v expectant delayed delivery for prevention of CP (RR 6.01, 95%CI 0.75-48.14) (-2 Imprecision). Status update since 2013: Newly listed intervention.	Low	Weak -	Yellow
8	Magnesium Sulphate	9	Prevention of CP via neuroprotection [BS]	Shepherd 2017[9]	Pregnant mothers with risks for preterm labour	–	5 RCTs. Magnesium sulphate conferred a reduction in the rate of CP compared to placebo (RR 0.68, 95%CI 0.54-0.87) (No downgrades applied). Status update since 2013: Newly listed intervention.	High	Strong +	Green
		10	Prevention of CP via treatment of pre-eclampsia [BS]	Shepherd 2017[9]	Pregnant mothers with pre-eclampsia	–	1 RCT. No between group differences for magnesium sulphate v placebo for prevention of CP (RR 0.34, 95%CI 0.09-1.26) (-1 Risk of Bias; -1 Imprecision). Evidence to Decision Considerations: Ineffective for this indication but the medication is given anyway because of the neuroprotective effects. Status update since 2013: Newly listed intervention.	Low	Weak -	Yellow
		11	Prevention of CP via prevention of preterm birth [BS]	Shepherd 2017[9]	Pregnant mothers with threatening preterm labour	–	1 RCT. No between group differences for magnesium sulphate v other tocolytic agents for prevention of CP (RR 0.13, 95%CI 0.01-2.51) (-1 Risk of Bias; -1 Imprecision). Evidence to Decision Considerations: Ineffective for this indication but the medication is given anyway because of the neuroprotective effects. Status update since 2013: Newly listed intervention.	Very low	Weak -	Yellow
9	Phenobarbital	12	Prevention of CP via prevention of peri-ventricular haemorrhage [BS]	Shepherd 2017[9]	Pregnant mothers with risks for preterm labour	–	2 RCTs. No between group differences for phenobarbital v placebo for prevention of CP (RR 0.71, 95%CI 0.40-1.28) (-1 Risk of Bias; -1 Imprecision). Status update since 2013: Newly listed intervention.	Low	Weak -	Yellow
10	Progesterone	13	Prevention of CP via prevention of preterm birth [BS]	Shepherd 2017[9]	Pregnant mothers with a history of spontaneous preterm birth	–	1 RCT. No between group differences for prenatal progesterone v placebo for prevention of CP (RR 0.14, 95%CI 0.01-3.48) (-2 Imprecision). Evidence to Decision Considerations: Ineffective for this indication but the medication is given anyway because it is effective for the prevention of prematurity which is a risk factor for CP. Status update since 2013: Newly listed intervention.	Low	Weak +	Yellow
11	Vitamin K	14	Prevention of CP via prevention of peri-ventricular haemorrhage [BS]	Shepherd 2017[9]	Pregnant mothers with risks for preterm labour	–	1 RCT. No between group differences for vitamin K v placebo for prevention of CP (RR 0.77, 95%CI 0.33-1.76) (-1 Risk of Bias; -1 Imprecision; -1 Indirectness). Status update since 2013: Newly listed intervention.	Very low	Weak -	Yellow
**NEONATAL INTERVENTIONS FOR THE PREVENTION OF CEREBRAL PALSY**										

**12 Aciclovir**	15 Prevention of CP via treatment of herpes simplex [BS]	Shepherd 2018[11]	Neonatal: Infants with herpes simplex	–	1 RCT. No between group differences for acyclovir v darabine for prevention of CP (-1 Risk of Bias; -2 Imprecision). Evidence to Decision Considerations: Ineffective for this indication but the medication is given anyway because it is effective for the treatment of herpes simplex. Status update since 2013: Newly listed intervention.	Very low	Weak +	Yellow
**13 Air (Room)**	16 Prevention of CP via resuscitation [BS]	Shepherd 2018[11]	Neonatal: Infants at birth requiring resuscitation	–	1 RCT. No between group differences for room air v 100% oxygen for prevention of CP (RR 1.34, 95%CI 0.55-3.24) (-2 Risk of Bias; -1 Imprecision). It is difficult to make conclusions with certainty; more research is needed. Status update since 2013: Newly listed intervention.	Very low	Weak +	Yellow
**14 Anti-Fungal Agents**	17 Prevention of CP via prevention of fungal infection [BS]	Shepherd 2018[11]	–	–	1 RCT. No between group differences for systemic anti-fungal agents v placebo for prevention of CP (RR 0.96, 95%CI 0.45-2.03) (-2 Imprecision). Evidence to Decision Considerations: Ineffective for this indication but the medication is given anyway because it is effective for the prevention of fungal infection. Status update since 2013: Newly listed intervention.	Very low	Weak +	Yellow
**15 Arginine Supplementation**	18 Prevention of CP via prevention of necrotising enterocolitis [BS]	Shepherd 2018[11]	Neonatal: Infants born preterm	–	1 RCT. No between group differences for arginine supplementation v placebo for prevention of CP (RR 0.88, 95%CI 0.21-3.80) (-2 Imprecision). Status update since 2013: Newly listed intervention.	Low	Weak -	Yellow
**16 Caffeine (Methyl-xanthines)**	19 Prevention of CP via mechanical ventilation [BS]	Shepherd 2018[11]	Neonatal: Infants born preterm during endotracheal extubation	–	1 RCT. Prophylactic caffeine conferred better prevention of CP than standard care. (RR 0.54, 95%CI 0.32-0.92) (-1 Risk of Bias). Status update since 2013: Newly listed intervention.	Moderate	Strong +	Green
	20 Prevention of CP via prophylactic prevention of apnoea [BS]	Shepherd 2018[11]	Neonatal: Infants born preterm	–	1 RCT. No between group differences for prophylactic caffeine v placebo for prevention of CP (RR 1.03, 95%CI 0.43-2.49) (-1 Risk of Bias; -1 Imprecision). Evidence to Decision Considerations: Ineffective for this indication but the medication is given anyway because of the neuroprotective effects. Status update since 2013: Newly listed intervention.	Low	Weak +	Yellow
	21 Prevention of CP via treatment of apnoea [BS]	Shepherd 2018[11]	Neonatal: Infants born preterm	–	1 RCT. No between group differences for prophylactic caffeine v placebo for prevention of CP (RR 0.60, 95%CI 0.29-1.25) (-1 Risk of Bias; -1 Imprecision). It is difficult to make conclusions with certainty; more research is needed. Evidence to Decision Considerations: Ineffective for this indication but the medication is given anyway because of the neuroprotective effects. Status update since 2013: Newly listed intervention.	Low	Weak +	Yellow
	22 Prevention of vision impairment in CP [BS]	Chorna 2017[12]	Neonatal: infants born preterm	–	1 RCT. Caffeine confers improved visual perceptual skills compared to standard care. (No quality deductions). Evidence to Decision Considerations: Benefits outweigh harms. Status update since 2013: Newly listed intervention.	High	Strong +	Green
**17 Continuous Distending Pressure**	23 Prevention of CP via mechanical ventilation [BS]	Shepherd 2018[11]	Neonatal: Infants born preterm with respiratory distress syndrome	–	1 RCT. No between group differences for Continuous Distending Pressure v standard care for prevention of CP (RR 5.00, 95%CI 0.26-97.37) (-1 Risk of Bias; -1 Imprecision). It is difficult to make conclusions with certainty; more research is needed. Status update since 2013: Newly listed intervention.	Low	Weak +	Yellow
**18 Darbepoetin**	24 Prevention	Shepherd	Neonatal: Infants	–	1 RCT. No between group differences for darbepoetin alfa v placebo for prevention of CP	Low	Weak -	Yellow

**Alfa**	of CP via prevention of red blood cell transfusion [BS]	2018[11]	born preterm or low birthweight		(RR 0.08, 95%CI 0.00-1.40) (-1 Risk of Bias; -2 Imprecision). It is difficult to make conclusions with certainty; more research is needed. Evidence to Decision Considerations: Ineffective for this indication but the medication is under investigation for neuroprotective effects. Status update since 2013: Newly listed intervention.			
**19 Dextrose Gel**	25 Prevention of CP via treatment of hypoglycaemia [BS]	Shepherd 2018[11]	Neonatal: Infants at birth with hypoglycaemia	–	1 RCT. No between group differences for dextrose gel v placebo for prevention of CP (RR 5.16, 95%CI 0.25-106.12) (-1 Risk of Bias; -2 Imprecision). Status update since 2013: Newly listed intervention.	Very low	Weak +	Yellow
**20 Dobutamine**	26 Prevention of CP via treating hypotension [BS]	Shepherd 2018[11]	Neonatal: Infants born preterm with low superior vena cava flow	–	1 RCT. No between group differences for dobutamine v dopamine for treating hypotension (RR 0.16, 95%CI 0.01-2.64) (-1 Risk of Bias; -1 Imprecision). It is difficult to make conclusions with certainty; more research is needed. Status update since 2013: Newly listed intervention.	Very low	Weak +	Yellow
**21 Endothelin Receptor Agonists**	27 Prevention of CP via treatment of pulmonary hypertension [BS]	Shepherd 2018[11]	Neonatal: Infants with pulmonary hypertension born at term and late preterm	–	1 RCT. No between group differences for endothelin receptor agonists v placebo for treating persistent pulmonary hypertension (RR 0.09, 95%CI 0.00-1.61) (-1 Risk of Bias; -1 Imprecision). It is difficult to make conclusions with certainty; more research is needed. Status update since 2013: Newly listed intervention.	Low	Weak +	Yellow
**22 Erythropoietin**	28 Prevention of CP via neuro-protection [BS]	Fischer 2017[14]	Neonatal: Infants born preterm	–	2 RCTs. rhEPO showed a trend toward lowering the combined outcome of any neurodevelopmental impairment including CP [OR 0.55; 95% CI, 0.28–1.08; P = .08]. More research is needed with larger numbers given these are low incidence outcomes the studies are underpowered for this outcome. (-1 Risk of Bias). Status update since 2013: Newly listed intervention.	Moderate	Weak +	Yellow
	29 Prevention of CP via prevention of red blood cell transfusion [BS]	Shepherd 2018[11]	Neonatal: Infants born preterm or low birthweight	–	2 RCTs. No between group differences for erythropoietin v placebo for prevention of CP (RR 0.66, 95%CI 0.31-1.37) (-1 Risk of Bias; -2 Imprecision; -1 Inconsistency). It is difficult to make conclusions with certainty; more research is needed. Evidence to Decision Considerations: Ineffective for this indication but the medication is under investigation for neuroprotective effects. Status update since 2013: Newly listed intervention.	Very low	Weak -	Yellow
	30 Prevention of central vision impairment in CP [BS]	Chorna 2017[12]	Neonatal: Infants born preterm	–	1 RCT. 2 out of the 191 preterm infants treated with rhEPO had severe visual impairment compared to 0 out of 174 placebo control group, notably the rhEPO was high and repeated dose. Evidence to Decision Considerations: Emergent evidence suggests rhEPO should not be given to prevent visual impairments, but evidence rests on one study and the recommendations are therefore weak negative.	High	Weak -	Yellow
**23 Ethamsylate**	31 Prevention of CP via prevention of periventricular/ intraventricular haemorrhag	Shepherd 2018[11]	Neonatal: Infants born preterm or low birthweight	–	3 RCTs. No between group differences for ethamsylate v standard care for prevention of CP (RR 1.13, 95%CI 0.64-2.00) (-1 Imprecision). Status update since 2013: Newly listed intervention.	Moderate	Weak -	Yellow

	e [BS]							
**24 Fluid Therapy**	32 Prevention of CP via volume treatment [BS]	Shepherd 2018[11]	Neonatal: Infants born very preterm	–	1 RCT. No between group differences for volume therapy v no treatment for prevention of CP (RR 0.76, 95%CI 0.48-1.20) (-1 Imprecision). Status update since 2013: Newly listed intervention.	Moderate	Weak +	Yellow
	33 Prevention of CP via gelatin treatment [BS]	Shepherd 2018[11]	Neonatal: Infants born preterm or low birthweight	–	1 RCT. No between group differences for gelatin v fresh frozen plasma for prevention of CP (RR 0.94 95%CI 0.52-1.69) (-1 Imprecision). Status update since 2013: Newly listed intervention. Comparing 2 similar interventions therefore difficult to GRADE. FFP more commonly used.	Moderate	Weak -	Yellow
	34 Prevention of CP via treatment of serum bilirubin levels [BS]	Lai 2017[15]	Neonatal: Infants with unconjugated hyper-bilirubinaemia requiring photo therapy	–	7 RCTs. No between group differences for IV fluid supplementation lowering bilirubin encephalopathy, kernicterus, or CP in healthy, term newborn infants with unconjugated hyperbilirubinaemia requiring phototherapy. Evidence to Decision Considerations: no reported harms. (-1 Risk of Bias; -1 Indirectness). Status update since 2013: Newly listed intervention.	Low	Weak +	Yellow
**25 Glutamine Supple-mentation**	35 Prevention of CP via parenteral feeding [BS]	Shepherd 2018[11]	Neonatal: Infants born preterm	–	1 RCT. No between group differences for glutamine supplementation v placebo for prevention of CP (RR 1.07 95%CI 0.59-1.92) (-1 Risk of Bias; -1 Indirectness; -1 Imprecision). Status update since 2013: Newly listed intervention.	Very low	Weak -	Yellow
**26 High Frequency Oscillatory Ventilation**	36 Prevention of CP via mechanical ventilation [BS]	Shepherd 2018[11]	Neonatal: Infants born preterm with acute pulmonary dysfunction	–	3 RCTs. High Frequency Oscillatory Ventilation conferred better prevention of CP amongst survivors than conventional ventilation (-1 Risk of Bias; -1 Imprecision). Status: Not reported in 2013 review.	Low	Weak +	Yellow
**27 Hydrocortisone**	37 Prevention of CP via treatment of broncho-pulmonary dysplasia [BS]	Baud 2017[13]	Neonatal: Infants born preterm with chronic lung disease	–	1 RCT. Early low-dose hydrocortisone was not associated with higher rates of disability at 2 yrs of age, more research is needed. (-2 Risk of Bias). Status update since 2013: Newly listed intervention.	Low	Weak +	Yellow
**28 Hypothermia**	38 Prevention of CP via treatment of asphyxia [BS]	Shepherd 2018[11]	Neonatal: Neonates with asphyxia or encephalopathy	–	7 RCTs. Hypothermia conferred better prevention of CP than control standard care (RR 0.66, 95%CI 0.54-0.82) (No downgrading). There was no difference between the modes of cooling (caps v whole body), both are equally effective. Status update since 2013: Newly listed intervention.	High	Strong +	Green
	39 Prevention of central vision impairment in CP [BS]	Chorna 2017[12]	Neonatal: infants moderate-severe neonatal encephalopathy	–	5 RCT's. No significant reduction in blindness after receiving hypothermia as compared to non-cooled control group (RR 0.62 95% CI 0.38 to 1.01). Evidence to Decision Considerations: No specific evidence for this indication however since hypothermia is standard care for neuroprotection the treatment will be used anyway, and the potential benefits outweigh harms.	High	Strong +	Green
**29 Ibuprofen**	40 Prevention of CP via treatment of patent	Shepherd 2018[11]	Neonatal: Infants born preterm or low birthweight	–	1 RCT. No between group differences for oral ibuprofen v intravenous ibuprofen for prevention of CP (RR 1.35 95%CI 0.24-7.48) (-1 Risk of Bias; -2 Imprecision). Evidence to Decision Considerations: Stops blood being shunted away from the brain. Status update since 2013: Newly listed intervention.	Very low	Weak +	Yellow

	ductus arteriosus [BS]							
30 **Indomethacin**	41 Prevention of CP via prophylactic prevention of patent ductus arteriosus [BS]	Shepherd 2018[11]	Neonatal: Infants born preterm	–	4 RCTs. No between group differences for prophylactic indomethacin v placebo for prevention of CP (RR 1.20, 95%CI 0.77-1.40) (-1 Imprecision). Evidence to Decision Considerations: Stops blood being shunted away from the brain. Status update since 2013: Newly listed intervention.	Moderate	Weak +	Yellow
31 **Kangaroo Care (Skin-to-Skin)**	42 Prevention of CP via skin-to-skin care to promote regular breathing [BS]	Shepherd 2018[11]	Neonatal: Infants born preterm or low birthweight	–	1 RCT. No between group differences for kangaroo care v conventional care for prevention of CP (RR 0.65, 95%CI 0.21-2.02) (-1 Risk of Bias; -1 Imprecision). Status update since 2013: Newly listed intervention.	Low	Weak +	Yellow
		Conde-Agudelo 2016[16]	–	–	Superseded by Shepherd 2018			
32 **Long Inspiratory Times**	43 Prevention of CP via mechanical ventilation [BS]	Shepherd 2018[11]	Neonatal Neonates having mechanical ventilation	–	1 RCT. No between group differences for long v short inspiratory times for prevention of CP (RR 2.90 95%CI 0.97-8.65) (-1 Risk of Bias; -2 Imprecision). Status update since 2013: Newly listed intervention.	Very low	Weak -	Yellow
33 **Nitric Oxide**	44 Prevention of CP via treatment of respiratory failure [BS]	Shepherd 2018[11]	Neonatal: Infants born preterm <3 days	–	2 RCTs. No between group differences for inhaled nitric oxide v placebo for prevention of CP (RR 1.85, 95%CI 0.93-3.71) (-2 Imprecision). Status update since 2013: Newly listed intervention.	Low	Weak -	Yellow
	45 Prevention of CP via treatment of respiratory failure [BS]	Shepherd 2018[11]	Neonatal: Infants born preterm >3 days with broncho-pulmonary dysplasia risks	–	2 RCTs. No between group differences for inhaled nitric oxide v placebo for prevention of CP (RR 1.10, 95%CI 0.54-2.23) (-1 Risk of Bias; -1 Imprecision). Status update since 2013: Newly listed intervention.	Low	Weak +	Yellow
	46 Prevention of CP via treatment of respiratory failure [BS]	Shepherd 2018[11]	Neonatal: Infants born preterm and intubated	–	2 RCTs. No between group differences for inhaled nitric oxide v placebo for prevention of CP (RR 0.94, 95%CI 0.51-1.70) (-1 Risk of Bias; -1 Imprecision). Status update since 2013: Newly listed intervention.	Low	Weak +	Yellow
	47 Prevention of CP via treatment of respiratory failure [BS]	Shepherd 2018[11]	Neonatal: Infants born at or near full term	–	2 RCTs. No between group differences for inhaled nitric oxide v placebo for prevention of CP (RR 1.02, 95%CI 0.49-2.14) (-1 Risk of Bias; -1 Imprecision). Status update since 2013: Newly listed intervention.	Low	Weak +	Yellow
	48 Prevention of CP via treatment of	Shepherd 2018[11]	Neonatal: Infants born at or near full term with	–	1 RCT. No between group differences for inhaled nitric oxide v placebo for prevention of CP (RR 8.33, 95%CI 0.45-154.78) (-1 Risk of Bias; -1 Imprecision). Status update since 2013: Newly listed intervention.	Very low	Weak +	Yellow

	respiratory failure [BS]		diaphragmatic hernia					
**34 Oxygen Saturation**	49 Prevention of CP via varying oxygen saturation levels [BS]	Askie 2018[17]	Neonatal: Infants born preterm with breathing difficulties	Low: 85-89% for 2 wks High: 91-95% for 2 wks	5 RCTs in an individual patient meta-analysis. No between group differences for lower or higher oxygen saturation levels for prevention of CP (RD 1.7%, 95%CI −1.3 to 4.6%). Evidence to Decision Considerations: Trade-offs between benefits and harms may need to be considered. Infants receiving the lower oxygen range had a 2.8% increased risk of death, and a 2.2% increase in the rate of necrotising enterocolitis, but a 4.2% decrease in the rate of retinopathy of prematurity. Status update since 2013: Newly listed intervention.	High	Weak +	Yellow
		Askie 2017[18]	–	–	Superseded by Askie 2018.			
**35 Phenobarbital (Barbiturates)**	50 Prevention of CP via antiepileptic agents as a treatment of asphyxia [BS]	Shepherd 2018[11]	Neonatal: Neonates with asphyxia or encephalopathy	–	2 RCTs. No between group differences for phenobarbital v standard care for prevention of CP (RR 0.58, 95%CI 0.19-1.70) (-1 Risk of Bias; -1 Inconsistency; -1 Imprecision). NOTE: Harm may occur from not treating seizures, however preclinical data shows phenobarbital is neurotoxic and there a known risk that phenobarbital harms brain volume and cognitive development. Research is therefore underway to identify anti-convulsants that are not neurotoxic eg Kepra and Ganaxolone. Until new anti-convulsants are proven to work as an effective alternative, phenobarbital cannot be removed from standard care. Status update since 2013: Newly listed intervention.	Very low	Weak +	Yellow
**36 Phototherapy**	51 Prevention of CP via prevention of jaundice [BS]	Shepherd 2018[11]	Neonatal: Infants born preterm or low birthweight	–	2 RCTs. No between group differences for prophylactic phototherapy v standard care for prevention of CP (RR 0.96, 95%CI 0.50-1.85) (-1 Risk of Bias). Status update since 2013: Newly listed intervention.	Moderate	Weak +	Yellow
**37 Postnatal cortico-steroids**	52 Prevention of CP via early postnatal corticosteroids prevention of broncho-pulmonary dysplasia [BS]	Shepherd 2018[11]	Neonatal: Infants born preterm with chronic lung disease	–	12 RCTs. An increase in rates of CP occurred from early postnatal corticosteroids v standard care (RR 1.45, 95%CI 1.06-1.98). Status update since 2013: Newly listed intervention.	Moderate	Strong -	Red
	53 Prevention of CP via early inhaled postnatal corticosteroids prevention of broncho-pulmonary dysplasia [BS]	Shepherd 2018[11]	Neonatal: Infants born preterm with very low birth weight and having ventilation	–	1 RCT. No between group differences for early inhaled postnatal corticosteroids v placebo for prevention of CP. (RR 1.33, 95%CI 0.33- 5.42) (-1 Risk of Bias; -2 Imprecision). Status update since 2013: Newly listed intervention.	Very low	Weak -	Yellow
	54 Prevention of CP via moderately	Shepherd 2018[11]	Neonatal: Infants born preterm with chronic lung	–	4 RCTs. No between group differences for moderately early postnatal corticosteroids v placebo or no treatment for prevention of CP. Given there are risks for increased rates of CP from early postnatal corticosteroids this treatment is not recommended. (RR 1.03,	Very low	Weak -	Yellow

	early postnatal corticosteroids prevention of broncho-pulmonary dysplasia [BS]		disease		95%CI 0.47- 2.24) (-1 Risk of Bias; -2 Imprecision). NOTE an earlier analysis split the data into subgroups by relative risk and found that: The effect of postnatal corticosteroids on the combined outcome of death or cerebral palsy varied by the level of risk for chronic lung disease. More specifically: When the risk for chronic lung disease was below 35%, corticosteroid treatment significantly increased the chance of death or cerebral palsy, whereas when the risk for chronic lung disease exceeded 65%, corticosteroid treatment reduced the chance of cerebral palsy. We have therefore split these 2 sub-populations into separate bubbles on the bubble chart. Reference: Doyle LW, Halliday HL, Ehrenkranz RA, Davis PG, Sinclair JC. Impact of postnatal systemic corticosteroids on mortality and cerebral palsy in preterm infants: effect modification by risk for chronic lung disease. Pediatrics. 2005;115(3):655-661.			
	55 Prevention of CP via late postnatal corticosteroids prevention of broncho-pulmonary [BS]	Shepherd 2018[11]	Neonatal: Infants born preterm with chronic lung disease	–	14 RCTs. No between group differences for late postnatal corticosteroids v placebo or no treatment for prevention of CP. Given there are risks for increased rates of CP from early postnatal corticosteroids this treatment is not recommended. (RR 1.06, 95%CI 0.76- 1.50) (-1 Risk of Bias; -1 Imprecision). Status update since 2013: Newly listed intervention.	Low	Weak -	Yellow
**38 Silicone Earplugs**	56 Prevention of CP via prevention of stress [BS]	Shepherd 2018[11]	Neonatal: Infants born preterm or very low birth weight	–	1 RCT. No between group differences for silicone earplugs v no earplugs for prevention of CP (RR 3.00, 95%CI 0.14- 63.15) (-1 Risk of Bias; -2 Imprecision). Evidence to Decision Considerations: Lowers stress from environment. Status update since 2013: Newly listed intervention.	Very low	Weak +	Yellow
**39 Surfactant**	57 Prevention of CP via animal derived surfactant treatment of respiratory distress [BS]	Shepherd 2018[11]	Neonatal: Infants with respiratory distress syndrome	–	1 RCT. No between group differences for animal derived surfactant v no treatment for prevention of CP (RR 0.88, 95%CI 0.34- 2.27) (-1 Risk of Bias; -2 Imprecision). Status update since 2013: Newly listed intervention.	Very low	Weak +	Yellow
	58 Prevention of CP via synthetic surfactant treatment of respiratory distress [BS]	Shepherd 2018[11]	Neonatal: Infants born preterm	–	5 RCTs. No between group differences for synthetic surfactant v placebo for prevention of CP (RR 0.76, 95%CI 0.55- 1.05) (-1 Imprecision). Status update since 2013: Newly listed intervention.	Moderate	Weak +	Yellow
	59 Prevention of CP via prophylactic protein-free synthetic surfactant treatment of	Shepherd 2018[11]	Neonatal: Infants born preterm	–	4 RCTs. No between group differences for prophylactic protein-free synthetic surfactant v placebo for prevention of CP (RR 0.93, 95%CI 0.64-1.33) (-1 Risk of Bias; -1 Imprecision). Evidence to Decision Considerations: Not a clinically relevant. Status update since 2013: Newly listed intervention.	Low	Weak +	Yellow

	respiratory distress [BS]							
**40 Thyroid Hormones**	60 Prevention of CP via prevention of neuro-developmental impairment [BS]	Shepherd 2018[11]	Neonatal: Infants born preterm	–	1 RCT. No between group differences for thyroid hormones v placebo for prevention of CP (RR 0.72, 95%CI 0.28-1.84) (-2 Imprecision). Status update since 2013: Newly listed intervention.	Low	Weak +	Yellow
**41 Transfusion (restrictive v liberal)**	61 Prevention of CP via prevention of morbidity and mortality [BS]	Shepherd 2018[11]	Neonatal: Infants with very low birth weight	–	1 RCT. No between group differences for transfusion at a restrictive threshold versus a liberal threshold for preventing mobility and mortality (RR 1.29, 95%CI 0.55-3.03) (-1 Risk of Bias; -1 Imprecision). It is difficult to make conclusions with certainty; more research is needed. Status update since 2013: Newly listed intervention.	Low	Weak -	Yellow

ALLIED HEALTH INTERVENTIONS FOR MANAGING CEREBRAL PALSY

**42 Acceptance & Commitment Therapy [ACT]**	62 Improved parent stress [EF]	Whittingham 2016[19]	Parents of children with CP 2-12 yrs	6 group sessions	1 RCT. Stepping Stones Triple P + ACT conferred reduced parental stress compared to waitlist control. (-1 Risk of Bias). Status update since 2013: Newly listed intervention.	Moderate	Strong +	Green
**43 Action Observation Training**	63 Improved bimanual hand function [BS & A]	Buccino 2012[20]<br>Sgandurra 2013[21]	Unilateral spastic CP, ambulatory 5-15 yrs	15 hrs (1 hr/day for 15 days) x 3 wks	2 RCTs. Upper limb action observation training conferred better bimanual performance compared to watching videos, but with a small effect size (Cohen's d = 0.14). (-1 Inconsistency). Status update since 2013: Newly listed intervention.	Moderate	Strong +	Green
**44 Airway Clearance**	64 Number of hospital-isations, length of stay, antibiotics [BS]	Blackmore 2019[22]	CP <18yrs	20-30 mins 3x day OR 12 mins 2X day for 5mon	2 RCTS. It is unclear whether cough assist vs conventional physiotherapy +/- nebulized bronchodilators and high frequency and high frequency chest wall compression (HFCWC) vs conventional physiotherapy confers benefits to number of respiratory hospitalisations, length of stay, & or antibiotic useage. (-1 Risk of Bias, -1 Imprecision). Status update since 2013: Newly listed intervention.	Low	Weak +	Yellow
**45 Ankle Foot Orthoses [AFOs] Posterior Design**	65 Improved stride length [BS]	Betancourt 2019[23]<br>Lintanf 2018[24]<br>Aboutorabi 2017[25]	CP bilateral & unilateral spastic, ambulatory GMFCS I-III	4 wks duration. No daily dose reported.	2 meta-analyses, 1 RCT. AFOs increased stride length by 5 cm, compared to barefoot or shoes alone, with a large effect size (Cohen's d = 0.88). (-1 Inconsistency; +1 Large Effects). Evidence to Decision Considerations: Costs, harms (pain, skin irritation, impedance of transfers, impedance of ability to put on shoes, and cosmesis stigmatisation) may affect children's preferences and adherence, need to be weighed up against the 5 cm gains in stride length, which may not necessarily improve function nor be clinically meaningful. For this reason, we have downgraded the recommendation to weak positive. Status update since 2013: Quality upgraded from very low to high. Recommendations for use unchanged.	High	Weak +	Yellow
	66 Improved ankle kinematics (dorsi	Betancourt 2019[23]<br>Lintanf 2018[24]	CP Bilateral & unilateral spastic, ambulatory GMFCS I-III	Not reported	2 meta-analyses. AFOs increased ankle dorsiflexion by 8 degrees in stance, compared to barefoot or shoes alone, with a large effect size (Cohen's d = 1.3-1.7). Authors hypothesised AFOs decreased trips and falls but this was not tested. (-1 Inconsistency; +1 Large Effects). Evidence to Decision Considerations: Costs, harms (pain, skin	High	Weak +	Yellow

	flexion) [BS]				irritation, impedance of transfers, impedance of ability to put on shoes, and cosmesis stigmatisation) may affect children's preferences and adherence, need to be weighed up against the gains in ankle kinematics, which may not necessarily improve function. For this reason, we have downgraded the recommendation to weak positive. Status update since 2013: Newly listed indication.			
	67 Improved walking speed [A]	Lintanf 2018[24] Aboutorabi 2017[25]	CP Bilateral & unilateral spastic, ambulatory GMFCS I-III	Not reported	1 meta-analysis and 1 RCT. AFOs increased walking speed, compared to barefoot or shoes alone, with a small effect size (Cohen's d = 0.28). Authors did not discuss whether these gains were clinically meaningful. (-1 Inconsistency). Evidence to Decision Considerations: Costs, harms (pain, skin irritation, impedance of transfers, impedance of ability to put on shoes, and cosmesis stigmatisation) may affect children's preferences and adherence. Status update since 2013: Newly listed indication.	Moderate	Weak +	Yellow
	68 Improved gross motor function [A]	Lintanf 2018[24]	CP Bilateral & unilateral spastic, ambulatory GMFCS I-III	Not reported	1 meta-analysis. AFOs conferred small improvements in standing, walking, running and jumping (GMFM Sections D&E) compared to barefoot or shoes alone, with a small effect size (Cohen's d = 0.28-0.30). Evidence to Decision Considerations: Costs, harms (pain, skin irritation, impedance of transfers, impedance of ability to put on shoes, and cosmesis stigmatisation) may affect children's preferences and adherence. Decreased power and subsequent running speed may influence which functional activities children use AFOs for. Status update since 2013: Newly listed indication.	Moderate	Weak +	Yellow
**46 Assistive Technology – Adaptive Equipment**	69 Improved independence in self-care [A & P]	–	–	–	Status update since 2013: No new primary source data, no change in quality or recommendations for use gradings.	Low	Weak +	Yellow
	70 Improved computer access [A]	–	–	–	Status update since 2013: No new primary source data, no change in quality or recommendations for use gradings.	Very low	Weak +	Yellow
	71 Improved mobility via powered wheelchair [A]	–	–	–	Status update since 2013: No new primary source data, no change in quality or recommendations for use gradings.	Low	Weak +	Yellow
	72 Improved participation [P]	–	–	–	Status update since 2013: No new primary source data, no change in quality or recommendations for use gradings.	Very low	Weak +	Yellow
	73 Improved transfers [A]	–	–	–	Status update since 2013: No new primary source data, no change in quality or recommendations for use gradings.	Very low	Weak +	Yellow
	74 Reduced caregiver burden [EF]	–	–	–	Status update since 2013: No new primary source data, no change in quality or recommendations for use gradings.	Very low	Weak +	Yellow
**47 Assistive Technology – Adaptive Seating Systems [AdSS]**	75 Improved postural control [BS]	Angsupaisal 2015[26]	CP non ambulant GMFCS IV-V <19 yrs	Varied	17 observational studies. Adaptive seating systems conferred short term gains in activity and participation, including upper limb function. (-1 Risk of Bias; -1 Inconsistency). Evidence to Decision Considerations: Very important to be seated in the upright position for mobility and inclusion. High cost. Status update since 2013: No change in quality or recommendations for use gradings.	Very Low	Weak+	Yellow
	76 Improved pulmonary function [BS]	–	–	–	Status update since 2013: No new primary source data, no change in quality or recommendations for use gradings.	Very low	Weak +	Yellow
	77 Improved hand	–	–	–	Status update since 2013: No new primary source data, no change in quality or recommendations for use gradings.	Very low	Weak +	Yellow

	function [A]							
	78 Reduced pressure via tilt [BS]	–	–	–	Status update since 2013: No new primary source data, no change in quality or recommendations for use gradings.	Very low	Weak +	Yellow
**48 Assistive Technology – Sleep Positioning Systems [SPP]**	79 Prevent hip migration percentage [BS]	Humphreys 2019[27]	CP (GMFCS III-V) 5-16 yrs	1-4 nights in sleep system	2 observational studies. Sleep systems did not prevent hip migration. (-1 Risk of Bias). Considerations: Costs, variable user acceptance. Status update since 2013: Newly listed indication.	Very low	Weak +	Yellow
	80 Reduced pain [BS]	Humphreys 2019[27]	CP (GMFCS III-V) 5-16 yrs	1-4 nights in sleep system	2 observational studies. Sleep systems did not reduce pain. (-1 Risk of Bias). Evidence to Decision Considerations: Costs, variable user acceptance. Status update since 2013: Newly listed indication.	Very low	Weak +	Yellow
	81 Improved sleep quality [BS]	Humphreys 2019[27]	CP (GMFCS III-V) 5-16 yrs	1-4 nights in sleep system	2 observational studies. Sleep systems did not improve sleep quality. (-1 Risk of Bias; -1 Inconsistency). Evidence to Decision Considerations: Costs, variable user acceptance. Status update since 2013: No change in quality or recommendations for use gradings.	Very low	Weak +	Yellow
		Blake 2015[28]	–	–	Superseded by Humphreys 2019			
		Galland 2012[29]	–	–	Superseded by Humphreys 2019			
**49 Assistive Technology: Robots**	82 Improved arm function [BS & A]	Chen 2016[30]	CP mixed 4-15 yrs	1 hr 2-3x day 3-8 wks	1 RCT with cross over, 8 observational studies. Robotic therapy appeared to confer improved arm function (-1 Risk of Bias). Status update since 2013: Newly listed indication.	Low	Weak +	Yellow
	83 Improved cognition and play [BS]	Cruz 2017[31]	25% sample CP 5.92 yrs	18 min, 9 sessions over 9 wks	1 RCT (ASD population), 33 observational studies. Robotics conferred improved switch and device operation in some studies but not all (-1 Risk of Bias; -1 Imprecision). Evidence to Decision Considerations: Costs. Status update since 2013: Newly listed indication.	Very low	Weak +	Yellow
	84 Improved ankle range of motion [BS]	Zhang 2013[32]	CP mixed 5-15yrs	Varied	4 observational studies in CP. Robot-assisted ankle rehabilitation training appeared to confer improved range of motion (-1 Risk of Bias; -1 Imprecision). Evidence to Decision Considerations: Costs. Status update since 2013: Newly listed indication.	Very low	Weak +	Yellow
**50 Assistive Technology: Virtual Reality [VR] & Gaming**	85 Improved arm function & postural control [BS & A]	Chen 2018[33]	CP 4-12 yrs	20-90 mins/ day, 1-7x wk, over 4-20 wks	19 RCTs. VR conferred better arm function than NDT or usual care, with large effect sizes (Cohen's d = 0.86). (-1 Inconsistency). VR conferred better postural control than usual care, with large effect sizes (Cohen's d = 1.01). Findings suggest that games are to be used as a complement to conventional therapies and not as a substitute. VR may also induce neuroplasticity. Evidence to Decision Considerations: Costs, as engineer devised technology was more effective than off-the-shelf technology increasing the costs. Status update since 2013: Quality upgraded from very low to moderate. Recommendations for use unchanged.	Moderate	Weak +	Yellow
		Lopes 2018[34]	–	–	Superseded by Chen 2018			
		Rathinam 2018[35]	–	–	Superseded by Chen 2018			
		Holtz 2018[36]	–	–	Superseded by Chen 2018			
		Hickman 2017[37]	–	–	Superseded by Chen 2018			
		Page 2017[38]	–	–	Superseded by Chen 2018			
		Ravi 2017[39]	–	–	Superseded by Chen 2018			

		Chiu 2015[40]	–	–	Superseded by Chen 2018			
		Staiano 2014[41]	–	–	Superseded by Chen 2018			
		Weiss 2014[42]	–	–	Superseded by Chen 2018			
		Chen 2014[43]	–	–	Superseded by Chen 2018			
		Bonnechere 2014[44]	–	–	Superseded by Chen 2018			
		Tatla 2014[45]	–	–	Superseded by Chen 2018			
		Inguaggiato 2013[46]	–	–	Superseded by Chen 2018			
		Tatla 2013[47]	–	–	Superseded by Chen 2018			
	86 Improved ambulation [BS & A]	Chen 2018[33]	CP 4-12 yrs	20-90 mins/ day, 1-7x wk, over 4-20 wks	19 RCTs. VR conferred better ambulation than treadmill training or usual care, with moderate effect sizes (Cohen's d = 0.75). (-1 Inconsistency). Evidence to Decision Considerations: Costs - engineer devised technology was more effective than off-the-shelf technology increasing the costs. Status update since 2013: Newly listed indication.	Moderate	Weak +	Yellow
**51 Assistive Technology: VR + Biofeedback**	87 Improved balance [BS]	Yoo 2017[48]	CP 7-15 yrs	30 mins	1 observational study, comparing CP to typically developing. Virtual reality + biofeedback appeared to confer better balance than virtual reality alone. (-1 Risk of Bias). Status update since 2013: Newly listed intervention.	Very low	Weak +	Yellow
**52 Assistive Technology: Wii Fit**	88 Improved balance [BS]	Cooper 2017[49]	CP GMFCS I-III 5-18 yrs	6 wks	1 RCT, 5 observational studies. Wii Fit appeared to confer improved balance. (-1 Risk of Bias). Evidence to Decision Considerations: May be less effective in children with cognitive impairment. Status update since 2013: Newly listed intervention.	Low	Weak +	Yellow
	89 Improved energy expenditure [BS]	Deutsch 2015[50]	CP GMFCS I-V 9-21 yrs		4 observational studies. Wii Fit appeared to improve energy expenditure levels. (-1 Risk of Bias). Evidence to Decision Considerations: It is unclear if improved energy expenditure leads to any clinically meaningful changes. Status update since 2013: Newly listed intervention.	Very low	Weak +	Yellow
**53 Augmentative & Alternative Communication [AAC]**	90 Improved general communication [A]	–	–	–	Status update since 2013: No new primary source data, no change in quality or recommendations for use gradings.	Very low	Weak +	Yellow
	91 Improved communication of pre-schoolers [A]	–	–	–	Status update since 2013: No new primary source data, no change in quality or recommendations for use gradings.	Very low	Weak +	Yellow
	92 Improved communication partner skills [P]	–	–	–	Status update since 2013: No new primary source data, no change in quality or recommendations for use gradings.	Very low	Weak +	Yellow
	93 Enhanced verbal speech supplementation [A]	–	–	–	Status update since 2013: No new primary source data, no change in quality or recommendations for use gradings.	Very low	Weak +	Yellow
	94 Improved	Therrien	Mixed disability	Varied	19 observational studies. AAC (involving teaching interactions skills) conferred improved	Very low	Weak +	Yellow

	peer interaction [A & P]	2016[51]	including CP		peer interactions. (-1 Risk of Bias; - Imprecision). Evidence to Decision Considerations: Communication is a human right. Risks exists for a behaviour disorder from communication frustration if AAC is not provided. Status update since 2013: Newly listed indication.			
**54 Augmentative & Alternative Communi-cation [AAC] Eye Gaze**	95 Improved communication across different social contexts [A & P]	Karlsson 2018[52]	Physical disability including CP dystonia, diplegia & tetraplegia GMFCS IV-V 5-15 yrs	14 days for 9-10 mo incl. training + support	2 observational studies, only 1 study had participants with CP (CFCS IV-V). Eye gaze conferred improved communication. (-1 Risk of Bias; -1 Imprecision; -1 Indirectness). Evidence to Decision Considerations: Costs, need for training and support to promote success. Risks exists for a behaviour disorder from communication frustration if eye gaze technology is not provided. High acceptability, when supports in place. Status update since 2013: Newly listed intervention.	Very low	Weak +	Yellow
**55 Behaviour Therapy**	96 Improved child behaviour [A]	–	–	–	New data exists and this intervention has been recoded as Stepping Stones Triple P.	–	–	–
**56 Behavioural Intervention – Sialorrhea**	97 Reduced sialorrhea [BS]	McInerney 2019[53]	Neurodisability including CP 5-17 yrs	Varied between studies	1 RCT and 7 observational studies. (-1 risk of bias, -1 small sample size, - imprecision). Report of significantly reduced sialorrhea frequency however reduction not maintained	Very low	Weak +	Yellow
**57 Bimanual Training**	98 Improved hand function [BS & A]	Ferre 2017[54]	CP hemiplegia 2-10 yrs	90 hrs day in camp	1 new RCT. Home-based hand-arm bimanual intensive therapy (H-HABIT) conferred better improvements in hand function than control lower-limb functional intensive training (LIFT). (-1 Risk of Bias). Status update since 2013: No change in quality or recommendations for use gradings.	Moderate	Strong +	Green
		Brandao 2018[55]	CP hemiplegia 4-12 yrs	90 hrs continuous v 90 hrs distributed	1 RCT. 90 hrs of bimanual therapy in 1 intensive continuous block [6 hr 5x wk for 3 wks] was equally effective to 90 hrs of bimanual therapy in 2 blocks with a break [6 hr 5x wk for 1.5 wks (45 hrs) then again 6 mo later] for improving hand function. Repeating the 45 hrs did not lead to better outcomes, gains were equal at 45 hrs v 90 hrs. (-1 Risk of Bias). Evidence to Decision Considerations: Both doses work. Threshold dose has previously proposed to be 90 hrs, but perhaps is closer to 45 hrs.			
		Friel 2016[56]	CP hemiplegia	90 hrs	1 small RCT. No between group differences for structured bimanual therapy v unstructured bimanual therapy for improved hand function, both approaches work. (-1 Risk of Bias).			
	99 Improved neuroplasticity [BS]	Friel 2016[56]	CP hemiplegia	90 hrs	1 small RCT. Structured bimanual therapy conferred greater changes in neuroplasticity on the motor maps than unstructured bimanual therapy. Both groups improved in bimanual hand function. (-1 Risk of Bias). Evidence to Decision Considerations: Structured practice produces superior results, confirming what is known about goal-directed and task-specific training. Status update since 2013: Newly listed indication.	Moderate	Weak +	Yellow
**58 Biofeedback**	100 Improved active range of motion [BS]	Chen 2016[30]	CP <17 yrs	3x wk for 6 wks	1 new RCT. No between groups differences for biofeedback with a robot + passive stretching at home v biofeedback with a robot + passive stretching in a lab for ankle range of motion. Both groups improved equally, suggesting the environment for this intervention is not critical. (-2 Risk of Bias: no control group – we don't know what to attribute the increase to). Status update since 2013: No change in quality or recommendations for use gradings.	Low	Weak +	Yellow
	101 Improved walking [A]	Van Gelder 2017[57]	CP GMFCS I-III 6-16 yrs	N/A	1 observational study. Children with CP were able to respond to real-time visual feedback, resulting in significant and clinically relevant improvements in peak hip and knee extension during gait. Status update since 2013: No change in quality or recommendations for use gradings.	Low	Weak +	Yellow
	102 Improved hand	Rattanathar n 2017[58]	CP 5-14 yrs	60 mins day, 3x wk, 4 wks	1 new RCT. Biofeedback conferred faster performance of hand skills than control. (-2 Risk of Bias: baseline non-equivalence favouring biofeedback group).	Low	Weak +	Yellow

	function [A]	Abdel-Rahman 2014[59]	CP hemiplegia 5-7 yrs		1 new RCT. Biofeedback on a computer program conferred better grip strength than usual care. (-2 Risk of Bias). Status update since 2013: Upgraded quality from very low to low. No change in recommendations for use grading.			
**59 Casting**	103 Improved passive range of motion lower limbs [BS]	Tustin 2017[60]	CP GMFCS I-III 2-10 yrs	Variable Most common 3x 1 wk	4 previous systematic reviews and 5 new studies (3 RCTs, 2 controlled trials). (-1 Indirectness). Casting consistently conferred improved dorsiflexion range of motion. Evidence to Decision Considerations: Pain. Short term duration effects. Status update since 2013: No change in quality or recommendations for use gradings.	Moderate	Strong +	Green
	104 Improve passive range of motion upper limbs [BS]	–	CP	–	Status update since 2013: No additional systematic reviews on efficacy of improved range of motion in the upper limbs, therefore grading remains the same.	Low	Weak +	Yellow
	105 Improved function [A]	–	CP	–	Status update since 2013: No additional systematic reviews on efficacy of improved function, therefore grading remains the same.	Low	Weak +	Yellow
	106 Reduced muscle spasticity [BS]	Tustin 2017[60]	CP GMFCS I-III 2-10 yrs	Variable Most common 3x 1 wk	4 previous systematic reviews and 5 new studies (3 RCTs, 2 controlled trials). Inconsistent findings for casting conferring reduction in spasticity. Casting may enhance spasticity reduction. (-1 Inconsistency; -1 Indirectness). Evidence to Decision Considerations: Pain. Effective spasticity management substitutes exist, casting should not be a frontline treatment for spasticity management, but may be an added secondary benefit if casting is in use for another indication. Status update since 2013: Recommendations for use upgraded from weak negative to weak positive.	Low	Weak +	Yellow
	107 Improved gait parameters [BS]	Tustin 2017[60]	CP GMFCS I-III 2-10 yrs	Variable Most common 3x 1 wk	4 previous systematic reviews and 5 new studies (3 RCTs, 2 controlled trials). Inconsistent findings for casting conferring improvements to gait parameters. (-1 Inconsistency; -1 Indirectness). Evidence to Decision Considerations: Pain. Short term duration effects. Status update since 2013: Newly listed indication.	Low	Weak +	Yellow
	108 Improved gross motor function [BS & A]	Tustin 2017[60]	CP GMFCS I-III 2-10 yrs	Variable Most common 3x 1 wk	4 previous systematic reviews and 5 new studies (3 RCTs, 2 controlled trials). Inconsistent findings for casting conferring improvements to gross motor function. (-1 Inconsistency; -1 Indirectness). Evidence to Decision Considerations: Pain. Effective substitutes for improving gross motor exist. Status update since 2013: Newly listed indication.	Low	Weak +	Yellow
**60 Coaching Parents**	109 Improved parenting skills and coping [EF]	–	–	–	Status update since 2013: No new primary source data, no change in quality or recommendations for use gradings.	Very low	Weak +	Yellow
	110 Improved gross motor skills [BS & A]	Novak 2014[61]	CP GMFCS I-III 5-12 yrs	1 hr/day, 3 days/wk for 3 mo	1 RCT. Motor learning coaching conferred greater retention in gross motor skills than NDT (-1 Risk of Bias). Status update since 2013: Newly listed indication.	Moderate	Weak +	Yellow
**61 Cognitive Behaviour Therapy [CBT]**	111 Reduced depression, anxiety [BS]	–	–	–	Status update since 2013: No new primary source data, no change in quality or recommendations for use gradings.	Very low	Weak +	Yellow
**62 Constraint Induced Movement Therapy [CIMT]**	112 Improved unimanual capacity and bimanual performance	Hoare 2019[62]	CP hemiplegia 3 mo – 19 yrs	0.5-8 hrs, 2-7x wk, for 1-10 wks (137 hrs)	36 RCTs. CIMT conferred better bimanual performance and unimanual capacity than low-dose therapy, which was not well defined. CIMT may also induce neuroplasticity. (-1 Risk of Bias; +1 Consistent result across trials). Status update since 2013: No change in quality or recommendations for use gradings.	High	Strong +	Green
		Sakzewski	–	–	Superseded by Hoare 2019			

	[BS & A]	2014[63]						
		Chen 2014[64]	–	–	Superseded by Hoare 2019			
		Das 2019[65]	–	–	Superseded by Hoare 2019			
		Jamali 2018[66]	–	–	Superseded by Hoare 2019			
		Fonseca 2017[67]	–	–	Superseded by Hoare 2019			
		Inguaggiato 2013[46]	–	–	Superseded by Hoare 2019			
	113 Activity and Participation [A & P]	Chiu 2016[68]	CP hemiplegia 2-10 yrs	2-84 hrs for 2-10 wks	31 RCTs; 15 compared CIMT to no intervention, 7 studies comparing CIMT with an upper limb intervention of equal intensity. CIMT conferred better activity and participation gains than no therapy, with large effect sizes. CIMT was equally effective to dose matched OT for producing activity and participation gains. Authors proposed the mechanism for improvements relates to intensity of practice rather than the type of intervention, consistent with the conclusions of previous reviews. (-1 Indirectness: outcome measures were incorrectly classified on the ICF). Status update since 2013: No change in in quality or recommendations for use gradings.	Moderate	Strong +	Green
		Adair 2015[69]	–	–	Superseded by Chiu 2016			
	114 Improved tactile function [BS]	Auld 2014[74]	CP Hemiplegia	2-6 hrs x day	2 small non-randomised trials. One trial demonstrated improvements in stereognosis and the other trial showed no benefits (-1 Risk of Bias; -1 Inconsistency; -1 Indirectness; -1 Imprecision). Status update since 2013: Newly listed indication.	Very low	Weak +	Yellow
**63 CIMT v Bimanual Intensive Training**	115 Improved bimanual performance [BS & A]	Hoare 2019[62]	CP hemiplegia 3 mo – 19 yrs	0.5-8 hrs, 2-7x wk, for 1-10 wks (137 hrs)	CIMT was equally effective for improving bimanual performance and unimanual capacity as high-dose matched OT or HABIT (bimanual training). [MD −0.39, 95% CI −3.14 to 2.36]. (-1 Risk of Bias; + 1 Consistent result across trials). CIMT was equally effective for improving bimanual performance dose matched HABIT (bimanual training). [MD 0.80, 95%CI −0.78 to 2.38]. All systematic reviews that compare CIMT with Bimanual Intensive Training concluded that both approaches led to similar improvements in upper limb function. Evidence to Decision Considerations: Since CIMT is equally effective to bimanual, it recommended that both approaches are used, or the family selects the one that suits their preferences. Status update since 2013: No change in in quality or recommendations for use gradings.	Moderate	Strong +	Green
		Dong 2013[70]	–	–	7 RCTs. Superseded by Hoare 2019.			
		Sakzewski 2014[63]	–	–	Superseded by Hoare 2019			
		Klepper 2017[71]	–	–	Superseded by Hoare 2019			
		Tervahauta 2017[72]	–	–	Superseded by Hoare 2019			
**64 Cognitive Orientation to Occupational Performance [CO-OP]**	116 Improved function [A]	Jackman 2018[73]	CP mixed GMFCS I-IV 4-15 yrs	10 hrs + home program	1 RCT. CO-OP conferred better function than functional hand splints alone. (-1 Risk of Bias). Evidence to Decision Considerations: High acceptability to children and parents. Low dose therefore lower cost than other motor learning approaches. Status update since 2013: Newly listed intervention.	Low	Weak +	Yellow
		Cameron 2017[74]	CP mixed 7-12 yrs	10 hrs + home program	1 small RCT. No between group differences for CO-OP and usual care for improving function. (-2 Risk of Bias: under-powered & control group also received CO-OP). Status update since 2013: Newly listed intervention.			

		Gimeno 2019[75]	CP dystonic GMFCS I-IV 6-21 yrs	10 hrs + home program	1 observational study. CO-OP conferred improved function. (+1 Large Effects). Evidence to Decision Considerations: CO-OP appears effective for teaching functional skills to children with dystonic CP. This is a very important finding given the lack of alternatives for this population. Status update since 2013: Newly listed intervention.			
		Peny-Dahlstrand 2018[76]	CP mixed + spina bifida 16–28 yrs	10 hrs + home program	1 observational study. CO-OP conferred improved function. (-1 Risk of Bias; -1 Indirectness). Evidence to Decision Considerations: High acceptability to adolescents. Low dose therefore lower cost than other motor learning approaches. Status update since 2013: Newly listed intervention.			
**65 Context Focussed Therapy**	117 Improved self-care [A]	Kruijsen-Terpstra 2015[77]	CP GMFCS I-IV 11 mo – 4 yrs		2 RCTs, 1 new RCT. No between group differences for context focussed v child focussed v usual care. (-1 Risk of Bias). Evidence to Decision Considerations: Since context focussed is equally effective to child focussed, it recommended that both approaches are used simultaneously, or the family selects the one that suits their preferences. Status update since 2013: The field has come to understand more about how to specifically train improvements in self-care skills through task specific training, goal-directed training and cognitive orientation to occupational performance. With this added data, the effect size of context focussed therapy is now less clear given that is compared to child-focussed therapy &/or usual care, which is an umbrella term for many different therapies with varying effect sizes. We have therefore downgraded the quality from high to moderate and recommendations for use from strong positive to weak positive.	Moderate	Weak +	Yellow
**66 Counselling [Parents]**	118 Improved mental health [EF]	–	–	–	New data exists and this intervention has been recoded as Acceptance and Commitment Therapy	–	–	–
**67 Dysphagia Management – Compensatory Strategies**	119 Improved safety of swallow via thickened textures [BS]	–	–	–	Status update since 2013: No new primary source data, no change in quality or recommendations for use gradings.	Low	Weak +	Yellow
	120 Improved safety of swallow via positioning [BS]	–	–	–	Status update since 2013: No new primary source data, no change in quality or recommendations for use gradings.	Low	Weak +	Yellow
**68 Dysphagia Management – Parent Education**	121 Improved feeding [A & P]	Mlinda 2018[78]	CP mixed types moderate-severe 2 yrs	6-8 sessions 6-8 30 min OT + 1 home visit	1 RCT. Parent education + pictorial feeding position sheets + OT focused on positioning & supporting feeding conferred better feeding than general health education. (-1 Risk of Bias; 1 Indirectness; -1 Imprecision). Evidence to Decision Considerations: Low risk of harm. Status update since 2013: Newly listed indication.	Very low	Weak +	Yellow
	122 Improved mood [BS, A & P]	Mlinda 2018[78]	CP mixed types moderate-severe 2 yrs	6-8 sessions 6-8 30 min OT + 1 home visit	1 RCT. Parent education + pictorial feeding position sheets + OT focused on positioning & supporting feeding conferred better feeding than general health education. (-1 Risk of Bias; 1 Indirectness; -1 Imprecision). Evidence to Decision Considerations: Low risk of harm. Status update since 2013: Newly listed indication.	Very low	Weak +	Yellow
	123 Reduced caregiver stress [EF]	Mlinda 2018[78]	CP mixed types moderate-severe 2 yrs	6-8 sessions 6-8 30 min OT + 1 home visit	1 RCT. Parent education + pictorial feeding position sheets + OT focused on positioning & supporting feeding conferred better feeding than general health education. (-1 Risk of Bias; 1 Indirectness; -1 Imprecision). Evidence to Decision Considerations: Low risk of harm. Status update since 2013: Newly listed indication.	Very low	Weak +	Yellow
**69 Early Intervention – Motor Training**	124 Improved gross motor skills [A]	Morgan 2016a[79]	CP & High risk of CP 0-2 yrs	Varied	3 new systematic reviews. Active motor training conferred better motor skills than passive motor approaches. Clinical trials included children without CP affecting the interpretation. (-1 Indirectness). Status update since 2013: Quality rating has been upgraded, no change	Moderate	Weak +	Yellow

		Hadders-Algra 2017[80]	–	–	in recommendation for use grading.			
		Case-Smith 2013[81]	–	–				
		Das 2019[65]	–	–	Superseded by Morgan 2016			
		Holmstrom 2019[82]	CP & High risk of CP 0-2 yrs	Home visits + daily practice for 39 wks in 6 wk blocks	1 small RCT. Small steps used 6 wk blocks focussed on mobility, hand function & communication. No between group differences for "Small Steps" motor training compared to usual care. However, the trial was underpowered and Small Steps appeared to offer a protective effect for children with more severe motor impairments, who caught up to those with better ability at baseline. (-1 Risk of Bias; -1 Indirectness). Evidence to Decision Considerations: High acceptability of the intervention. Status update since 2013: Quality rating has been upgraded, no change in recommendation for use gradings.			
**70 Early Intervention – NDT**	125 Improved gross motor skills [A]	Morgan 2016a[79]	CP & High risk of CP 0-2 yrs	Varied	2 new systematic reviews. Children who received NDT in its original format that included passive movement had worse motor skills. Clinical trials included children without CP affecting the interpretation. (-1 Indirectness). Status update since 2013: Quality rating has been downgraded, no change in recommendation for use grading.	Moderate	Weak -	Yellow
		Hadders-Algra 2017[80]	–	–				
**71 Early Intervention – GAME**	126 Improved gross motor skills [A]	Morgan 2015[83] & 2016b[84]	CP & High risk of CP 0-2 yrs	Weekly home visits + daily practice	2 small RCTs. "GAME" intervention (motor training + motor enrichment + parent coaching) conferred better improvement in motor skills than usual care, with moderate effect sizes but wide confidence intervals. (-1 Risk of Bias; -1 Imprecision). Evidence to Decision Considerations: High acceptability of the intervention. Status update since 2013: Newly listed indication.	Low	Weak +	Yellow
	127 Improved cognitive skills [BS]	Morgan 2016b[84]	CP & High risk of CP 0-2 yrs	Weekly home visits + daily practice	1 small RCT. "GAME" intervention (motor training + motor enrichment + parent coaching) conferred better improvement in cognitive skills than usual care, but wide confidence intervals. (-1 Risk of Bias; -1 Imprecision). Evidence to Decision Considerations: High acceptability of the intervention. Given cognition is important for academic performance and independent living long-term, these data are important. Status update since 2013: Newly listed indication.	Low	Weak +	Yellow
**72 Early Intervention – CIMT**	128 Improved hand function [BS & A]	Eliasson 2018[85]	CP hemiplegia & High risk of CP 0-2 yrs	Varied. Usually daily practice	1 small RCT. Constraint Induced Movement Therapy conferred better hand function than massage. Evidence to Decision Considerations: The is high level evidence supporting the effectiveness of CIMT in children over 2 yrs of age, and this study confirms efficacy in the younger age range, and importantly did not identify any harms to the dominant hand when administered in the recommended dose. Status update since 2013: Newly listed indication in this age group.	Moderate	Weak +	Yellow
**73 Early Intervention – CIMT v Bimanual**	129 Improved hand function [BS & A]	Chamudot 2018[86]	CP hemiplegia & High risk of CP 3-6 mo	1 hr day for 8 wks	1 small RCT. No between group differences for Constraint Induced Movement Therapy or Bimanual Training, both interventions conferred improved hand skills with large effect sizes. (-1 Risk of Bias; -1 Indirectness). Evidence to Decision Considerations: High acceptability of the intervention. Status update since 2013: Newly listed indication in this age group.	Low	Weak +	Yellow
**74 Early Intervention – Coping with and Caring for Infants with Special Needs (COPCA)**	130 Improved gross motor skills [BS & A]	Hielkema 2019a[87]	CP & high risk of CP GMFCS I-V 0-9 mo 18 mo follow-up	30-60 mins 1x wk for 1 yr	1 small RCT. No between group differences for "COPCA" intervention and traditional infant physiotherapy (NDT) for improving motor skills. (-2 Risk of Bias: not all participants had CP at trial completion). Status update since 2013: Newly listed intervention.	Low	Weak +	Yellow
		Hielkema 2019b[88]			Duplicate participant data, therefore not reported twice.			
		Blauw-Hospers	CP & high risk of CP	COPCA: 2x wk	1 small RCT. No between group differences for "COPCA" intervention and traditional infant physiotherapy (NDT) for improving motor skills. (-2 Risk of Bias: not all participants			

		2011[89]	3-6 mo<br>18 mo follow-up		had CP at trial completion). Status update since 2013: Newly listed intervention.			
		Hielkema 2011[90]			Duplicate participant data, therefore not reported twice.			
	131 Improved function [A & P]	Hielkema 2019b[88]	CP & high risk of CP<br>GMFCS I-V<br>0-9 mo<br>18 mo follow-up	30-60 mins 1x wk for 1 yr	1 small RCT. No between group differences for "COPCA" intervention and traditional infant physiotherapy (NDT) for improving functional skills on the PEDI. (-2 Risk of Bias: not all participants had CP at trial completion). Status update since 2013: Newly listed intervention.	Low	Weak +	Yellow
	132 Improved quality of life [BS, A, P, EF, PF]				1 small RCT. "COPCA" intervention conferred better improvements in quality of life on the ITQOL. (-2 Risk of Bias: not all participants had CP at trial completion). Status update since 2013: Newly listed intervention.	Low	Weak +	Yellow
75 **Early Intervention – Conductive Education**	133 Improved gross motor skills [BS & A]	Ziviani 2010[91]	Varied diagnosis<br>0-9 yrs	Varied	Status update since 2013: No new primary source data on efficacy of improved motor skills from Conductive Education, therefore grading remains the same. Status update since 2013: Newly listed indication in this age group.	Low	Weak -	Yellow
76 **Early Intervention – General Stimulation**	134 Improved gross motor skills [A]	Morgan 2016a[79]	CP & High risk of CP<br>0-2 yrs	Varied	3 new systematic reviews. General stimulation programs conferred better motor skills than passive motor approaches. Only RCT with a positive effect is by Palmer 1988, with a moderate effect size (0.59-0.75). Not all general stimulation programs are effective. (-1 Risk of Bias; -1 Indirectness). Evidence to Decision Considerations: Programs that work practice real life tasks. Status update since 2013: Newly listed indication.	Low	Weak +	Yellow
		Hadders-Algra 2017[80]	–	–				
		Ziviani 2010[91]	Varied diagnosis<br>0-9 yrs	–				
	135 Improved cognitive skills [BS]	Morgan 2016a[79]	CP & High risk of CP<br>0-2 yrs	Varied	1 RCT. General stimulation program conferred better cognitive skills than NDT by Palmer 1988. (-1 Risk of Bias; -1 Indirectness). Status update since 2013: Newly listed indication.	Low	Weak +	Yellow
77 **Early Intervention – Votja**	136 Improved gross motor skills [A]	Morgan 2016a[79]	CP & High risk of CP<br>0-2 yrs	Varied	2 new systematic reviews. Votja did not confer improved gross motor skills. (-2 Risk of Bias; -1 Indirectness). Evidence to Decision Considerations: Pain affecting acceptability. Substitute interventions exist that confer improved motor skills. Status update since 2013: Quality upgraded from very low to low in this age group. Recommendations for use unchanged.	Low	Weak -	Yellow
		Hadders-Algra 2017[80]	–	–				
78 **Electrical Stimulation**	137 Improved gait parameters, inc walking speed [BS]	Chiu 2014[92]	CP<br><18 yrs	0.5 hr 3x day	5 small RCTs. 3 RCTs compared electrical stimulation v control, and electrical stimulation conferred improved walking speed better than control. 2 RCTs compared electrical stimulation v activity training alone and the treatments were equally effective. (-1 Risk of Bias; -1 Imprecision). Status update since 2013: No change in quality or recommendations for use gradings.	Low	Weak +	Yellow
		Das 2019[65]	–	–	Superseded by Chiu			
		Moll 2017[93]	CP Spastic<br>GMFCS I-III<br>5-19 yrs	1-4 hrs per day	2 RCTs plus other observational evidence. Electrical stimulation conferred improved walking better than control physiotherapy. Importantly, children preferred electrical stimulation to orthotics (-1 Risk of Bias; 1 Imprecision). Status update since 2013: No change in no change in quality or recommendations for use gradings.			
		Salazar 2019[94]	CP Spastic<br>All topographies<br>1-8 yrs	3-6x wk,<br>6-8 wks	6 RCTs. Electrical stimulation conferred better sitting and standing compared to control NDT, but not walking (-1 Risk of Bias; -1 Imprecision). Status update since 2013: No change in no change in quality or recommendations for use gradings.			
	138 Improved strength [BS]	–	–	–	Status update since 2013: No new primary source data, no change in quality or recommendations for use gradings.	Moderate	Weak +	Yellow

**79 Environmental Enrichment**	139 Improved motor skills [A]	Morgan 2013[95]	CP mixed types 0-2 yrs	Varied	7 RCTs. Pooled analysis indicated environmental enrichment conferred small improvements in motor skills [SMD 0.39, 95%CI 0.05–0.72]. (-1 Risk of Bias). Status update since 2013: Newly listed intervention.	Moderate	Strong +	Green
**80 Exercise**	140 Improve-ment in Pulmonary Function Tests [BS]	Blackmore 2019[22]	CP without respiratory conditions 6-12 yrs	“Spirotiger” 15 mins feedback & 30 mins PT 3x wk for 3 wks	1 RCT. Feedback respiratory + conventional PT vs conventional PT. Improvements in FVC and FEV1 in children without existing respiratory disease (-1Risk of Bias, -1 imprecision). Status update since 2013: Newly listed intervention.	Low	Weak +	Yellow
**81 Fitness**	141 Improved fitness [BS]	–	–	–	Status update since 2013: No new primary source data, no change in quality or recommendations for use gradings. Evidence to Decision Considerations: Effective for children GMFCS I-II who can move fast enough to train aerobic fitness. Status update since 2013: No change in no change in quality or recommendations for use gradings.	Moderate	Strong +	Green
	142 Improved participation [A & P]	–	–	–	Status update since 2013: No new primary source data, no change in quality or recommendations for use gradings.	Moderate	Weak -	Yellow
	143 Improved gross motor function [BS & A]	Ryan 2017[96]	CP mixed types GMFCS I-V <19 yrs	Varied. 2-7x wk, 12-60 mins	8 RCTs and Controlled Trials focussed on aerobic capacity, including cycling and treadmill. A pooled analysis of 3 trials indicated, aerobic exercise conferred improved gross motor function short term [SMD 0.53, 95%CI 0.02-1.04] and intermediate term [MD 12.96%, 95%CI 0.52-25.40] (-1 Risk of Bias; -1 Imprecision). Status update since 2013: Newly listed indication.	Low	Weak +	Yellow
	144 Improved gait speed [BS & A]	Ryan 2017[96]	CP mixed types GMFCS I-V <19 yrs	Varied	8 RCTs and Controlled Trials focussed on aerobic capacity. A pooled analysis of 4 trials indicated, aerobic exercise does not improve gait speed short term [MD 0.09m/s, 95%CI −0.11 to 0.28] or intermediate term [MD −0.17m/s, 95%CI −0.59 to 0.24]. (-2 Risk of Bias; -1 Imprecision). Status update since 2013: Newly listed indication.	Very low	Weak +	Yellow
**82 Focal Vibration**	145 Improved kinaesthesia [BS]	Ritzmann 2018[97]	CP 8-15 yrs	FV 70 Hz, 0.5 mm, 10 sec (3x)	1 observational study. Focal vibration may confer improved awareness of joint-position and muscle contraction. (-1 Risk of Bias; -1 Imprecision). Status update since 2013: Newly listed indication.	Very Low	Weak +	Yellow
**83 Goal Directed Training**	146 Improved gross motor [BS & A]	Toovey 2017[98]	CP GMFCS I-III 4-18 yrs	Varied	No new primary source data. Evidence to Decision Considerations: There are now numerous RCTs in the upper and lower limb, plus in early intervention, cognitive orientation to occupational performance, HABIT-ILE and in the home program evidence bases consistently indicating that goal directed training improves goal achievement of functional tasks involving motor performance. Status update since 2013: No change in quality grading for gross motor. We upgraded the recommendations for use from weak positive to strong positive.	Low	Strong +	Green
	147 Improved hand function [BS & A]	–	–	–	Status update since 2013: No new primary source data, no change in quality or recommendations for use gradings.	High	Strong +	Green
	148 Improved self-care [A]	–	–	–	Status update since 2013: No new primary source data, no change in quality or recommendations for use gradings.	High	Strong +	Green
**84 HABIT-ILE**	149 Improved motor function upper & lower limb [BS & A]	Bleyenheuft 2015[99]	CP Unilateral GMFCS I-IV 6-16 yrs	90 hrs in camp format	1 small RCT with cross over. HABIT-ILE conferred improved motor function in both the lower and upper limbs compared to usual care. (-1 Risk of Bias; -1 Imprecision). Status update since 2013: Newly listed intervention.	Low	Weak +	Yellow
		Bleyenheuft 2017[100]	CP Bilateral GMFCS II-IV 6-16 yrs	90 hrs in camp format	1 Controlled Trial. HABIT-ILE conferred improved motor function in both the lower and upper limbs compared to usual care. (-1 Risk of Bias; -1 Imprecision; +1 Large Effects). Status update since 2013: Newly listed intervention.			
		Plasschaert	–	–	Superseded by Bleyenheuft 2017			

		2019[101]						
**85 Hippotherapy**	150 Improved gross motor function [BS & A]	Charry-Sánchez 2018[102]	CP all types GMFCS I-IV 4-12 yrs	30 mins – 1 hr 1-2x wk for 8 wks – 6 mo	3 RCTs with conflicting findings. 1 study found significant improvements in gross motor function whereas other 2 studies found no benefit for CP. (-1 Inconsistency). Status update since 2013: More high-quality evidence exists but no change in recommendation for use grading given the conflicting findings.	Moderate	Weak +	Yellow
		Das 2019[69]	–	–	Superseded by Charry-Sánchez 2018			
		Tseng 2013[103]	–	–	Superseded by Charry-Sánchez 2018.			
	151 Improved fine motor function [BS & A]	MacKinnon 1995[104]	CP spastic GMFCS I-IV 4-12 yrs	1 hr/wk for 6 mo	1 RCT. Hippotherapy conferred improvement fine motor function in only 1 domain (grasping) compared to waitlist control. Best effects observed in moderately severe CP. (-2 Risk of Bias). Status update since 2013: Newly listed indication.	Low	Weak +	Yellow
	152 Improved activities of daily living [A]	MacKinnon 1995[104]	CP spastic GMFCS I-IV 4-12 yrs	1 hr/wk for 6 mo	1 RCT. No between group differences for hippotherapy versus control on activities of daily living. Evidence to Decision Considerations: Cost and feasibility for this indication. It is perhaps unsurprising that hippotherapy does not affect activities of daily living, given there is no overlap in task performance or specificity. Status update since 2013: Newly listed indication.	Low	Weak -	Yellow
	153 Improved balance & posture [BS]	Kwon 2011[105]	4-12 yrs GMFCS I-IV All CP types	30 mins 1-2x wk for 8-12 wks	3 RCTs plus observational studies. Hippotherapy conferred improved balance and posture, specifically improving trunk position and arm function. (-1 Risk of Bias). Evidence to Decision Considerations: Cost and feasibility. Status update since 2013: Upgraded quality from low to moderate, upgraded recommendations for use from weak positive to strong positive.	Moderate	Strong +	Green
		Kwon 2015[106]						
		Matusiak-Wieczorek 2016[107]						
		Dewar 2015[108]						
	154 Improved symmetry [BS]	Charry-Sánchez 2018[102]	CP all types & severities 4-16 yrs	8-30 min/wk for 1-13 wks	3 RCTs. Hippotherapy conferred consistent in muscle symmetry over control. Data suggests a possible dose response and evidence from 1 RCT that controls symmetry got worse. (-1 Risk of Bias; +1 Large Effects). Evidence to Decision Considerations: Improvement in symmetry does not necessarily equate to improvement in function. Status update since 2013: Upgraded quality from low to moderate, upgraded recommendations for use from weak positive to strong positive.	Moderate	Strong +	Green
	155 Decreased muscle spasticity [BS]	Lucena-Antón 2018[109]	CP spastic GMFCS IV-V 3-14 yrs	45 min/wk for 12 wks	1 RCT. Hippotherapy conferred decrease in spasticity on the MAS measure, with a moderate effect size. (-1 Risk of Bias). Evidence to Decision Considerations: Cost and feasibility given effective substitutes for spasticity management exist. Status update since 2013: Newly listed indication not reported in 2013.	Moderate	Weak +	Yellow
	156 Improved participation [P]	–	–	–	Status update since 2013: No new primary source data, no change in quality or recommendations for use gradings.	Moderate	Weak -	Yellow
**86 Hippotherapy Simulation**	157 Improved balance and posture [BS]	Dewar 2015[108]	CP spastic Mixed severity 3-18 yrs CP spastic	Variable (15-60 min, 1-3x wk for 6-12 wks	2 RCTs. Hippotherapy simulation appears to confer better balance and posture than conventional physical therapy or passive abduction. (-1 Imprecision). Evidence to Decision Considerations: 2 RCTs suggest that simulation might be less effective than hippotherapy. Status update since 2013: Newly listed intervention.	Moderate	Weak +	Yellow
		Lee 2014[110]			1 RCT.			
		Temcharoensuk 2015[111]			1 RCT.			
**87 Home**	158 Improved	Novak	CP Mixed	10 mins	2 RCTs. Home programs conferred improved function compared to control no therapy.	Moderate	Strong +	Green

Programs	function [A]	2014[112]	GMFCS I-V 4-13 yrs	daily for 8wks minimum	Evidence to Decision Considerations: Home programs are an effective way to increase the dose of therapy. Status update since 2013: No change in quality or recommendations for use gradings.			
	159 Improved participation [P]	–	–	–	Status update since 2013: No new primary source data, no change in quality or recommendations for use gradings.	Moderate	Weak -	Yellow
88 Hydrotherapy	160 Improved gross motor function [BS & A]	Roostaei 2017[113]	CP mixed types GMCFCS I-V 3-21 yrs	2-3x wk for 6-16 wks	2 RCTs, 9 observational studies (1 RCT is new). 1 RCT indicated hydrotherapy conferred improved gross motor skills, compared to control no therapy, whereas the other RCT found no significant between group differences for hydrotherapy versus land-based physiotherapy. (-1 Risk of Bias; -1 Imprecision). Evidence to Decision Considerations: Facility costs. Low risk of harms. Any gains observed within the water would need to be practiced on land without the assistance of buoyancy, to promote skills transfer and generalisation. Status update since 2013: No change in quality or recommendation for use grading since 2013 review.	Low	Weak +	Yellow
89 Language Therapy	161 Improved interaction [P]	Pennington 2018[114]	Disability including CP 1-8 yrs	8 parent sessions over 12 wks	2 small RCTs. There was no evidence that parent communication training had an effect on children's initiation of conversation or engagement in joint attention during interaction with their mothers. (-2 Risk of Bias; -1 Imprecision). Status update since 2013: No new primary source data, no change in quality or recommendations for gradings.	Very low	Weak +	Yellow
	162 Improved communication [A & P]	Chorna 2016[115]	Mixed populations including CP 0-2 yrs	Varied	5 systematic reviews, 8 RCTs and 4 observational studies. Language therapy promoting parent/caregiver responsivity, pre-linguistic milieu teaching and parent-infant transaction frameworks appeared to confer improved communication. There were no interventions designed exclusively for CP and therefore recommended interventions do not specifically target infants at risk of or with CP. Further research required. (-1 Risk of Bias; -1 Indirectness). Status update since 2013: Newly listed intervention.	Very low	Weak +	Yellow
		Pennington 2016[116]	CP mixed types 3-18 yrs	Varied	An update of their 2004 review. 1 RCT, 2 Controlled Trials, 13 observational studies. 9 were interventions for children, the others were interventions for parents. Language therapy conferred some positive changes in speech, language and communication as well as communication partners. (-1 Risk of Bias; -1 Imprecision). Status update since 2013: Newly listed intervention.			
90 Literacy Interventions	163 Improved reading and literacy [A]	Mandak 2018[117]	CP using AAC 5-18 yrs	3–36 sessions (average 12)	9 observational studies. Literacy interventions conferred improved reading skills with large effect sizes. A range of techniques were used including: speech intelligibility training, letter an sound identification, sound matching, phoneme blending, word segmentation, blending, spelling, and word identification (+1 Large Effect Sizes). Evidence to Decision Considerations: Reading is known to confer improved intelligence in typically developing children and there are harms from not providing the intervention. Status update since 2013: Newly listed intervention.	Moderate	Strong +	Green
		Barker 2012[118]	–	–	Superseded by Mandak 2018.			
	164 Improved literacy via participation in emergent literacy activities [P]	Stauter 2017[119]	CP 3-8 yrs	7 hrs - entire school yr	6 observational studies. Literacy interventions conferred improved emergent literacy skills and classroom participation. The following strategies were consistently effective across the studies: modelling AAC use, adapting materials, subvocalization for decoding, contextual learning, and differentiated instruction. Evidence to Decision Considerations: Children enjoyed the intervention. Reading is known to confer improved intelligence in typically developing children and there are harms from not providing the intervention. We therefore upgraded the strength of the recommendation. Status update since 2013: Newly listed intervention.	Low	Strong +	Green
91 Mirror Therapy	165 Improved motor	Park 2016[120]	CP	Not specified	7 clinical trials non randomized and 2 observational studies. In 7/9 studies mirror therapy conferred improved movement, however the interventions are poorly defined and are not	Very low	Weak +	Yellow

	function [BS & A]				homogenous or operating from the same theoretical constructs, and 3/9 studies compared CP to normal controls. (- 2 Risk of Bias; -1 Indirectness; -1 Imprecision). Status update since 2013: Newly listed intervention.			
	166 Improved tactile perception [BS]	Auld 2017[121]	CP<br>MACS I-III<br>6-18 yrs	90 mins x1	1 small RCT with cross over. Mirror□based training conferred improved tactile perception improved in 4 of 6 participants but was unchanged with control bimanual therapy. (- 2 Risk of Bias). Status update since 2013: Newly listed intervention.	Very low	Weak +	Yellow
**92 Mobility Training**	167 Improved gross motor function [BS & A]	Bania 2018[122]	CP<br>GMFCS I-III<br>2-18 yrs	Variable 240-3600 mins	9 RCTs. No between group differences for improvement in gross motor function from mobility training for any intervention used. Eclectic group of interventions including Nintendo; wall climbing, sit to stand, and circuit training of functional tasks. (-1 Indirectness; -1 Inconsistency). Evidence to Decision Considerations: Low risk of harms. Status update since 2013: Newly listed intervention.	Low	Weak +	Yellow
		Booth 2018[123]	CP<br>GMFCS I-IV<br>4-21 yrs	Variable 108-1800 mins	11 RCTs. Pooled analysis of all types of mobility training did not show a positive effect on gross motor function. (-1 Indirectness; -1 Inconsistency). Evidence to Decision Considerations: Low risk of harms. Status update since 2013: Newly listed intervention.			
		Clutterbuck 2018[124]	CP<br>GMFCS I-III<br>2-18 yrs	Variable 8-55 hrs	4 RCTs. Gross motor activity training conferred inconsistent improvement in gross motor function. (-1 Indirectness; -1 Inconsistency). Evidence to Decision Considerations: Low risk of harms. Status update since 2013: Newly listed intervention.			
		Elnahhas 2019[125]	CP hemiplegia<br>GMFCS I-II<br>10-14 yrs	15-25 mins, 3x wk for 6-12 wks	1 RCT. Backward gait training conferred better improvements in gross motor function (with 3-9% gains) than forward training. (-1 Indirectness). Evidence to Decision Considerations: Low risk of harms. Status update since 2013: Newly listed intervention.			
		Paleg 2015[126]	CP<br>GMFCS III-IV<br>5-18 yrs	30 min, 2x wk for 9 wks	1 RCT, plus observational studies. No between group differences for body weight supported treadmill compared to overground walking. (-1 Indirectness). Status update since 2013: Newly listed intervention.			
	168 Improved walking speed [BS]	Booth 2018[123]	CP<br>GMFCS I-IV<br>4-21 yrs	Variable 108-1800 mins	11 RCTs. Pooled data Indicated improved walking speed from mobility training. A greater effect occurred when gait training was enhanced withy auditory or visual feedback or tDCS. (-1 Indirectness). Status update since 2013: Newly listed intervention.	Moderate	Strong +	Green
		Moreau 2016[127]	CP<br>4-21 yrs	Variable 8-36 sessions	Meta-analysis of 7 RCTs and 1 controlled trial. All types of gait training conferred improved walking speed with a large effect size (0.92), with the analysis strongly weighted strongly towards treadmill training or mechanical assisted gait training. (-1 Indirectness). Status update since 2013: Newly listed intervention.			
		Paleg 2015[126]	CP and ID<br>2-16 yrs<br>GMFCS IV-V	Variable	1 RCT, plus observational studies. Gait trainers may assist a child GMFCS IV-V to take more steps but does not increase their speed. Evidence to Decision Considerations: High acceptability as it gives the child the experience of walking. Status update since 2013: Newly listed intervention.	Very low	Weak +	Yellow
**93 Modified Sport**	169 Improved gross motor function [BS & A]	Clutterbuck 2018[124]	Spastic CP<br>GMFCS I-III<br>4-16 yrs	1-1.5 hr/wk for 4-10 wks	2 observational studies. Modified sport conferred improved gross motor skills compared to control. (-1 Risk of bias). Status update since 2013: Newly listed intervention.	Very low	Weak +	Yellow
		Chappell 2019[128]	CP	_	1 observational study. Modified sport conferred improved gross motor skills compared to control. (-1 Risk of bias). Status update since 2013: Newly listed intervention.			
	170 Improved gait [BS]	Clutterbuck 2018[124]	CP all types<br>GMFSC I-III<br>4-16 yrs	45-90 mins/wk	3 observational studies. Modified sport conferred improved running speed, agility, velocity and gait (length and velocity). (-1 Risk of Bias). Status update since 2013: Newly listed intervention.	Very low	Weak +	Yellow
		Chappell 2019[128]	CP	_	4 observational studies. Modified sport conferred improved running speed, agility, velocity and gait (length and velocity). (-1 Risk of Bias). Status update since 2013: Newly listed intervention.			
	171 Improved	Chappell	CP diplegia	2x per wk for	1 observational study. Modified sport conferred improved aerobic fitness (with a more	Very low	Weak +	Yellow

	fitness [BS]	2019[128]	GMFCS I<br>16 yrs	10 wks	than minimum clinically meaningful difference) (-1 Risk of Bias). Evidence to Decision Considerations: Only milder forms of CP (e.g. GMFCS I) can exercise at sufficient speed to affect cardiovascular fitness, findings cannot be generalised to more severe physical disability. Status update since 2013: Newly listed intervention.			
**94 Motor speech interventions**	172 Improved speech intelligibility [BS & P]	Pennington 2016[116]	CP mixed types<br>3-8yrs	Varied	0 RCTs. 48 observational studies. Speech therapy using motor learning principles may confer improved speech intelligibility, voice quality and clarity, but more research is needed. (-1 Risk of Bias, - 1 Imprecision). Evidence to Decision Considerations: High acceptability but high cost.	Very low	Weak +	Yellow
**95 Neurodevelop mental Therapy [NDT] / Bobath**	173 Improved gross motor function [BS & A]	Zanon 2019[129]	CP diplegia<br>GMFCS I-III<br>3-15 yrs	2-18 hr wk for 6-16 wks	3 new RCTs with meta-analysis of 1 RCT. NDT is no better than control usual care for improving gross motor skills. [MD 1.40, 95%CI -5.47 to 8.27]. (-1 Risk of Bias; -1 Imprecision). These findings confirm 3 previous systematic reviews. The authors concluded "the effectiveness of neurodevelopmental treatment for children with CP is unclear. Because of the lack of good certainty evidence, neurodevelopmental treatment should be used with caution. The child's response should be observed carefully". Evidence to Decision Considerations: Effective substitute interventions exist for improving gross motor function and therefore the recommendation was downgraded. Status update since 2013: Newly listed indication, gross motor function was not previously reported.	Low	Strong -	Red
		Labaf 2015[130]	CP<br>2-6 yrs		1 Controlled Trial. No between group differences for NDT v control for walking, running, jumping on the GMFM-88. NDT conferred better lying, rolling, sitting and crawling on the GMFM-88 (-2 Risk of Bias; -1 Imprecision). Evidence to Decision Considerations: It is unclear if better lying, rolling, and crawling is clinically meaningful in children aged 2-6 yrs. Effective substitute interventions exist for improving gross motor function. Status update since 2013: Newly listed indication, gross motor function was not previously reported.			
		Dewar 2015[108]	CP		Did not compare NDT to control no therapy but rather a variety of different therapies and therefore no new primary source data.			
	174 Normalised movement [BS]	Batra 2012[131]	CP	40 mins 3x wk for 3 mo	1 small RCT. Neurofacilitation of Developmental Reaction (NFDR) approach conferred better normalised movement than NDT. NFDR involved preparatory movements and postural control. NDT involved positioning and handling using key points of control + stretching. (-2 Risk of Bias). Status update since 2013: No change in quality or recommendations for use gradings.	Low	Strong -	Red
	175 Prevent contracture [BS]	–	–	–	Status update since 2013: No new primary source data, no change in quality or recommendations for use gradings.	Low	Strong -	Red
	176 Improved function [A]	Ari 2017[132]	CP diplegia<br>GMFCS I-III<br>3-10 yrs	45 mins 2x wk for 6 wks	1 new RCT. No between group differences for NDT + usual care v usual care alone on Timed Up and Go Test. (-1 Risk of Bias). Evidence to Decision Considerations: Effective substitute interventions exist for improving function and therefore the recommendation was downgraded. Status update since 2013: Quality was upgraded from low to moderate. Recommendations for use were downgraded from weak negative to strong negative.	Moderate	Strong -	Red
	177 Improved social emotional [PF]	–	–	–	Status update since 2013: No new primary source data, no change in quality or recommendations for use gradings.	Low	Strong -	Red
	178 Reduced spasticity [BS]	Ari 2017[132]	CP diplegia<br>GMFCS I-III<br>3-10 yrs	45 mins 2x wk for 6 wks	1 new RCT. NDT + usual care conferred reduced spasticity compared to usual care alone on the Modified Ashworth Scale. (-1 Risk of Bias; -1 Indirectness). Evidence to Decision Considerations: Effective substitute interventions exist for reducing spasticity and this intervention is very indirect. Status update since 2013: Newly listed indication.	Low	Weak +	Yellow
**96 Non-speech oral motor**	179 Improved verbal	–	–	–	Status update since 2013: No new primary source data, no change in quality or recommendations for use gradings.	N/A	Weak -	Yellow

exercises	speech [BS]							
97 **Oral Sensorimotor Treatment**	180 Improved physiological mechanism for swallow [BS]	Morgan 2012[133]	CP spastic, athetoid, mixed moderate-severe 4-21 yrs	5-40 mins day for 9-20 wks	3 RCTs, 2 of which included children with CP. No between group differences for oral sensorimotor therapy compared to standard care. 1 RCT compared sensorimotor treatment v chewing treatment v increasing food texture resistance + 10 weeks of sensorimotor treatment, where all three treatments are oral sensorimotor treatments meaning it is unsurprising there was no between group differences. (-1 Risk of Bias; -1 Indirectness; -1 Imprecision). Status update since 2013: Quality ratings unchanged. Recommendations for use upgrade from weak negative to weak positive.	Very low	Weak +	Yellow
		Arvedson 2010[134]	–	–	Superseded by Morgan 2012.			
	181 Improved diet consistency [BS]	Morgan 2012[133]	CP spastic, athetoid, mixed moderate-severe 4-21 yrs	5-40min day for 9-20 wks	3 RCTs, 2 of which included children with CP. No between group differences for diet consistency and texture variation compared to standard care. (-1 Risk of Bias; -1 Indirectness; -1 Imprecision). Status update since 2013: Newly listed indication.	Very low	Weak +	Yellow
	182 Improved growth [BS]	Morgan 2012[133]	CP spastic, athetoid, mixed moderate-severe 4-21 yrs	5-40min day for 9-20 wks	3 RCTs, 2 of which included children with CP. No statistically significant between group differences for growth and texture variation compared to standard care. (-1 Risk of Bias; -1 Indirectness; -1 Imprecision). Status update since 2013: Newly listed indication.	Very low	Weak +	Yellow
		Arvedson 2010[134]	–	–	Superseded by Morgan 2012.			
	183 Improved oral motor function [BS]	Sigan 2013[135]	CP 1-4 yrs	1hr wk for 6 mo	1 RCT. Oral motor training conferred improved chewing, swallowing and sialorrhea compared to control. (-1 Risk of Bias). Evidence to Decision Considerations: Poor feeding is a very important clinical problem. Status update since 2013: Newly listed indication.	Moderate	Strong +	Green
	184 Improved feeding skills [A]	Khamis 2019[136]	CP <12 mo	Varied: 3 sessions, 1-2 weekly, daily	1 historical case control, 4 observational studies. Combination of motor learning interventions plus compensatory strategies improved feeding. (-1 Risk of Bias; -1 Low sample sizes; - 1 Indirectness; -1 Imprecision)	Very low	Weak +	Yellow
		Mlinda 2018[78]	CP spastic, hypotonic, mixed moderate-severe 2 yrs	6-8 parent education sessions + 6-8 OT sessions	1 RCT. No between group differences for focused parent education on feeding compared to control general information about nutrition, but within group improvements observed (-1 Risk of Bias; -1 Indirectness; -1 Imprecision). Evidence to Decision Considerations: Malnutrition is a very important clinical problem, but the findings are inconsistent across studies for varying techniques and therefore we downgraded the strength of the recommendation for use. Status update since 2013: Newly listed indication.	Moderate	Weak +	Yellow
		Sigan 2013[135]	CP 1-4 yrs	1hr wk for 6 mo	1 RCT. Oral motor training conferred improved feeding skills compared to control. (-1 Risk of Bias). Status update since 2013: Newly listed indication.			
		Arvedson 2010[134]	CP spastic, athetoid, mixed moderate-severe 4-21yrs.	5-40 min day for 9-20 wks	4 observational studies. Innsbruck Sensorimotor Activator and Regulator (ISMAR) conferred improved functional swallowing. (-1 Risk of Bias; -1 Indirectness; -1 Imprecision). Status update since 2013: Newly listed indication.			
		Morgan 2012[133]	CP spastic, athetoid, mixed moderate-severe 4-21yrs	5-40 min day for 9-20 wks	3 RCTs, 2 of which included children with CP. No between group differences for oral sensorimotor therapy compared to standard care for feeding skills. (-1 Risk of Bias; -1 Indirectness; -1 Imprecision). Status update since 2013: Newly listed indication.			
	185 Improved chewing function [A]	Morgan 2012[133]	CP spastic, athetoid, mixed moderate-severe 4-21 yrs	5-40 min day for 9-20 wks	3 RCTs, 2 of which included children with CP. Oral sensorimotor therapy conferred improved chewing skills. (-1 Risk of Bias). Evidence to Decision Considerations: Chewing is an important clinical problem to solve. Status update since 2013: Newly listed indication.	Moderate	Strong +	Green
		Inal 2017[137]	CP	5 x 20 min	1 RCT. Functional chewing training (FuCT) conferred better chewing compared to control			

			GMFCS II-IV<br>Mean 4 yrs	for 12 wks	traditional oral sensorimotor therapy (positioning, sensory stimulation, chewing exercise & adaptive food consistency). (-1 Risk of Bias).			
	186 Reduced tongue thrust [BS]	Inal 2017[137]	CP<br>GMFCS II-IV<br>Mean 4 yrs	5 x 20 min for 12 wks	1 RCT. Functional chewing training (FuCT) conferred reduced tongue thrust compared to control traditional oral sensorimotor therapy (positioning, sensory stimulation, chewing exercise & adaptive food consistency). (-1 Risk of Bias). Status update since 2013: Newly listed indication.	Moderate	Weak +	Yellow
	187 Improved feeding behaviours [A & P]	Inal 2017[137]	CP<br>GMFCS II-IV<br>Mean 4 yrs	5 x 20 min for 12 wks	1 RCT. Functional chewing training (FuCT) conferred improved feeding behaviours compared to control traditional oral sensorimotor therapy (positioning, sensory stimulation, chewing exercise & adaptive food consistency). (-1 Risk of Bias; -1 Indirectness). Status update since 2013: Newly listed indication.	Low	Weak +	Yellow
	188 Reduced sialorrhea [BS]	Inal 2017[137]	CP<br>GMFCS II-IV<br>Mean 4 yrs	5 x 20 min for 12 wks	1 RCT. Functional chewing training (FuCT) conferred better reduced sialorrhea severity than control traditional oral sensorimotor therapy (positioning, sensory stimulation, chewing exercise & adaptive food consistency), suggesting oral sensorimotor therapy is less effective than motor learning training approaches. (-1 Risk of Bias; -1 Indirectness). Evidence to Decision Considerations: Sialorrhea is a very important clinical problem, but the findings are inconsistent across studies for varying techniques and therefore we downgraded the strength of the recommendation for use. Status update since 2013: Newly listed indication.	Moderate	Weak +	Yellow
		Sigan 2013[135]	CP<br>1-4 yrs	1hr wk for 6 mo	1 RCT. Oral motor training conferred better reduced sialorrhea than control physiotherapy. (-1 Risk of Bias). Evidence to Decision Considerations: Downgrade for inconsistencies in the literature.			
		Arvedson 2010[134]	CP<br>1-2 yrs	Chin cup	5 observational studies, 1 study used a chin cup. Oral sensorimotor therapy incorporating chin cups had very little to no effect on sialorrhea (-1 Risk of Bias; -1 Indirectness; -1 Imprecision).			
**98 Oral Sensorimotor Treatment + Electrical Stimulation**	189 Reduced sialorrhea [BS]	Umay 2019[138]	CP<br>2-6 yrs	30 min day 5x wk for 4 wks	1 RCT. Electrical stimulation + oral sensorimotor therapy conferred better sialorrhea reduction than sham electrical stimulation + oral sensorimotor therapy. Evidence to Decision Considerations: Safety is not well studied, and professional bodies caution its use in children, mild discomfort (head turning) but no harms reported. Status update since 2013: Newly listed intervention.	High	Strong +	Green
	190 Improved feeding skills [A]	Umay 2019[138]	CP<br>2-6 yrs	30 min day 5x wk for 4 wks	1 RCT. Electrical stimulation + oral sensorimotor therapy conferred better lip closure while swallowing, ability to swallow food without excess loss, ability to sip liquid, ability to swallow liquid without excess loss, and ability to swallow without cough than sham electrical stimulation + oral sensorimotor therapy. Evidence to Decision Considerations: Safety is not well studied, and professional bodies caution its use in children, mild discomfort (head turning) but no harms reported. Status update since 2013: Newly listed intervention.	High	Strong +	Green
		Song 2015[139]	CP<br>3-8 yrs	30min 2x wk for 8wks	1 RCT. Electrical stimulation + oral sensorimotor therapy conferred better feeding skills than sham electrical stimulation + oral sensorimotor therapy. (-1 Risk of Bias). Status update since 2013: Newly listed intervention.	Moderate	Weak +	Yellow
**99 Orthotics | Upper Limb**	191 Improved hand function [BS & PF]	Jackman 2013[140]	CP<br><18 yrs	Varied	6 RCT's, 5 included static splinting and 1 functional splinting. Static splints plus therapy conferred small improvements in hand function compared to therapy alone. More research is needed about functional splints (-1 Imprecision). Evidence to Decision Considerations: Costs, low acceptability from discomfort. Status update since 2013: Quality upgraded form very low to moderate. Recommendations for use upgraded from weak negative to weak positive.	Moderate	Weak +	Yellow
		Garbellini, 2018[141]	–	–	Superseded by Jackman 2013.			
**100 Parent**	192 Improved	–	–	–	Status update since 2013: No new primary source data, no change in quality or	Very low	Weak +	Yellow

**Education**	parenting skills to facilitate development [EF]				recommendations for use gradings. Evidence to Decision Considerations: Refer also to Stepping Stones Triple P.			
101 **Physical Activity Interventions [includes exercise, activity training, strength/endurance training, behaviour therapy, balance]**	193 Improved physical activity [BS & A]	Reedman 2017[142]	CP<br>GMFCS I-V<br><25 yrs	Varied	7 RCTs, 2 observational studies. Physical training, activity level training, physical training + behaviour change therapy, online behaviour change modules, and context-focused therapy conferred a small effect on physical activity intervention compared with passive usual care. Habitual physical activity, increased by approximately 1000 steps per day [SMD 0.34, 95%CI 0.03–0.66]. (-1 Risk of Bias; -1 Imprecision). Status update since 2013: Newly listed intervention	Low	Weak +	Yellow
		Bloemen 2017[143]	Physical disability	Varied	5 RCTs, 2 observational studies. Almost the same source data as Reedman but the interventions were categorised differently. No treatment effect for physical training alone. Whereas physical training + a behavioural component had conflicting results, with some studies indicating increased step count, but gains were not maintained at follow up. Parents reported children were more active (-1 Risk of Bias; -1 Imprecision). Status update since 2013: Newly listed intervention			
		O'Brien 2016[144]	Physical disability including CP Wheelchair users<br>GMFCS IV-V<br><25 yrs	4x wk	31 studies - 4 RCTs & 6 observational studies, focusing on children who were wheelchair users. Physical activity interventions conferred improved health, fitness and well-being, without adverse outcomes compared to control (-2 Risk of Bias). Status update since 2013: Newly listed intervention	Low	Weak +	Yellow
	194 Improved ambulation & mobility [A]	O'Brien 2016[144]	Physical disability including CP Wheelchair users<br>GMFCS IV-V<br><25 yrs	4x wk	31 studies - 4 RCTs & 6 observational studies, focusing on children who were wheelchair users. Conflicting results. For the most part, studies indicated physical activity interventions did not improve walking and ambulation, however some studies showed positive effects (-1 Risk of Bias; -1 Imprecision). Evidence to Decision Considerations: Given the participants were wheelchair users, the clinical relevance of measuring change in walking and ambulation is questionable. Status update since 2013: Newly listed intervention	Low	Weak +	Yellow
	195 Improved gross motor function [BS & A]	Ryan 2017[96]	CP mixed types<br>GMFCS I-V <19 yrs	Varied. 2-7x wk, 12-60 mins	29 Trials. Mixed physical activity training did not improve gross motor function [SMD0.02, 95%CI −0.29 to 0.33] or gait speed [MD 0.10 m/s, −0.07 to 0.27]. Status update since 2013: Newly listed intervention	Low	Weak -	Yellow
		O'Brien 2016[144]	Physical disability including CP Wheelchair users<br>GMFCS IV-V<br><25 yrs	Varied	31 studies - 4 RCTs & 6 observational studies, focusing on children who were wheelchair users. Conflicting results. Predominantly studies indicated physical activity interventions did not improve gross motor functions, and any study with gains the improvements were lost at follow up (-1 Risk of Bias; -1 Imprecision). Status update since 2013: Newly listed intervention	Low	Weak -	Yellow
	196 Improved fitness [BS]	O'Brien 2016[144]	Physical disability including CP Wheelchair users<br>GMFCS IV-V<br><25 yrs	4x wk	31 studies - 4 RCTs & 6 observational studies, focusing on children who were wheelchair users. Conflicting results. Some studies showed gains short term. Predominantly studies indicated physical activity interventions did not improve fitness. (-1 Risk of Bias; -1 Imprecision). Status update since 2013: Newly listed intervention	Low	Weak +	Yellow
	197 Improved participation in physical activities [P]	Reedman 2017[142]	CP spastic<br>GMFCS I-III<br>7-22 yrs	Varied.<br>30-60 mins<br>1-6x wk for 4-39 wks	3 studies. Reedman found mixed physical activities did not have an effect on leisure participation [SMD 0.40, 95%CI -0.40 to 1.19]. Whereas Ryan found mixed physical activity training improved participation [MD 0.40, 95% CI 0.13 to 0.67, N = 65, 1 study] in the short-term. Note: there were 6 adverse events reported across all studies in this review (muscle pain, falls/accidents, broken bone). (-1 Risk of Bias; -1 Imprecision). Status update since 2013: Newly listed intervention	Moderate	Weak +	Yellow
		Ryan 2017[96]	CP mixed types<br>GMFCS I-V <19	Varied. 2-7x wk, 12-60				

			yrs	mins				
	198 Improved quality of life [BS, A, P, EF, PF]	O'Brien 2016[144]	Physical disability including CP Wheelchair users GMFCS IV-V <25 yrs	Varied	31 studies - 4 RCTs & 6 observational studies, focusing on children who were wheelchair users. Conflicting results. Physical activity (walking, cycling, PRT, functional exercises) probably conferred improved quality of life, especially if the intervention included more physical fitness components. (-1 Risk of Bias; -1 Imprecision). Status update since 2013: Newly listed intervention	Low	Weak +	Yellow
**102 Play Therapy**	199 Improved play skills [A]	Hsieh 2012[145]	CP 6-8 yrs	10 sessions	1 new observational study comparing the play in children with CP to typically developing children. Pretend play (adapted to accommodate physical disability) appeared to confer improved positive affect and imagination. Status update since 2013: No change in quality or recommendation for use grading since 2013 review.	Low	Weak +	Yellow
	200 Improved coping & reduced stress [BS & PF]	–	–	–	Status update since 2013: No new primary source data, no change in quality or recommendations for use gradings.	N/A	Weak +	Yellow
**103 Postural Management**	201 Prevention hip displace-ment [BS]	Gmelig 2018[146]	CP GMFCS III-V 0-18 yrs	Varied	8 observational studies. Position a child in hip abduction in either standing fame or standing shell or sitting program may slow the rate of hip subluxation (-1 Indirectness; -1 Imprecision). Evidence to Decision Considerations: Children can experience discomfort and restricted mobility. The decision to recommend 24 hr postural management and age at start of intervention should be made with caution. Status update since 2013: Newly listed indication.	Very low	Weak +	Yellow
		Miller 2017[147]	CP GMFCS II-V	Varied	8 observational studies, 6/8 positioning the child in hip abduction. 2 studies indicated positioning devices (including standing frames, customised seating devices, and sleep positioning systems) may have improved hip displacement, whereas 4 studies had no effect (-1 Imprecision). Evidence to Decision Considerations: Management of hip displacement requires a comprehensive approach. Longitudinal population-based studies in Sweden have demonstrated that the most effective method for preventing hip displacement is regular hip surveillance, with comprehensive multidisciplinary management, at the right time. Status update since 2013: Newly listed indication.			
**104 Pressure Care**	202 Reduced ulcer develop-ment from mattresses [BS]	–	–	–	Status update since 2013: No new primary source data, no change in quality or recommendations for use gradings.	Low	Strong +	Green
	203 Reduced ulcer develop-ment from wheelchair cushions [BS]	–	–	–	Status update since 2013: No new primary source data, no change in quality or recommendations for use gradings.	Low	Weak +	Yellow
**105 Respite**	204 Reduced parent stress [EF]	–	–	–	Status update since 2013: No new primary source data, no change in quality or recommendations for use gradings.	Low	Weak +	Yellow
**106 Sensation Training**	205 Improved tactile function via	McLean 2017[148]	CP Hemiplegia	3x wk for 6 wks	1 pilot RCT published after the Auld 2014 systematic review. Authors conclude sensation training conferred improved goal performance, proprioception, and bimanual hand use, better than control goal directed training (-1 Risk of Bias; -1 Imprecision). Status update	Very low	Weak +	Yellow

		tactile training [BS]				since 2013: Newly listed intervention			
107	**Sensory Integration**	206 Improved sensory organisation [BS]	–	–	–	Status update since 2013: No new primary source data, no change in quality or recommendations for use gradings.	Low	Strong -	Red
		207 Improved motor skills [BS & A]	–	–	–	Status update since 2013: No new primary source data, no change in quality or recommendations for use gradings.	Low	Strong -	Red
		208 Improved tactile function [BS]	Auld 2014[149]	CP Diplegia	1.5 hrs per day for 3 wks	1 small 3-group RCT. No between group difference for improving sensation from sensory integration of sensory perceptual motor training, however the study is underpowered and not directly dedicated to sensation training (-1 Risk of Bias; -1 Indirectness; -1 Imprecision). Status update since 2013: Newly listed intervention	Very low	Weak -	Yellow
108	**Sensory Processing**	209 Improved function [A]	–	–	–	Status update since 2013: No new primary source data, no change in quality or recommendations for use gradings.	N/A	Weak -	Yellow
109	**Sleep Hygiene**	210 Improved sleep onset [BS]	Rigney 2018[150]	CP	Daily	1 observational study for CP. Sleep hygiene interventions confer improved sleep onset. (-1 Imprecision). Evidence to Decision Considerations: Poor sleep affects negatively academic performance and behaviour, therefore there is a harm in not acting. Status update since 2013: Newly listed intervention	Very low	Weak +	Yellow
110	**Social Stories**	211 Improved communication & behaviour [A]	–	–	–	Status update since 2013: No new primary source data, no change in quality or recommendations for use gradings.	Very low	Weak +	Yellow
111	**Solution Focussed Brief Therapy**	212 Reduced parental depression [EF]	–	–	–	Status update since 2013: No new primary source data, no change in quality or recommendations for use gradings.	N/A	Weak +	Yellow
112	**Stepping Stones Triple P**	213 Child behaviour [BS]	Whittingham 2016[19]	Parents of children with CP 2-12 yrs	6 x 2 hr groups + 3x 30 mins phone call	1 RCT. Stepping Stones Triple P + ACT conferred decreased behavioural problems and intensity, hyperactivity and parental over reactivity. SSTP alone was linked to decreased behavioural and emotional problems. These gains were maintained at follow up. (-1 Risk of Bias). Status update since 2013: Quality upgraded from low to moderate. Recommendations for use upgraded from weak positive to strong positive.	Moderate	Strong +	Green
		214 Reduced parenting stress [EF]	Whittingham 2016[19]	Parents of children with CP 2-12 yrs	6 x 2 hr groups + 3x 30 mins phone call	1 RCT. Stepping Stones Triple P + ACT conferred reduced parental stress compared to waitlist control. (-1 Risk of Bias). Status update since 2013: Quality upgraded from very low to moderate. Recommendations for use upgraded from weak positive to strong positive.	Moderate	Strong +	Green
		215 Improved quality of life [BS, A, P, EF, PF]	Whittingham 2016[19]	Parents of children with CP 2-12 yrs	6 x 2 hr groups + 3x 30 mins phone call	1 RCT. Stepping Stones Triple P + ACT conferred improved quality of life on CP-QOL compared to waitlist control. (-1 Risk of Bias; -1 Indirectness). Status update since 2013: Newly listed indication.	Low	Weak +	Yellow
		216 Improved mobility [A]	Whittingham 2016[19]	Parents of children with CP 2-12 yrs	6 x 2 hr groups + 3x 30 mins phone call	1 RCT. Stepping Stones Triple P + ACT conferred improved mobility on the PEDI compared to waitlist control. (-1 Risk of Bias; -1 Indirectness). Status update since 2013: Newly listed indication.	Low	Weak +	Yellow
113	**Strength Training**	217 Improved lower limb strength	Park 2013[151]	CP primarily spastic diplegic 6.3-15.9 yrs	25 – 70 mins sessions, 3 x per wk, 5-	13 RCTs combined in a meta-analysis. Strength Training conferred improved muscle strength and gait. (-1 Imprecision). Evidence to Decision Considerations: An optimal training protocol has not been identified, but an ideal dose threshold of 3 sessions per	Moderate	Strong +	Green

	[BS]			12 wks (varied by RCT)	week is recommended. Upgraded for large effect sizes. Status update since 2013: Upgraded from weak positive to strong positive.			
		Abbashkhanian 2015[152]	–	–	Superseded by Park 2013			
	218 Improved upper limb strength [BS]	Rameckers 2014[153]	CP 8.9-20 yrs	3x per wk, 6-8 wks	3 small RCTs and 3 observational studies. Strength Training confers improved muscle strength and counteracts the destructive effects of BoNT injection compared to control interventions. (-1 Risk of Bias; - 1 Imprecision). Status update since 2013: No change in grading	Low	Weak +	Yellow
	219 Improved muscle strength [BS]	Gillett 2016[154]	CP GMFCS I-III 3.4 yrs	3 x per wk for 6 wks	1 small RCT. Focal vibration may confer improved muscle structure following strength training. Robust RCTs are lacking. (-1 Risk of Bias; -1 Imprecision). Status update since 2013: Newly listed indication.	Low	Weak +	Yellow
	220 Improved function [A]	Rameckers 2014[153]	CP 8.9-20 yrs	3x per wk, 6-8 wks	3 small RCTs. Strength Training may confer improved function compared to control, however there was insufficient data to draw conclusions. (1 Risk of Bias; - 1 Imprecision; - 1 Indirectness). Status update since 2013: No change in grading	Very Low	Weak +	Yellow
	221 Improved gross motor function [BS & A]	Park 2013[151]	CP primarily spastic diplegic 6.3-15.9 yrs	25 – 70 mins sessions, 3 x per wk, 5-12 wks	13 RCTs combined in a meta-analysis. Strength Training conferred improved gross motor function with moderate effect sizes (d=0.668). The authors conclude that there is some improvement in activity, though more research needs to conduct. (-1 Imprecision). Status update since 2013: Newly listed indication.	Moderate	Weak +	Yellow
**114 Stretching**	222 Reduced spasticity [BS]	Eldridge 2016[155]	CP all types 1-7 yrs	30 mins daily	3 RCTs, 2 RCTs used same cohort. Note this is not new primary source data since our 2013, rather it is reanalysed by different systematic review authors in another way. There is no between group difference favouring manual stretching for reducing spasticity. (-2 Risk of Bias; -1 Imprecision). Evidence to Decision Considerations: Discomfort to children and manual stretching is onerous for carers out at 30 mins/day or longer and therefore may affect feasibility/acceptability and negatively affect the parent-child relationship. Manual stretching is a local treatment for a central nervous system problem, and it is therefore unsurprising spasticity was not reduced. Effective spasticity management substitutes exist and should be preferentially used and thus we downgraded to weak negative. Status update since 2013: Newly listed indication.	Very low	Weak -	Yellow
	223 Contracture prevention &/or improved range of motion via manual stretching [BS]	Eldridge 2016[155]	CP all types 1-7 yrs	5 stretches	1 observational study, with new primary source data. Manual stretching (either by a physiotherapist or self-stretching 5x) conferred immediate 10 degrees of improvement in passive range of motion, but this was not sustained long term. Authors suggest this might lead to long term adaptations if performed regularly but did not have data to support this hypothesis. Evidence to Decision Considerations: Discomfort to children may affect feasibility and acceptability. Ten degrees of improvement is may or may not be clinically meaningful dependent of the child's range. Given stretch can transiently loosen muscle resistance, it is not surprising short-term gains are observed but not sustained given the central messages of spasticity / overactivity are present 24 hrs/day. Status update since 2013: No change in grading	Low	Weak -	Yellow
	224 Contracture prevention via splinting or positioning [BS]	–	–	–	Status update since 2013: No new primary source data, no change in quality or recommendations for use gradings.	Low	Weak +	Yellow
**115 Suit Therapy/ Garment**	225 Improved gross motor	Wells 2018[156]	CP mixed types, primarily spastic	2-12 hrs/day for 3-12 wks	5 small RCTs and observational studies, with 3 studies pooled into a meta-analysis. No between group differences for suit therapy compared to control NDT for improving gross	Moderate	Weak -	Yellow

**Therapy** There are many different types of suits including: TheraSuit, Second Skin, the AdeliSuit, Thera Togs, UpSuit, Camp Lycra Dynamic Elastomeric Fabric Orthose (DEFO), Full Body Suit (FBS), Space Suit, PediaSuit, Penguin Suit, Compression clothing, Suit therapy, Bungy Suit, NeuroSuit.	function [BS & A]		15 mo – 17 yrs		motor function. (-1 Imprecision). Evidence to Decision Considerations: Some children disliked wearing the suits and experienced adverse events including respiratory compromise, overheating and peripheral cyanosis. The suits also impeded function such as independent toileting and dressing. We have downgraded the recommendations based on these side effects to weak negative. Effective substitutes exist for improving gross motor skills. For children that do not experience side effects, suit therapy may be an option. Status update since 2013: The quality of the evidence was upgraded from low to moderate but there was no change in grading of the recommendations for use.			
		Martins 2015[157]	CP mixed types 6 yrs, 11 mo	2-12 hrs/day for 3-12 wks	This meta-analysis included 1 additional RCT (Mahani et al. 2011) to Wells 2018 but concluded the opposite to Wells 2018 findings. We therefore analysed the individual RCTs and identified a mathematical flaw in Martins meta-analyses. Martins erroneously counted the experimental sample group twice which falsely inflated the treatment effect, and therefore we assessed the true effect to be 4 RCTs showing no between group difference between suit therapy and control. In Mahani's RCT, there was a trend to the group that received modified suit therapy + functional training without a suit, having superior outcomes for gross motor function. Evidence to Decision Considerations: Mahani's finding is consistent with the idea that the functional training component of the combined treatment was effective. Status update since 2013: The quality of the evidence was upgraded from low to moderate but there was no change in grading of the recommendations for use.			
	226 Proximal Kinematics [BS]	Wells 2018[156]	CP mixed types primarily spastic 15 mo – 17 yrs	2-12 hrs/day for 3-12 wks	2 RCTs. Suit therapy conferred short-term benefit on proximal kinematics compared to control NDT. The authors noted that improved proximal kinematics did not translate into an improvement in function. (-2 Risk of Bias). Evidence to Decision Considerations: Well's finding is consistent with the idea that the suit might act on hip and shoulder stability and movement, given the suit is located over the hips and shoulders, whereas there was no effect on distal kinematics as the suit could not act on regions of the body not covered by the suit. Some children disliked wearing the suits and experienced adverse events including respiratory compromise, overheating and peripheral cyanosis. The suits also impeded function such as independent toileting and dressing. We have downgraded the recommendations based on these side effects to weak negative. Status update since 2013: Newly listed indication.	Low	Weak -	Yellow
	227 Distal Kinematics [BS]	Wells 2018[156]	CP mixed types primarily spastic 15 mo – 17 yrs	2-12 hrs/day for 3-12 wks	2 RCTs. No between group differences for suit therapy compared to control NDT for distal kinematics (-2 Risk of Bias). Evidence to Decision Considerations: Some children disliked wearing the suits and experienced adverse events including respiratory compromise, overheating and peripheral cyanosis. The suits also impeded function such as independent toileting and dressing. We have downgraded the recommendations based on these side effects to weak negative. Status update since 2013: Newly listed indication.	Low	Weak -	Yellow
	228 Kinematics [BS]	Almeida 2017[158]	CP mixed types Mostly diplegic GMFCS I-III 3-17 yrs	3-18 wks; 30 mins – 12 hrs/day	Includes the same RCTs as Wells 2018, with the addition of 1 small RCT by E-lKafy and El-Shemy. In the El-Kafy trial there was a between group difference favouring the suit. Status update since 2013: Newly listed indication.	Low	Weak -	Yellow
116 **Taping**	229 Improved gross motor function [BS & A]	Unger 2018[159]	CP GMFCS I-V <18 yrs	4-12 wks	5 RCTs. Adjunctive taping combined with NDT conferred better gross motor skills than NDT alone but with varying results between trials. The protocols lack standardisation. Found to be most beneficial with GMFCS I-II, ie children with better selective motor control. (-1 Risk of Bias; - 1 Imprecision). Evidence to Decision Considerations: Taping should be considered an adjunct to therapy, not stand-alone intervention. A small number of children will have a skin allergy to the tape, and allergy is a contraindication. Status update since 2013: Newly listed intervention.	Low	Weak +	Yellow

		Cunha 2018[160]	–	–	Superseded by Unger 2018			
		Fonseca 2018[161]	–	–	Superseded by Unger 2018			
		Guchan 2016[162]	–	–	Superseded by Unger 2018			
		Shamsoddini 2016[163]	–	–	Superseded by Unger 2018			
	230 Improved upper limb function [BS & A]	De Sousa 2017[164]	CP<br>GMFCS I-V<br>3-17 yrs	1-4 days/wk, 3 days – 10 mo duration	6 RCT's, 5 observational studies. Adjunctive taping combined with upper limb motor training conferred better upper limb skills than training alone. (-1 Indirectness; -1 Imprecision). Evidence to Decision Considerations: Taping should be considered an adjunct to motor training, not stand-alone intervention. Children had more active movement when the tape was elasticised compared to rigid tape. Status update since 2013: Newly listed intervention.	Low	Weak +	Yellow
		Cunha 2018[160]	–	–	Superseded by De Sousa 2017			
		Fonseca 2018[161]	–	–	Superseded by De Sousa 2017			
		Ortiz-Ramirez 2017[165]	–	–	Superseded by De Sousa 2017			
		Shamsoddini 2016[163]	–	–	Superseded by De Sousa 2017			
**117 Task Specific Training**	231 Improved gross motor [BS & A]	Toovey 2017[98]	CP<br>GMFCS I-III<br>4-18 yrs	Varied	8 RCTs, 5 observational studies. In this analysis we have excluded the 4 studies that used Goal Directed Training and reported them separated under Goal Directed Training. We also excluded 2 RCTs that compared part-task practice to whole task practice, instead focussing on the studies that compared task specific training (not goal-directed) to a non-task-based control therapy. We also excluded 2 RCT evaluating instrumentation and physical activity in mixed typically developing and CP populations, leaving 2 RCTs for analysis. 2 small RCTs (Bleyenheuft, Declerck) conferred improved gross motor skills compared to control non-task-based therapy. More research is needed (-1 Risk of Bias). Status update since 2013: Newly listed intervention.	Moderate	Strong +	Green
**118 Trans-cranical Direct Current Stimulation [tDCS]**	232 Improved gait [BS & A]	Saleem 2019[166]<br><br>Krishnan 2015[167]	CP hemiplegia & other types<br>4-19 yrs	0.7-1.0 mA	8 RCTs plus other observational studies, indicated tDCS + a motor learning rehabilitation (treadmill or VR) appeared to confer improved gait velocity, stride length and cadence compared to sham tDCS + rehabilitation. Safe and well tolerated by children. Adverse effects are rare, mild, and transient and include minor tingling, burning, itching, and skin redness. These same findings were reported in Krishnan 2015 a systematic review on safety. (-1 Indirectness). Evidence to Decision Considerations: Although the treatment appears safe and well tolerated, the quality of evidence is not yet high enough to upgrade recommendations beyond weak positive. Status update since 2013: Newly listed intervention.	Moderate	Weak +	Yellow
	233 Improved mobility [A]	Hamilton 2018[168]	CP hemiplegia & other types<br>4-19 yrs	0.7-1.0 mA	3 RCTs. No between group differences for tDCS + a motor learning rehabilitation v sham tDCS + rehabilitation on mobility for improved mobility (-1 Risk of Bias; -1 Imprecision). Status update since 2013: Newly listed intervention.	Low	Weak +	Yellow
	234 Improved balance [BS]	Hamilton 2018[168]	CP hemiplegia & other types<br>4-19 yrs	0.7-1.0 mA	4 RCTs. No between group difference for tDCS + a motor learning rehabilitation v sham tDCS + rehabilitation for improving balance (-1 Risk of Bias; -1 Imprecision; -1 Indirectness). Status update since 2013: Newly listed intervention.	Very Low	Weak +	Yellow
	235 Improved	Saleem	CP hemiplegia	0.7-1.0 mA	3 RCTs plus 1 observational study, indicated tDCS + a motor learning rehabilitation	Low	Weak +	Yellow

	hand function [BS & A]	2019[166]	4-19 yrs		(CIMT or functional training) appeared to confer improved quality of movement but not grip strength compared to sham tDCS + rehabilitation (-1 Risk of Bias; -1 Indirectness). Status update since 2013: Newly listed intervention.			
		Plasschaert 2019[101]	–	–	Superseded by Salem 2019			
	236 Reduced dystonia [BS]	Hamilton 2018[168]	CP dystonic 7-19 yrs	1.0 mA cathodal tDCS	1 observation study showed cathodal tDCS reduced dystonia. Status update since 2013: Newly listed intervention.	Very Low	Weak +	Yellow
	237 Reduced spasticity [BS]	Hamilton 2018[168]	CP spastic 9-17 yrs	1.0 mA anodal tDCS	1 RCT showed cathodal tDCS reduced spasticity and improved ROM short term. Status update since 2013: Newly listed intervention	Very Low	Weak +	Yellow
**119 Treadmill Training**	238 Improved walking speed [BS]	Booth 2018[123]	CP GMFCS I-IV 4-21 yrs	Variable 108-1800 mins	9 RCTs and 3 Controlled Trials. Studies examined treadmill training and partial body weight support treadmill training (PBWSTT). Treadmill training conferred improved walking speed in 7 RCTs, whereas 1 RCT found no difference between groups. (-1 Indirectness). Status update since 2013: Newly listed indication.	Moderate	Strong +	Green
		Moreau 2016[127]	CP 4-21 yrs	Variable 8-36 sessions	Meta-analysis of 7 RCTs and 1 Controlled Trial. Examined all types of gait training. Treadmill training conferred improved walking speed in 6 RCTs with high effect sizes (0.92). (-1 Indirectness).			
		Das 2019[65]	–	–	Superseded by Moreau 2016			
	239 Improved walking & endurance [BS]	Booth 2018[123]	CP 4-21 yrs	Variable 8-36 sessions	Meta-analysis of 7 RCTs and 1 Controlled Trial. Examined all types of gait training. Treadmill training conferred improved walking endurance. (-1 Indirectness). Status update since 2013: Quality upgraded from low to moderate. Recommendations for use upgraded from weak positive to strong positive.	Moderate	Strong +	Green
	240 Improved gross motor [BS & A]	Booth 2018[123]	CP 4-21 yrs	Variable 8-36 sessions	Meta-analysis of 7 RCTs and 1 Controlled Trial. Examined all types of gait training. Treadmill training conferred improved gross motor function in 4/5 RCTs. (-1 Indirectness). Status update since 2013: Newly listed indication.	Moderate	Strong +	Green
	241 Improved weight bearing [BS]	–	–	–	Status update since 2013: No new primary source data, no change in quality or recommendations for use gradings.	Low	Weak +	Yellow
**120 Treadmill Training Partial Body Weight Support**	242 Improved walking speed [BS]	Booth 2018[123]	CP GMFCS I-IV 4-21 yrs	Variable 108-1800 mins	9 RCTs and 3 Controlled Trials. Studies examined treadmill training and PBWSTT. Partial body weight support treadmill training conferred improved walking speed in 7 RCTs, whereas 1 RCT found no difference between groups. (-1 Indirectness). Status update since 2013: Newly listed intervention.	Moderate	Strong +	Green
		Moreau 2016[127]	CP 4-21 yrs	Variable 8-36 sessions	Meta-analysis of 7 RCTs and 1 Controlled Trial. Examined all types of gait training. Partial body weight support treadmill training conferred improved walking speed in 6 RCTs) with high effect sizes (0.92). (-1 Indirectness). Status update since 2013: Newly listed intervention.			
		Lefmann 2017[169]	–	–	Superseded by Moreau 2016			
	243 Improved walking endurance [BS]	Booth 2018[123]	CP 4-21 yrs	Variable 8-36 sessions	Meta-analysis of 7 RCTs and 1 Controlled Trial. Examined all types of gait training. Partial body weight support treadmill training conferred improved walking (-1 Risk of Bias; -1 Indirectness). Status update since 2013: Newly listed intervention.	Low	Weak +	Yellow
		Lefmann 2017[169]	–	–	Superseded by Moreau 2016			
	244 Improved gross motor [BS & A]	Moreau 2016[127]	CP 4-21 yrs	Variable 8-36 sessions	Pooled effect of 3 RCTs of PBWSTT on gross motor function was non-significant (-1 Inconsistency; -1 Indirectness). Status update since 2013: Newly listed intervention.	Low	Weak +	Yellow
**121 Vestibular**	245 Improved	Tramontano	CP	30 mins x 10	1 small RCT. No between group differences for vestibular stimulation training + NDT v	Low	Weak -	Yellow

Stimulation Training	gross motor [BS & A]	2017[170]		sessions for 5 wks	NDT alone, both groups showed improved gross motor function. (-2 Risk of Bias: small sample and cross over design). Status update since 2013: Newly listed intervention.			
	246 Improved trunk stabilisation in upright [BS]				1 small RCT. Vestibular stimulation training + NDT conferred better trunk stabilisation in sitting and standing than NDT alone, measured on the Goal Attainment Scale. (-2 Risk of Bias: small sample and cross over design). Status update since 2013: Newly listed intervention.	Low	Weak +	Yellow
122 Vision Training	247 Improved vision processing [BS]	Chorna 2017[12]	CP with CVI<br>7 mo – 4 yrs	2 x 4 wk cycles of training	1 observational study. Vision training using black-and-white slide exposures, black light training and multisensory stimulation conferred improved vision. Preliminary evidence suggested that children with milder physical disability were better treatment responders. (-1 Indirectness). Evidence to Decision Considerations: Benefits outweigh harms and vision is a very important problem to treat. Status update since 2013: Newly listed intervention.	Very Low	Weak +	Yellow
123 Weight Bearing	248 Improved bone mineral density via active weight bearing [BS]	Kim 2017[171]	CP ambulant GMFCS I-II<br>6-12 yrs	40 mins per day x 12 wks	1 RCT. Active weight bearing via a home-based virtual cycling training with a sit to stand component conferred better bone mineral density in the femur than usual physical activity, with moderate effect sizes (Cohen's d = 0.7). (1 Imprecision). Evidence to Decision Considerations: No harms reported, reduction in fractures hypothesised but not tested, low resource use. Status update since 2013: Newly listed indication.	Moderate	Strong +	Green
	249 Improved bone mineral density via a passive standing frame [BS]	Kim 2017[171]	CP<br>GMFCS III-V<br>4-6 yrs	30 mins per day, 5 days per wk 2- 8 mo	2 observational studies. Passive weight bearing in a standing frame conferred may increase bone mineral density in femur. (-1 Risk of Bias; -1 Imprecision). Evidence to Decision Considerations: No harms reported, reduction in fractures hypothesised but not tested, equipment costs, and alternative pharmacological options for bone density exist. Status update since 2013: No change in quality or recommendations for use gradings.	Low	Weak +	Yellow
124 Whole Body Vibration	250 Reduced spasticity [BS]	Ritzmann 2018[97]	CP<br>1-12.3 yrs	sWVB, minimum:5 Hz – maximum: 25 Hz; 0 – 9 mm	1 RCT and 7 observational studies. Whole body vibration may confer short term reduction in spasticity compared to control stretching. (-1 Risk of Bias; -1 Imprecision; -1 Inconsistency). Evidence to Decision Considerations: Highly effective pharmacological substitutes exist. Whole body vibration therefore should not be a frontline approach for this indication. Status update since 2013: Newly listed indication.	Very Low	Weak +	Yellow
	251 Improved muscle activation [BS]	Ritzmann 2018[97]	CP<br>1-12.3 yrs	sWVB, minimum:5 Hz – maximum: 25 Hz; 0 – 9 mm	3 observational studies. Whole body vibration may confer improved muscle activation of the ankle and knee muscles. (-1 Risk of Bias; -1 Imprecision). Evidence to Decision Considerations: Highly effective substitutes exist. Whole body vibration therefore should not be a frontline approach for this indication. Status update since 2013: Newly listed indication	Very low	Weak +	Yellow
	252 Improved kinaesthesia [BS]	Ritzmann 2018[97]	CP<br>1-12.3 yrs	sWVB, minimum:5 Hz – maximum: 25 Hz; 0 – 9 mm	2 observational studies. Whole body vibration may confer improved kinaesthesia. (-1 Risk of Bias; -1 Imprecision). Status update since 2013: Newly listed indication.	Very low	Weak +	Yellow
	253 Improved muscle strength	Ritzmann 2018[97]	CP<br>1-12.3 yrs	sWVB, minimum:5 Hz –	1 RCT and 2 observational studies. Whole body vibration may confer improved muscle strength in both the upper and lower limb muscles. (-1 Risk of Bias; -1 Imprecision). Status update since 2013: Quality upgraded from very low to moderate.	Very low	Weak +	Yellow

	[BS]			maximum: 25 Hz; 0 – 9 mm	Recommendations for use upgraded from weak negative to weak positive.			
	254 Improved gait [BS]	Ritzmann 2018[97]	CP 1-12.3 yrs	sWVB, minimum:5 Hz – maximum: 25 Hz; 0 – 9 mm	2 observational studies. Whole body vibration may confer improved gait kinematics and speed. (-1 Risk of Bias; -1 Imprecision). Evidence to Decision Considerations: Highly effective substitutes exist. Whole body vibration therefore should not be a frontline approach for this indication. Status update since 2013: Quality remains unchanged. Recommendations for use upgraded from weak negative to weak positive.	Very low	Weak +	Yellow
	255 Improved posture [BS]	Ritzmann 2018[97]	CP 1-12.3 yrs	sWVB, minimum:5 Hz – maximum: 25 Hz; 0 – 9 mm	1 observational study. No improvements in posture observed from whole body vibration. (-1 Risk of Bias; -1 Imprecision). Status update since 2013: Newly listed indication.	Very low	Weak -	Yellow
	256 Improved range of motion [BS]	Ritzmann 2018[97]	CP 1-12.3 yrs	sWVB, minimum:5 Hz – maximum: 25 Hz; 0 – 9 mm	3 observational studies. Whole body vibration may confer improved active and passive range of motion in the knee and ankle joints. (-1 Risk of Bias; -1 Imprecision). Evidence to Decision Considerations: Given the discomfort caused by usual care manual stretching, whole body vibration may be a substitute that is more acceptable to children. Status update since 2013: Newly listed indication.	Very low	Weak +	Yellow
	257 Improved gross motor [BS & A]	Ritzmann 2018[97]	CP 1-12.3 yrs	sWVB, minimum:5 Hz – maximum: 25 Hz; 0 – 9 mm	2 observational studies. Whole body vibration may confer improved gross motor skills. (-1 Risk of Bias; -1 Imprecision). Evidence to Decision Considerations: Highly effective substitutes exist. Whole body vibration therefore should not be a frontline approach for this indication. Status update since 2013: Newly listed indication.	Very low	Weak +	Yellow
		Saquetto et 2015[172]	–	–	Superseded by Ritzmann 2018			
		Duquette 2015[173]	–	–	1 additional RCT that did not change the GRADE quality of evidence rating. Superseded by Ritzmann 2018			
		Sa-Caputo 2016[174]	–	–	Superseded by Ritzmann 2018			
**PHARMOCOLOGICAL INTERVENTIONS FOR MANAGING CEREBRAL PALSY**								
**125 Alcohol**	258 Reduced spasticity via local injections [BS]	–	–	–	Evidence to Decision Considerations: Botulinum toxin exists as an effective substitute. Status update since 2013: No new primary source data, no change in quality or recommendations for use gradings.	N/A	Weak -	Yellow
**126 Anti-convulsants**	259 Improved seizure control [BS]	–	–	–	Status update since 2013: No new primary source data, no change in quality or recommendations for use gradings.	N/A	Strong +	Green
**127 Baclofen Intrathecal [ITB]**	260 Reduced spasticity and/or dystonia	Buizer 2019[175]	CP spastic &/or dystonic GMFCS IV-V 1-18 yrs	25-500 mcg Continuous baclofen Varies & can	1 small RCT plus 32 observational studies. Continuous baclofen conferred small to very large effect sizes for reduction of spasticity, and large to very large effect sizes for reduction of dystonia, compared to control no baclofen (-1 Risk of Bias; -1 Imprecision). Evidence to Decision Considerations: Upgraded due to large effect sizes. Conclusions	Low	Strong +	Green

	[BS]			be titrated	are applicable to GMFCS IV-V, less is known about the effects for GMFCS I-III. Status update since 2013: No change in quality or recommendations for use gradings.			
		Hasnat 2015[176]	–	–	Superseded by Buizer			
	261 Reduced dystonia [BS]	Fehlings 2018[177]	CP dystonic GMFCS IV-V 2-42 yrs	Slowly increased to 50-200 µg/day	4 observational studies. 3 studies found baclofen conferred reduced dystonia, 1 study had conflicting findings. (-2 Risk of Bias; -1 Imprecision). Evidence to Decision Considerations: One of the studies was only a single dose infusion which affects the interpretation and certainty of effects for long-term management. Status update since 2013: No change in quality or recommendations for use gradings.	Very low	Weak +	Yellow
	262 Improved gross motor skills [BS & A]	Buizer 2019[175]	CP spastic &/or dystonic GMFCS IV-V 1-18 yrs	Not reported	1 small RCT plus other lower level evidence. Small effect sizes for participation and reduction of caregiver burden (-1 Risk of Bias; -1 Imprecision). Evidence to Decision Considerations: Conclusions are applicable to GMFCS IV-V, less is known about the effects for GMFCS I-III where it might be theoretical possible to achieve larger clinical gains for this indication. Status update since 2013: Newly listed indication.	Low	Weak +	Yellow
		Hasnat 2015[176]	–	–	Superseded by Buizer.			
	263 Improved participation & health related quality of life [P]	Buizer 2019[175]	CP spastic &/or dystonic GMFCS IV-V 1-18 yrs	Not reported	1 small RCT plus observational evidence. Small effect sizes for improved gross motor (-1 Risk of Bias; -1 Imprecision). Status update since 2013: No change in quality or recommendations for use gradings.	Low	Weak +	Yellow
		Tsoi 2012[178]	–	–	Superseded by Buizer 2019			
	264 Improved walking [A]	–	–	–	Status update since 2013: No new primary source data, no change in quality or recommendations for use gradings.	Very low	Weak +	Yellow
	265 Reduced pain [BS]	Ostojic 2018[179]	CP spastic &/or dystonic GMFCS IV-V 1-16 yrs	65–199 µg/day	2 RCTs plus observational studies. Pain caused by hypotonia is reduced from ITB (-1 Risk of Bias; -1 Imprecision). Evidence to Decision Considerations: Conclusions are applicable to GMFCS IV-V. Acceptability is enhanced by continuous baclofen has the dual benefit of spasticity/dystonia reduction and pain reduction. Status update since 2013: Newly listed indication.	Low	Weak +	Yellow
		Beecham 2015[180]	–	–	Superseded by Ostojic 2018			
	266 Prevent hip displace-ment [BS]	Miller 2017[147]	CP GMFCS I-V	Varied	1 observational study. ITB may reduce hip displacement by a very small amount. (-1 Risk of Bias). Evidence to Decision Considerations: Management of hip displacement requires a comprehensive approach. Longitudinal population-based studies in Sweden have demonstrated that the most effective method for preventing hip displacement is regular hip surveillance, with comprehensive multidisciplinary management, at the right time. Status update since 2013: Newly listed indication.	Low	Weak +	Yellow
**128 Baclofen Oral**	267 Reduced spasticity and/or dystonia [BS]	Navarrete-Opazo 2016[181]	CP spastic 2-16 yrs	Slowly increased to 2-40 mg/day <8yo; 40-60 mg/day >8 yrs	6 small RCTs, 5/6 used the crossover methodology. Oral baclofen reduces spasticity and dystonia (-1 Risk of Bias; -1 Indirectness; -1 Imprecision). Evidence to Decision Considerations: Oral baclofen requires high doses to induce an effect, which elevates the risks for side effects including seizures and drowsiness. Status update since 2013: No change in quality or recommendations for use gradings.	Low	Weak +	Yellow
		Ward 2017[182]	CP 0-2 yrs	Varied	1 retrospective audit about baclofen, inside a systematic review of multiple different tone management strategies for infants with CP (-2 Risk of Bias; -1 Indirectness; -1 Imprecision). Evidence to Decision Considerations: Effects are essentially unknown, but the side effects of drowsiness and induction of seizures need to be carefully weighted up	Very low	Weak +	Yellow

					in this population. Substitutes are often considered such as benzodiazepines which offer the dual action of seizure and spasticity management. Status update since 2013: Newly listed indication in this age group.			
129 **Bis-phosphonates**	268 Improved bone mineral density [BS]	Kim 2015[183]	CP	Varied	1 RCT, 3 observational studies. Bisphosphonates conferred improved Z-score of lumbar spine compared to pre-treatment values [SMD 0.79, 95%CI 0.49–1.10], and conferred improved Z-score of femur compared to pre-treatment values [SMD 0.75, 95%CI 0.38–1.11]. (-1 Risk of Bias). Evidence to Decision Considerations: Benefits outweigh harms. Harms from not acting on fracture risk. Status update since 2013: No change in quality or recommendations for use gradings.	Moderate	Strong +	Green
		Ozel 2016[184]	–	–	Superseded by Kim 2015.			
130 **Botulinum Toxin (BoNT)**	269 Reduced lower limb spasticity [BS]	Kahraman 2016[185]	CP<br>2-18 yrs	Varies.<br>Botox 4-6 U/kg	Repeat dosing appears safe and efficacious. Evidence to Decision Considerations: Experts have called for more research into whether or not there is long-term harm from muscle atrophy and loss of contractile elements following Botulinum toxin. What is not known is whether the atrophy and insertion of replacement fat and connective tissue process that ensures is a direct or accelerated adverse event from BoNT or whether this is the natural history of CP. Status update since 2013: No additional systematic reviews on efficacy therefore no change in quality or recommendations for use gradings.<br><br>There is strong evidence that instrumented guidance of BoNT is more accurate.	High	Strong +	Green
	270 Reduced upper limb spasticity [BS]	Kahraman 2016[185]	CP<br>2-18 yrs	Varies.<br>Botox 4-6 U/kg	Repeat dosing appears safe and efficacious. Status update since 2013: No additional systematic reviews on efficacy therefore no change in quality or recommendations for use gradings.	Moderate	Strong +	Green
	271 Reduced spasticity and increased ROM [BS]	Druschel 2013[186]	CP<br>0-2 yrs	6 U/kg body weight diluted to 50 U/mL	Only 1 small RCT (Tedroff) in children <2 yrs. (-1 Risk of Bias; -1 Imprecision). Evidence to Decision Considerations: Less is known about the efficacy of BoNT in the <2 yr age group than >2 yr age group, due to usual care involving delaying the provision of injections to prevent infant botulism. Status update since 2013: Newly listed indication in this age group.	Low	Weak +	Yellow
	272 Reduced neck dystonia [BS]	–	–	–	Status update since 2013: No new primary source data, no change in quality or recommendations for use gradings.	Very low	Weak +	Yellow
	273 Reduced pain [BS]	Pin 2013[187]	CP<br>GMFCS IV-V	Botox 4-6 U/kg or not reported	One small RCT. BoNT conferred improvements in post-operative pain. (Risk of Bias -1, Imprecision -1.) Evidence to Decision Considerations: The dual action of spasticity reduction and pain reduction makes BoNT an acceptable intervention. Status update since 2013: Quality upgraded from very low to low, no change in recommendations for use.	Low	Weak +	Yellow
	274 Reduced sialorrhea [BS]	Sridharan 2018[188]	CP<br>2-18 yrs	Varies	15 RCTs of varying treatments in varying populations. Pooled analysis for CP only indicated that botulinum toxins A and B conferred reduction in the frequency and severity of sialorrhea, A [MD -1.9, 95%CI -3.03 to 0.66], B [MD -16.67, 95%CI -24.06 to -9.28]. Evidence to Decision Considerations: Consider the child's total management plan (including upper and lower limb injections) to ensure enough dose is available within safe dosing limits. Status update since 2013: No change in quality or recommendations for use grading.	Moderate	Strong +	Green
		Rodwell 2012[189]	–	–	Superseded by Sridharan 2018.			
	275 Prevent hip	Miller	CP	Varied	4 observational studies. BoNT may reduce hip displacement in some children. (-1 Risk of	Low	Weak +	Yellow

	displacement [BS]	2017[147]	GMFCS I-V		Bias). Evidence to Decision Considerations: Management of hip displacement requires a comprehensive approach. Longitudinal population-based studies in Sweden have demonstrated that the most effective method for preventing hip displacement is regular hip surveillance, with comprehensive multidisciplinary management, at the right time.			
**131 BoNT + Occupational Therapy**	276 Improved goal achievement [A]	Mathevon 2018[190]	CP spastic 2-18 yrs	Varies. Botox 4-6 U/kg	Status update since 2013: No new primary source data, no change in quality or recommendations for use gradings.	High	Strong +	Green
**132 BoNT + Physiotherapy**	277 Improved gait [BS]	Fonseca 2018[161]	CP spastic	Botox 4-6 U/kg or not reported	4 small RCTs. 1 RCT (Physiotherapy = goal oriented functional training + home program + BoNT) conferred improved gait on the COPM measure. 3 RCTs showed no statistically significant between treatment groups for gait (PT involved stretching and/or goal oriented functional training). (-1 Risk of Bias; -1 Inconsistency; -1 Imprecision). Evidence to Decision Considerations: BoNT + physiotherapy is the standard of care and conventional wisdom, this affects the number of trials conducted. Status update since 2013: Newly listed indication.	Very Low	Weak +	Yellow
	278 Improved gross motor function [BS & A]	Yana 2019[191]	CP spastic GMFCS I-III 2-18 yrs	Varies. 3-30 U/Kg	4 RCTs. 3 studies indicated Botox + PT conferred better gross motor function than PT alone, whereas 2 showed no difference between groups. One study used very high doses and varied the injection technique between groups introducing confounders and therefore must be interpreted cautiously. (-1 Risk of Bias; -1 Inconsistency; -1 Imprecision). Status update since 2013: Newly listed indication.	Very low	Weak +	Yellow
	279 Reduced spasticity [BS]	Yana 2019[191]	CP spastic GMFCS I-III 2-18 yrs	Varies. 3-30 U/Kg	3 RCTs. 2 studies indicated BoNT + PT conferred better reduction in spasticity, whereas 1 study showed no difference between groups. (-1 Risk of Bias; -1 Inconsistency; -1 Imprecision). Status update since 2013: Newly listed indication.	Very low	Weak +	Yellow
**133 BoNT + Resistance Training**	280 Reduced spasticity & improved muscle strength and volume [BS]	Mathevon 2018[190]	CP spastic 2-18 yrs	BoNT: unknown Resistance: 2-3 x wk for 8-12 wks	3 small RCTs: 1 RCT found BoNT + resistance training conferred improved spasticity, strength and muscle volume compared to controls. 1 RCT conferred increased strength in both groups and 1 RCT conferred increased plantarflexions median torque compared to controls but otherwise no difference between groups. (-1 Risk of Bias; -1 Inconsistency; -1 Imprecision). Status update since 2013: Newly listed indication.	Very low	Weak +	Yellow
**134 BoNT + Electrical Stimulation**	281 Improved upper limb function [A]	Mathevon 2018[190]	CP spastic 2-18 yrs	2x daily 15 mins post BoNT	1 RCT. 1 RCT found BoNT + electrical stimulation conferred function, but not difference between groups for spasticity and range of motion compared to control BoNT alone. (-1 Risk of Bias; -1 Imprecision). Status update since 2013: Newly listed indication.	Low	Weak +	Yellow
	282 Improved gait speed [BS]	Mathevon 2018[190]	CP spastic 2-18 yrs	Daily for 4 wks	2 RCTs and 1 observational study. No between group differences for gait speed for BoNT + electrical stimulation compared to BoNT alone. (-1 Risk of Bias; -1 Imprecision). Status update since 2013: Newly listed indication.	Low	Weak +	Yellow
**135 BoNT + Serial Casting**	283 Improved range of motion [BS]	Mathevon 2018[190]	CP spastic 2-18 yrs	3 progressive casts, 0-4 wks post BoNT	6 RCTs. BoNT + casting conferred better improvements in range of motion compared to casting alone. Evidence to Decision Considerations: Muscle atrophy and pain was higher from BoNT + casting. Discomfort was higher when the casts were applied immediately post BoNT than when they were applied 4 wks post BoNT. Status update since 2013: Newly listed indication.	High	Strong +	Green
**136 BoNT + Night Hand Splints**	284 Improved upper limb function [A]	Mathevon 2018[197]	CP spastic 2-18 yrs	Nightly	1 RCT. 1 RCT found BoNT + a night hand splint conferred better hand function than BoNT alone. (-1 Risk of Bias; -1 Imprecision). Status update since 2013: Newly listed indication.	Low	Weak +	Yellow
**137 BoNT + Hip Bracing**	285 Prevent hip displacement [BS]	Miller 2017[145]	CP GMFCS II-V	Varied	1 RCT. BoNT + SWASH brace may reduce hip displacement in some children by a very small amount. Authors did not conclude the change was clinically meaningful. (-1 Risk of Bias; -1 Imprecision). Evidence to Decision Considerations: Management of hip displacement requires a comprehensive approach. Longitudinal population-based studies in Sweden have demonstrated that the most effective method for preventing hip	Low	Weak +	Yellow

					displacement is regular hip surveillance, with comprehensive multidisciplinary management, at the right time. Status update since 2013: Newly listed indication.			
**138 Benztropine**	286 Reduced sialorrhea [BS]	Sridharan 2018[188]	CP<br>2-18 yrs	Varies	15 RCTs of varying treatments in varying populations. Pooled analysis for CP only indicated that benztropine conferred reduction in the frequency and severity of sialorrhea [MD -6.04, 95%CI -8.20 to -3.88]. (-1 Risk of Bias; -1 Imprecision). Status update since 2013: Newly listed intervention.	Low	Weak +	Yellow
		Dias 2016[192]	–	–	Superseded by Sridharan 2018			
**139 Dantrolene**	287 Reduced spasticity [BS]	–	–	–	Status update since 2013: No new primary source data, no change in quality or recommendations for use gradings.	Low	Weak -	Yellow
**140 Diazepam**	288 Reduced spasticity [BS]	–	–	–	Status update since 2013: No new primary source data, no change in quality or recommendations for use gradings.	Moderate	Strong +	Green
**141 Gabapentin**	289 Reduced pain [BS]	Ostojic 2018[179]	CP Spastic or Dystonic<br>GMFCS V<br>1-18 yrs	18.1 mg/kg with dystonia<br>7.61 mg/kg no dystonia	1 observational study by Liow 2015, indicating gabapentin compared to placebo had no benefit, but gabapentin combined with ITB compared to control reduced pain. (-2 Risk of Bias; -1 Imprecision). Status update since 2013: Newly listed intervention.	Very low	Weak +	Yellow
	290 Reduced dystonia [BS]	Liow 2015[193]	CP Spastic or Dystonic<br>GMFCS V<br>1-18 yrs	18.1 mg/kg with dystonia<br>7.61 mg/kg no dystonia	1 observational study indicated reduced dystonia (-2 Risk of Bias; -1 Indirectness; -1 Imprecision). Evidence to Decision Considerations: In situations with combined dystonia and epilepsy, Gabapentin may benefit both. However, the quality of evidence is not high enough to upgrade recommendations. Status update since 2013: Newly listed intervention.	Very low	Weak +	Yellow
	291 Improved activities and participation [A & P]	Liow 2015[193]	CP Spastic or Dystonic<br>GMFCS V<br>1-18 yrs	18.1 mg/kg with dystonia<br>7.61 mg/kg no dystonia	1 observational study indicated reduced dystonia (-2 Risk of Bias; -1 Indirectness; -1 Imprecision). Status update since 2013: Newly listed intervention.	Very low	Weak +	Yellow
**142 Glycopyrrolate**	292 Reduced sialorrhea [BS]	Sridharan 2018[188]	CP<br>2-18 yrs	Varies	15 RCTs of varying treatments in varying populations. Pooled analysis for CP only indicated that glycopyrrolate conferred a small reduction in the frequency and severity of sialorrhea [MD -0.72, 95%CI -1.72 to -0.17]. (-1 Risk of Bias; -1 Imprecision). Status update since 2013: Newly listed intervention. Evidence to Decision Considerations: Substitute sialorrhea interventions with larger effect sizes exist. Status update since 2013: Newly listed intervention.	Low	Weak +	Yellow
		Dias 2016[192]	–	–	Superseded by Sridharan 2018			
		Eiland 2012[194]	–	–	Superseded by Sridharan 2018			
**143 Levodopa**	293 Improved upper limb function [A]	Fehlings 2018[177]	CP Dystonic<br>GMFCS II-V<br>9-27 yrs	150 mg/day<br>1<5 kg	1 RCT by Pozin, showed no improvement in upper limb function (-1 Risk of Bias; -1 Indirectness). Status update since 2013: Newly listed intervention.	Low	Weak -	Yellow
**144 Melatonin**	294 Improved sleep [BS]	Galland 2012[29]	CP mixed types<br>9 mo – 18 yrs	Daily	No CP specific trials, data comes from data in neuro-disability. Melatonin confers improved sleep time and parent perceptions of their child's sleep quality, but does not appear to improve sleep onset. (-1 Risk of Bias; -1 Indirectness). Status update since 2013: Newly listed intervention.	Very low	Weak +	Yellow
**145 Phenol**	295 Reduced spasticity	Gonnade 2017[195]	CP diplegia<br>GMFCS I-III	Phenol: 6%<br>BoNT: 1-3	1 RCT, comparing phenol head-to-head with BoNT. BoNT conferred better reduction in spasticity reduction than phenol. The result were also longer lasting in BoNT (24 wks)	Moderate	Weak -	Yellow

	[BS]		4-10 yrs	U/Kg <6 U/kg	than phenol (6 wks). (-1 Risk of Bias). Evidence to Decision Considerations: Phenol had a higher side effects profile, including skin irritation, permanent peripheral nerve palsy, and painful muscle necrosis. Status update since 2013: Quality is upgraded from low to moderate. Recommendations for use remain unchanged.			
**146 Scopolamine**	296 Reduced sialorrhea [BS]	Dias 2016[192]	CP 12-58 yrs	–	Non-systematic review. Included 1 RCT for Scopolamine. Scopolamine conferred reduction in the frequency and severity of sialorrhea compared to controls. (-1 Risk of Bias; -1 Imprecision). Evidence to Decision Considerations: Side effects observed in 23%. Status update since 2013: Newly listed intervention.	Low	Weak +	Yellow
**147 Tizanidine**	297 Reduced spasticity [BS]	–	–	–	Status update since 2013: No new primary source data, no change in quality or recommendations for use gradings.	Low	Weak +	Yellow
**148 Tri-hexyphenidyl [Artane]**	298 Reduced dystonic or athetoid movement [BS]	Masson 2017[196]	CP Athetoid and Dystonic (GMFCS III-V) 2-17 yrs	Start dose: 0.1-5 mg Final dose: 7.5-10 mg	3 small RCTs, and 1 observational study. No difference between groups for trihexyphenidyl v control for improving athetosis or dystonia symptoms. Trihexyphenidyl often associated with side effects (-1 Risk of Bias; -1 Imprecision). Status update since 2013: Newly listed intervention.	Low	Weak -	Yellow
		Fehlings 2018[177]	–	–	Identical source articles Masson 2017.			
		Harvey 2018[197]	–	–	Superseded by Masson 2017.			
	299 Improved quality of upper limb movement [BS]	Masson 2017[196]	CP athetoid and dystonic 2-17 yrs	Start dose: 0.1-5 mg Final dose: 7.5-10 mg	2 small RCTs with conflicting findings. 1 RCT reported trihexyphenidyl conferred upper limb quality of movement gains after 15 wks treatment better than control. 1 RCT found no difference between groups for quality of upper limb movement measured on the QUEST, however the authors used QUEST total scores rather than the Rasch recommended domains A and B only (-1 Risk of Bias; -1 Inconsistency; -1 Indirectness; -1 Imprecision). Status update since 2013: Newly listed intervention	Very low	Weak -	Yellow
		Harvey 2018[197]	–	–	Superseded by Masson 2017.			
	300 Improved participation [P]	Harvey 2018[197]	CP dystonia 2-17 yrs	Start dose: 0.2 mg 3x dose Final dose: 2.5 mg 3x dose	1 small RCT. Trihexyphenidyl conferred better goal achievement in participation on the GAS and COPM than control no trihexyphenidyl (-1 Risk of Bias; -1 Imprecision). Evidence to Decision Considerations: may also help sialorrhea but use should be balanced against potential constipation and behavioural side effects. The recommendations are unchanged by this. Status update since 2013: Newly listed intervention.	Low	Weak +	Yellow
	301 Reduced sialorrhea [BS]	Dias 2016[192]	CP dystonia	0.095-0.55mm/kg/day	Non-systematic review, included 1 observational study for Trihexyphenidyl. Trihexyphenidyl conferred reduction in the frequency and severity of drooling. (-1 Risk of Bias; -1 Imprecision). Evidence to Decision Considerations: Side effects observed in 69%. Substitute sialorrhea interventions exist with lower rates of side effects. Status update since 2013: Newly listed intervention.	Very low	Weak -	Yellow
**149 Vitamin D [& calcium]**	302 Improved bone mineral density [BS]	Ozel 2016[184]	CP GMFCS III-V	800-1000 IU	2 observational studies. Vitamin D appeared to confer improved bone mineral density. Evidence to Decision Considerations: Bone density is an important clinical problem to prevent or treat. Status update since 2013: No change in quality or recommendations for use gradings.	Low	Weak +	Yellow

SURGICAL INTERVENTIONS FOR MANAGING CEREBRAL PALSY

**150 Deep Brain Stimulation [DBS]**	303 Reduced dystonia [BS]	Fehlings 2018[177]	CP dystonic 3-57yrs	Varied	12 observational studies. 4 studies indicated DBS reduced dystonia, 4 studies indicated no change in dystonia (-1 Risk of Bias; -1 Imprecision). Evidence to Decision Considerations: A dystonic storm can be life-threatening painful, and in these	Very low	Weak +	Yellow

					circumstances the benefits outweigh the risks. DBS might be best applied within embedded research studies in clinical care to ensure adequate monitoring and learning about this rare indication. Status update since 2013: Newly listed intervention.			
		Elia 2018[198]	–	–	Superseded by Fehlings 2018.			
	304 Improved motor function [BS & A]	Fehlings 2018[177]	CP dystonic 3-57 yrs	Varied	8 observational studies. 4 studies indicated improved motor function, 4 studies indicated no change in motor function (-1 Risk of Bias; -1 Imprecision). Status update since 2013: Newly listed intervention.	Very low	Weak +	Yellow
	305 Reduced pain [BS]	Fehlings 2018[177]	CP dystonic 3-57 yrs	Varied	4 observational studies. 2 studies indicated DBS conferred reduced pain, 2 studies indicated no change in pain (-1 Risk of Bias; -1 Imprecision). Status update since 2013: Newly listed intervention.	Very low	Weak +	Yellow
	306 Reduced caregiver burden	Fehlings 2018[177]	CP dystonic 3-57 yrs	Varied	1 observational study indicated reduced caregiver burden (-1 Risk of Bias; -1 Imprecision). Status update since 2013: Newly listed intervention.	Very low	Weak +	Yellow
**151 Dental Care**	307 Improved dental health [BS]	Dieguez-Perez 2016[199]	CP mixed types	Varied	11 observational studies. Children with CP have higher rates of dental caries, dental trauma, poor oral hygiene and poor gingival health than typically developing children. Early and regular routine dental confers improved dental health. Evidence to Decision Considerations: Harms rise from not providing dental care. Furthermore, dental problems cause pain, which is a known trigger for behavioural problems. We therefore upgraded the recommendation for use. Status update since 2013: Newly listed intervention.	Low	Strong +	Green
**152 Equinus Deformity Correction**	308 Reduced foot deformity [BS]	–	–	–	Status update since 2013: No new primary source data for treating equinus in isolation, no change in quality or recommendations for use gradings. Refer to Single Event Multi-Level Surgery.	Low	Weak +	Yellow
**153 Femoral Derotation Osteotomy**	309 Reduced pelvic retraction and hip internal rotation [BS]	Carty 2014[200]	CP unilateral 6-12 yrs	Femoral Derotation Osteotomy	13 observational studies (5 prospective, 8 retrospective). Femoral derotation osteotomy conferred reduced pelvic retraction by 9 degrees and hip internal rotation by 17 degrees (-1 Risk of Bias). Evidence to Decision Considerations: Magnitude of rotational correction in surgery was greater than observed kinematic changes for unilateral and bilateral CP. Surgery before 10 yrs of age was associated with a greater recurrence rate of internal hip rotation. Status update since 2013: Newly listed intervention.	Very low	Weak +	Yellow
	310 Improved pelvic and hip rotation [BS]	Carty 2014[200]	CP bilateral 6-12 yrs	Femoral Derotation Osteotomy	13 observational studies (5 prospective, 8 retrospective). Femoral derotation osteotomy conferred reduced hip internal rotation by 14 degrees, but there were no improvements in pelvic retraction (-1 Risk of Bias). Evidence to Decision Considerations: Magnitude of rotational correction in surgery was greater than observed kinematic changes for unilateral and bilateral CP. Surgery before 10 yrs of age was associated with a greater recurrence rate of internal hip rotation. Status update since 2013: Newly listed intervention.	Very low	Weak +	Yellow
**154 Fundo-plication**	311 Reduced gastro oesophageal reflux [BS]	–	–	–	Status update since 2013: No new primary source data, no change in quality or recommendations for use gradings.	N/A	Weak +	Yellow
	312 Improved rate of gastric emptying [BS]	Brun 2013[201]	CP <18 yrs	2 meals on separate days	1 trial. No differences between the groups for rates of gastric emptying after the 2 meals in children with a fundoplication v children without a fundoplication. (-2 Risk of Bias). Status update since 2013: Newly listed indication.	Low	Weak -	Yellow
	313 Reduced postprandial	Brun 2013[201]	CP <18 yrs	2 meals on separate	1 trial. No differences between the groups for postprandial symptoms in children with a fundoplication v children without a fundoplication. (-2 Risk of Bias). Status update since	Low	Weak -	Yellow

	symptoms [BS]			days	2013: Newly listed indication.			
**155 Gastrostomy**	314 Improved safety after PEG insertion [BS]	Islek 2013[202]	Neurological conditions including CP 1-9 yrs	50% of the volume for standard feed	1 RCT. No between group difference for adverse events in the group fed 4hrs after tube insertion versus 12hrs after tube insertion. (-1 Risk of Bias). Evidence to Decision Considerations: Feed was well tolerated at 4 hrs, conferring earlier discharge from hospital with cost savings. Status update since 2013: Newly listed intervention.	Moderate	Weak +	Yellow
	315 Improved growth [BS]	Ferluga 2014[203]	CP GMFCS I-V	Varied	1 RCT, 10 observational studies. Gastrostomy feeding conferred weight gain, however many remained underweight. No significant improvement in height or aspiration incidence in the studies. (-1 Risk of Bias; -1 Imprecision). Evidence to Decision Considerations: Adverse events included site infection (59%), granulation tissue (42%), and recurrent reflux (30%). Death rates ranged from 7 to 29%; however, the underlying cause was probably not the surgery. Status update since 2013: Upgraded the quality of the evidence from very low to low. Recommendations for use remain unchanged.	Low	Weak +	Yellow
**156 Hand Surgery**	316 Reduced thumb in palm posture [BS]	–	–	–	Status update since 2013: No new primary source data, no change in quality or recommendations for use gradings.	Very low	Weak +	Yellow
	317 Improved tactile function [BS]	Auld 2014[149]	CP type not specified	Single surgery type not specified	2 observational studies with conflicting findings. Authors conclude it is unclear. The validity of the stereognosis measure affects interpretation of findings (-1 Risk of Bias; -1 Inconsistency; -1 Indirectness; -1 Imprecision). Status update since 2013: Newly listed indication.	Very low	Weak +	Yellow
**157 Hip Surveillance**	318 Prevent hip displacement [BS]	Wynter 2015[204]	CP GMFCS I-V	Commence at 12-24 mo review annually	4 observational studies. Hip surveillance conferred statistically significant improvements in slowing the rate of hip displacement or preventing hip displacement, compared with CP controls who did not have hip surveillance. (+1 population-based samples with low risk of bias). Evidence to Decision Considerations: GMFCS level is risk factor for hip displacement, i.e. the more severely the motor impairment the higher the risk of hip displacement. Clinical practice guidelines exist, outlining the exact measurement intervals and the radiographic procedures. Guidelines should be followed. Status update since 2013: No change in quality or recommendations for use gradings.	Moderate	Strong +	Green
		Miller 2017[147]	–	–	1 RCT, 23 observational studies analysing individual intervention's efficacy for preventing hip displacement, which have been reported separately under BoNT, ITB, obturator nerve block, positioning & SDR. Combined effect not examined in this study, therefore superseded by Wynter 2015. Status update since 2013: No change in quality or recommendations for use gradings.			
**158 Obstructive Sleep Apnoea Surgery**	319 Reduced sleep disturbance [BS]	Galland 2012[29]	CP mixed types 9 mo – 18 yrs	Surgical procedures	3 observational studies. Surgery conferred small improvements in sleep. (-1 Indirectness) Evidence to Decision Considerations: 1 in 3 required repeat operations. Status update since 2013: Newly listed intervention.	Low	Weak +	Yellow
**159 Obturator Nerve Block**	320 Prevent hip displacement [BS]	Miller 2017[147]	CP GMFCS III-IV	Varied	1 observational study. Obturator nerve block may reduce hip displacement in some children by a very small amount. (-1 Risk of Bias). Evidence to Decision Considerations: Management of hip displacement requires a comprehensive approach. Longitudinal population-based studies in Sweden have demonstrated that the most effective method for preventing hip displacement is regular hip surveillance, with comprehensive multidisciplinary management, at the right time.	Low	Weak +	Yellow
**160 Radio-frequency lesion of the**	321 Reduced pain [BS]	Ostojic 2018[179]	CP Spastic GMFCS V 5-22 yrs	Under GA, radio frequent	1 small observational study. Radio frequency lesion conferred reduction in pain, caregiver burden, and spasticity (-1 Risk of Bias; -1 Indirectness; -1 Imprecision). Status update since 2013: Newly listed intervention.	Very low	Weak +	Yellow

**dorsal root ganglion [RF-DRG]**				current to increase temp to 67°C 60 sec				
**161 Recon-structive Hip Surgery**	322 Reduced hip displace-ment [BS]	El-Sobky 2018[205]	CP<br>GMFCS III-V<br>3-14 yrs	Varus Derotation Osteotomy	36 observational studies (1 prospective, 35 retrospective). 53% had varus derotation osteotomy (VRDO) + pelvic osteotomy, 45% VRDO alone, 1% pelvic osteotomy alone. Almost all also had adductor release +/- iliopsoas release or iliopsoas tenotomy. Pelvifemoral hip reconstruction conferred better hip location than VRDO alone both short-term and long-term. VRDO alone had 37% complication rates and those who were older and GMFCS V were most at risk. (-1 Risk of Bias). Evidence to Decision Considerations: The decision on when to perform a pelvic osteotomy in the setting of hip reconstruction remains elusive. Future investigation with the use of intraoperative arthrography may help standardize this decision process. The greatest success of hip reconstruction was seen in surgeons who are experienced in caring for children with CP. Status update since 2013: No change in quality or recommendations for use gradings.	Very low	Weak +	Yellow
		Hesketh 2016[206]	–	–	Superseded by El-Sobky 2018. 7% avascular necrosis complication rate, with no identifiable risk factors. Status update since 2013: No change in quality or recommendations for use gradings.			
**162 Salvage Hip Surgery**	323 Reduced pain [BS]	Kolman 2016[207]	CP non-ambulant<br>GMFCS IV-V<br><18 yrs	Salvage surgery	21 observational studies. Femoral head resection salvage surgery conferred better pain reduction than arthrodesis [OR 7.3, 95%CI 2.2-24.8]. Valgus osteotomy salvage surgery conferred better pain reduction than arthrodesis [OR 5.9, 95%CI 1.6-22.8]. Total hip arthroplasty salvage surgery conferred better pain reduction than arthrodesis [OR 11.7, 95%CI 1.1-297.5]. (-1 Risk of Bias). Evidence to Decision Considerations: Arthrodesis had a higher complication rate. When considering salvage hip surgical procedures (femoral head resection, valgus osteotomy and hip replacement) for children with CP there is optimal procedure in terms of pain relief and function. However, one should avoid performing a hip arthrodesis in the setting of salvage hip surgery. Status update since 2013: Newly listed intervention.	Very low	Weak +	Yellow
		Ostojic 2018[179]	–	–	Superseded by Kolman 2016			
		Boldingh 2014[208]	–	–	Superseded by Kolman 2016			
	324 Reduced pain [BS]	Kolman 2016[207]	CP ambulant<br><18 yrs	Salvage surgery	8 observational studies. 3 studies found total hip arthroplasty salvage surgery conferred 93% pain reduction, with 14% requiring a revision. (-1 Risk of Bias). 1 study found arthrodesis salvage surgery conferred pain reduction for n=4/4 patients, with no complications. (-1 Risk of Bias). Evidence to Decision Considerations: For ambulant CP patients, total hip arthroplasty has demonstrated excellent pain relief and function, but with a higher rate of complications compared to a general cohort of patients undergoing total hip arthroplasty. Status update since 2013: Newly listed intervention.	Very low	Weak +	Yellow
**163 Selective Dorsal Rhizotomy [SDR]**	325 Reduced spasticity [BS]	Health Quality Ontario[209]	CP spastic	–	1 meta-analysis, 5 RCTs plus 75 observational studies. No new primary source data since our 2013 review which showed SDR + physiotherapy conferred better reduction in lower limb spasticity than control physiotherapy alone (-1 Inconsistency). Status update since 2013: No change in quality or recommendations for use gradings.	Moderate	Strong +	Green
	326 Improved gait kinematics [BS]	–	–	–	Status update since 2013: No new primary source data, no change in quality or recommendations for use gradings.	Low	Strong +	Green
	327 Improved	Health	CP spastic	–	No new primary source data since our 2013 review but a new meta-analysis showed SDR	Low	Weak +	Yellow

	gross motor function [A]	Quality Ontario[209]			+ physiotherapy conferred better gross motor function than control physiotherapy alone (1 Inconsistency; -1 Indirectness). Status update since 2013: Newly listed intervention.			
	328 Improved function & participation [A & P]	–	–	–	Status update since 2013: No new primary source data, no change in quality or recommendations for use gradings.	Very low	Weak +	Yellow
	329 Prevent hip displacement [BS]	Miller 2017[147]	CP GMFCS I-III	–	7 observational studies. Conflicting findings, some studies indicated SDR assisted in stabilising or improving hip displacement, whereas others did not. (-1 Risk of Bias; -Imprecision). Evidence to Decision Considerations: Management of hip displacement requires a comprehensive approach. Longitudinal population-based studies in Sweden have demonstrated that the most effective method for preventing hip displacement is regular hip surveillance, with comprehensive multidisciplinary management, at the right time. Status update since 2013: Newly listed intervention.	Very low	Weak +	Yellow
**164 Scoliosis Surgical Correction**	330 Scoliotic deformity correction [BS]	Toovey 2017[210]	CP GMFCS IV-V <18 yrs	Spinal Fusion	32 observational studies. Spinal fusion conferred 59% improvement in the curve, short term; 61% improvement in the curve, medium term; and 61% improvement in the curve long-term. Mean change in Cobb angles 65-82 degrees pre-operatively to 19-37 degrees post-operatively. (-2 Risk of Bias; +1 Large Effects). Evidence to Decision Considerations: High acceptability to patients despite the 38% complication rate in these medically fragile children. Improved curve angle does not confer improved function or gross motor function but may improve quality of life. Evidence to Decision Considerations: Scoliosis surgery in GMFCS IV/V children is associated with significant correction of spinal deformity; however, this surgery is associated with moderated risk of complication, uncertain change in quality of life and with an unknown trajectory of natural history of untreated progressive scoliosis, decision for surgery must be made on an individual basis. Status update since 2013: Newly listed intervention.	Low	Strong +	Green
		Legg 2014[211]	–	–	Superseded by Toovey 2017			
	331 Pelvic obliquity correction [BS]	Toovey 2017[210]	CP GMFCS IV-V <18 yrs	Spinal Fusion	26 observational studies measured pelvic obliquity, but with varying definitions. Spinal fusion conferred 65% improvement in pelvic obliquity, short term and 55% long-term. Mean change in obliquity angles 9-25 degrees pre-operatively to 4-10 degrees post-operatively. (-1 Risk of Bias; -1 Indirectness; +1 Large Effects). Evidence to Decision Considerations: 38% complication rate, however these children are often medically fragile. Surgical correction of scoliosis may improve pelvic obliquity, but it is unclear how this affects quality of life and caregiver burden. Status update since 2013: Newly listed intervention.	Very low	Weak +	Yellow
	332 Quality of life [BS, A, P, EF, PF]	Toovey 2017[210]	CP GMFCS IV-V <18 yrs	Spinal Fusion	3 observational studies, using different measures. Spinal fusion conferred improved quality of life at 2 yrs post-operatively. (-1 Risk of Bias; -1 Indirectness; -1 Imprecision). Evidence to Decision Considerations: Limited data from 3 observational studies existed at the time of the Toovey meta-analysis; however, 4 more recent observational studies all have confirmed that spinal fusion is associated with some improvement of quality of life, but the duration of these improvements is unknown. Status update since 2013: Newly listed intervention.<br>3 observational studies, using different measures. Spinal fusion conferred improved quality of life at 2 yrs post-operatively. (-1 Risk of Bias; -1 Indirectness; -1 Imprecision). Evidence to Decision Considerations: Limited data from 3 observational studies existed at the time of the Toovey meta-analysis; however, 4 more recent observational studies all have confirmed that spinal fusion is associated with some improvement of quality of life, but the duration of these improvements is unknown. Status update since 2013: Newly	Very low	Weak +	Yellow
		DiFazio 2017[212]	–	–				
		Jain 2018[213]	–	–				
		Miyanji 2018[214]	–	–				
		Miller 2019[215]	–	–				

					listed intervention.			
	333 Reduced pain [BS]	Toovey 2017[210]	CP GMFCS IV-V <18 yrs	Spinal Fusion	2 observational studies. Spinal fusion conferred reduced pain. (-1 Risk of Bias; -1 Indirectness; -1 Imprecision). Status update since 2013: Newly listed intervention.	Very low	Weak +	Yellow
	334 Improved function [A]	Toovey 2017[210]	CP GMFCS IV-V <18 yrs	Spinal Fusion	12 observational studies, using different measures. In the 2 studies with validated measures, there was no improvement in function from spinal fusion (-1 Risk of Bias; -1 Indirectness; -1 Imprecision). Evidence to Decision Considerations: Children who require spinal fusion typically have severe motor impairments and changes in function are difficult to achieve due to the lack of selective motor control. Spinal fusion is not indicated for the goal of improving function and needs to be weigh up carefully given the 38% complication rate. Similar to hip surgery in children in GMFCS V children the goal is not to improve function, but limit deformity, decrease pain and improve quality of life. While some recent data has demonstrated improved deformity and quality of life, pain has yet to be studied rigorously in this cohort. Status update since 2013: Newly listed intervention.	Very low	Weak -	Yellow
	335 Improved gross motor function [BS & A]	Toovey 2017[210]	CP GMFCS IV-V <18 yrs	Spinal Fusion	8 observational studies, using different measures and definitions. No improvement in gross motor function from spinal fusion in 6 studies, whereas 2 studies suggested initial improvement that was not sustained long-term. (-1 Risk of Bias; -1 Indirectness; -1 Imprecision). Evidence to Decision Considerations: Spinal fusion is not indicated for the goal of improving gross motor function and needs to be weigh up carefully given the 38% complication rate. Similar to hip surgery in children in GMFCS V children the goal is not to improve function, but limit deformity, decrease pain and improve quality of life. While some recent data has demonstrated improved deformity and quality of life, pain has yet to be studied rigorously in this cohort. Status update since 2013: Newly listed intervention.	Very low	Weak -	Yellow
**165 Single Event Multi-Level Surgery**	336 Improved gross motor function [BS & A]	Amirmudin 2019[216]	CP spastic unilateral & bilateral GMFCS I-V	Single event multi-level surgery	1 RCT & 27 observational studies. Pooled analyses indicated that gross motor function did not improve short-term [SMD 0.01 95%CI -0.48-0.50], or medium term [SMD 0.51 95%CI -0.56-1.58], or long-term [SMD 0.38, 95%CI -0.25-1.01] from single event multi-level surgery. (-1 Risk of Bias; -1 Imprecision). Evidence to Decision Considerations: High acceptability to patients. Small changes in gross motor function can occur after single event multi-level surgery but occur between 1 and 2 yrs after surgery. These functional changes are small and using standardized assessment instruments is critical to capture these changes between 1 and 2 yrs after surgery. Status update since 2013: Newly listed indication.	Low	Weak +	Yellow
	337 Improved gait speed [BS]	Amirmudin 2019[216]	CP spastic unilateral & bilateral GMFCS I-V	Single event multi-level surgery	29 observational studies. Pooled analyses indicated that gait speed worsened short-term in one study [MD -0.04] and did not improve medium term [SMD -0.08 95%CI -0.22-0.07], or long-term [SMD -0.01, 95%CI -0.01-0.25] from single event multi-level surgery. (-1 Risk of Bias; -1 Imprecision). Evidence to Decision Considerations: The natural history of untreated CP is a gradual decline in walking speed. Single event multi-level surgery has demonstrated small decreases in speed in the short term which is expected and maintenance of speed at medium- and long-term function which is better than the natural history. Status update since 2013: Newly listed indication.	Very low	Weak +	Yellow
	338 Improved gait [BS]	Amirmudin 2019[216]	CP spastic unilateral & bilateral GMFCS I-V	Single event multi-level surgery	35 observational studies. Pooled analyses indicated that gait improved from single event multi-level surgery at 1 yr [SMD -0.78, 95%CI -0.98- -0.57] and long-term [SMD -0.80, 95%CI -0.95 - -0.65]. Evidence to Decision Considerations: The greatest gait improvements were seen greater than 1 yr after single event multi-level surgery, demonstrating the need for at least 2 yrs of follow up after single event multi-level surgery. These findings also highlight the importance of continued rehabilitation for at least 2 yrs after single event multi-level surgery. Status update since 2013: No change in quality or recommendations for use gradings.	Very low	Weak +	Yellow

		Lamberts 2016[217]	–	–	Superseded by Amirmudin 2019			
	339 Improved passive ankle dorsiflexion range of motion [BS]	Amirmudin 2019[216]	CP spastic unilateral & bilateral GMFCS I-V	Single event multi-level surgery	17 observational studies. Pooled analyses indicated single event multi-level surgery conferred improved ankle dorsiflexion range of motion compared to resistance training at 1 yr post-operatively [MD 7 95%3-12]. Single event multi-level surgery conferred improved ankle dorsiflexion medium term [SMD 0.48, 95%CI 0.10-0.86], and long-term [SMD 0.47, 95%CI 0.17-0.76] (-1 Imprecision). Evidence to Decision Considerations: Single event multi-level surgery demonstrates improvement in the plantar flexion knee extension couple, with positive changes in both ankle and knee range of motion after surgery. Status update since 2013: Newly listed indication.	Very low	Weak +	Yellow
		Lamberts 2016[217]	–	–	Superseded by Amirmudin 2019			
	340 Improved passive knee extension range of motion [BS]	Amirmudin 2019[216]	CP spastic unilateral & bilateral GMFCS I-V	Single event multi-level surgery	15 observational studies. Pooled analyses indicated single event multi-level surgery conferred improved knee extension range of motion medium term [SMD 0.60, 95%CI 0.43-0.77] and long-term [SMD 0.43, 95%CI 0.24-0.61]. (-1 Imprecision). Evidence to Decision Considerations: Single event multi-level surgery demonstrates improvement in the plantar flexion knee extension couple, with positive changes in both ankle and knee range of motion after surgery. Status update since 2013: Newly listed indication.	Very low	Weak +	Yellow
		Lamberts 2016[217]	–	–	Superseded by Amirmudin 2019			
	341 Improved muscle strength [BS]	Amirmudin 2019[216]	CP spastic unilateral & bilateral GMFCS I-V	Single event multi-level surgery	14 observational studies. Pooled analyses indicated single event multi-level surgery did not improve muscle strength [SMD -0.22, 95%CI -0.50- 0.06]. (-1 Imprecision). Evidence to Decision Considerations: Single event multi-level surgery does result in expected muscle weakness in the early follow up period, but results at 1 yr demonstrated improved strength compared to resistance training alone. Status update since 2013: Newly listed indication.	Very low	Weak +	Yellow
	342 Reduced spasticity [BS]	Amirmudin 2019[216]	CP spastic unilateral & bilateral GMFCS I-V	Single event multi-level surgery	6 observational studies. Single event multi-level surgery appeared to confer reduced spasticity short-term, but not long-term. Evidence to Decision Considerations: Spasticity reduction is not the intended purpose of orthopaedic surgery and the clinical relevance of these data is debateable. While short term changes in spasticity were identified after single event multi-level surgery these changes are likely the effects of muscle lengthening and not a "true" change in muscle spasticity. The long-term follow up after single event multi-level surgery found that spasticity does not change and these results are expected as this intervention is not designed for spasticity control. Status update since 2013: Newly listed indication.	Very low	Weak -	Yellow
	343 Reduced equinovarus [BS]	Firth 2013[218]	CP diplegia GMFCS II-III 7 yrs	Single event multi-level surgery	1 observational study. Surgical treatment for equinus was successful, when combined with multilevel surgery, orthoses, and rehabilitation. Rate of revision surgery for recurrent equinus was 12.5%. Evidence to Decision Considerations: Treatment of equinus within a single event multi-level surgery, rather than in isolation, appears to lessen the prevalence of crouch gait as a complication. Status update since 2013: Newly listed indication.	Low	Weak +	Yellow
**166 Soft Tissue Surgery for Crouch Gait**	344 Improved knee range of motion [BS]	Galey 2017[219]	CP GMFCS I-IV <18 yrs	Hamstring lengthening or transfer with associated soft tissue procedures	27 observational studies, where crouch was poorly defined, with the exception of 3 studies, affecting interpretation. Only 1 had an adequate control group and no comparative studies identified. Hamstring lengthening, or transfer +/- rectus femoris transfer or psoas lengthening were examined. Hamstring lengthening was the only procedure to confer improved maximum knee flexion in swing (sagittal plane motion). (-1 Risk of Bias; -1 Imprecision). Status update since 2013: Newly listed intervention.	Very low	Weak +	Yellow
**167 Soft Tissue**	345 Reduced hip	Jung	CP diplegia	–	3 observational studies. Pooled analyses indicated hamstring lengthening + rectus	Very low	Weak +	Yellow

**Surgery for Pelvic & Hip Rotation**	internal rotation [BS]	2016[220]	6-19 yrs		femoris transfer lessened pelvic rotation by 3.61 degrees (95%CI -6.13 to -1.09), and lessened hip internal rotation by 6.6 degrees (95%CI 3.34-9.86). (-1 Risk of Bias; -1 Imprecision). Status update since 2013: Newly listed intervention.			
168 **Surgical Correction of Esotropia / Exotropia**	346 Prevention of vision impairment [BS]	Chorna 2017[12]	CP<br>GMFCS I-V<br>6 mo – 14 yrs	–	1 Observational study. Correction of Esotopia via bilateral medial rectus recession and correction of exotropia via bilateral lateral rectus recession, may be beneficial before 2 yrs of age to optimise vision, as correction of alignment is possible. Status update since 2013: Newly listed intervention.	Low	Weak +	Yellow
**REGENERATIVE MEDICINE INTERVENTIONS FOR TREATING CEREBRAL PALSY**								
169 **Stem Cells / Cell Therapies – Neural-like cells [OECs, NSCs, NPCs]**	347 Improved gross motor function [BS & A]	Novak 2016[221]	All CP types and severities<br>0-32 yrs	Variable (1-2 doses 2x10^{6}-2x10^{7} cells)	2 RCTs and 1 nonrandomized trial. Neural like stem cells conferred better improvements in gross motor function than rehabilitation alone (-1 Risk of bias, -1 Inconsistency). Status update since 2013: Newly listed intervention.	Low	Weak +	Yellow
		Kułak-Bejda 2016[222]	–	–	Superseded by Novak 2016.			
	348 Improved cognition [BS]	Novak 2016[221]	All CP types and severities<br>0-3.5 yrs	1 dose of 8–10x10^{6} cells	1 RCT showed improved cognition at 12 mo post intervention. Neural like stem cells may have conferred better cognitive gains than rehabilitation alone but the results must be interpreted cautiously as the measures are unvalidated (-1 Risk of bias; -1 Indirectness; -1 Imprecision). Status update since 2013: Newly listed intervention.	Very Low	Weak +	Yellow
170 **Stem Cells / Cell Therapies – Umbilical Cord Blood**	349 Improved gross motor function [BS & A]	Novak 2016[221]	All CP types and severities<br>10 mo – 20 yrs	1 dose of variable concentration of 1-5x10^{7} cells/kg	3 RCTs. Umbilical Cord Blood conferred small but better improvements in gross motor function than rehabilitation alone. (-1 Inconsistency). Evidence to Decision Considerations: There is preliminary evidence of a dose response, i.e higher doses result in bigger gains in gross motor function, and an infusion must reach a threshold dose to confer any gains. Benefits outweigh harms, as rates of adverse events are negligible. Status update since 2013: Newly listed intervention.	Moderate	Strong +	Green
		Kułak-Bejda 2016[222]	–	–	Superseded by Novak 2016.			
	350 Improved cognition/social cognition [BS]	Novak 2016[221]	CP. Type & severity not defined<br>10 mo – 10 yrs	1 dose of 3x10^{7} cells/kg	1 RCT. Umbilical Cord Blood may have conferred better cognitive gains than rehabilitation alone, but the results must be interpreted cautiously (-1 Risk of Bias; -1 Confounders EPO and immunosuppression). Status update since 2013: Newly listed intervention.	Low	Weak +	Yellow
171 **Stem Cells / Cell Therapies – Mesenchymal stem/stromal cells**	351 Improved gross motor function [BS & A]	Huang 2018[223]<br>Liu 2017[224]	CP type & severity not defined<br>6 mo – 12 yrs	Variable (4 doses at 1x10^{6} or 8 doses at 5x10^{7} cells/kg)	2 RCTs. Mesenchymal stromal cells conferred better improvements in gross motor function than control bone marrow mononuclear cells or placebo with rehabilitation (-1 Risk of bias). Evidence to Decision Considerations: There was variability in both the cell source and delivery route. Status update since 2013: Newly listed intervention.	Moderate	Weak +	Yellow
	352 Improved fine motor function [BS & A]	Liu 2017[224]	CP spastic<br>GMFCS II–V<br>6 mo – 11 yrs	4 doses at 1x10^{6} cells/kg	1 RCT. Bone marrow mesenchymal stem cells conferred better improvements in fine motor function than control bone marrow mononuclear cells (-2 Risk of bias). Status update since 2013: Newly listed intervention.	Low	Weak +	Yellow
	353 Improved function [A]	Huang 2018[223]	CP type & severity not defined<br>3-12 yrs	8 doses at 5x10^{7} cells/kg	1 RCT. Mesenchymal stromal cells conferred better improvements in function than placebo with rehabilitation, but results must be interpreted cautiously due to the psychometrics of the Comprehensive Function Assessment measure. (-2 Risk of bias; -1 Indirectness). Status update since 2013: Newly listed intervention.	Very Low	Weak +	Yellow
172 **Stem Cells / Cell Therapies**	354 Improved gross motor	Rah 2017[225]<br>Liu 2017[222]	CP spastic or not specified	Variable (4 doses at	2 RCTs. Mobilized peripheral blood/bone marrow mononuclear cells conferred improved gross motor function compared to placebo or control bone marrow mononuclear cells. (-1	Low	Weak +	Yellow

**– Mobilized peripheral blood/bone marrow mononuclear cells**	function [BS & A]		GMFCS II-V<br>2-12.5 yrs	1x10^6 or 1 at 5.2x10^8 (+/- 2.54) cells/kg)	Risk of Bias; -1 Confounders). Evidence to Decision Considerations: Study that used G-CSF stimulation followed by mobilized peripheral blood mononuclear cell reinfusion saw largest improvements after G-CSF stimulation alone, which may have been the contributing factor. Unclear if benefits are from G-CSF or from cells. Variability in cell source and administration (route/dose/repeat dosing) was an additional confounder. Status update since 2013: Newly listed intervention.			
	355 Improved fine motor function [BS & A]	Liu 2017[222]	CP spastic<br>GMFCS II–V<br>6 mo – 12.5 yrs	4 doses at 1x10^6 cells/kg	1 RCT. No difference between groups for fine motor function for mobilized peripheral blood/bone marrow compared to control bone marrow mononuclear cells (-2 Risk of Bias). Status update since 2013: Newly listed intervention.	Low	Weak +	Yellow
		Park 2017[226]	–	–	Duplicate participant data, therefore, not reported twice.			
**COMPLIMENTARY AND ALTERNATIVE MEDICINE INTERVENTIONS FOR MANAGING CEREBRAL PALSY**								
**173 Acupuncture**	356 Improved gross motor function [A]	Li 2018[227]	CP<br>0.5-14yrs	1-5x week	21 small RCTs some published and unpublished in Chinese. Authors conclude acupuncture confers improved gross motor function better than rehabilitation (-1 Risk of Bias; -1 Publication Bias). Status update since 2013: No change in quality or recommendations for use gradings.	Low	Weak +	Yellow
		Mandziuk 2012[228]	CP	1-7x wk	6 small RCTs and 1 quasi-randomised trial. Authors conclude acupuncture confers improved movement and function better than control music or play or sham, however multiple studies are comparing differing doses of acupuncture not acupuncture to control (-2 Risk of Bias; -1 Imprecision; -1 Other – improvements are not clinically meaningful change). Status update since 2013: No change in quality or recommendations for use gradings.	Very low	Weak +	Yellow
	357 Reduced spasticity [BS]	Kwon 2019[229]	CP spastic<br>1-16 yrs	1x mo	7 small RCTS combined in a meta-analysis showed acupuncture (acupotomy approach i.e. acupuncture + microsurgery) probably conferred reduction of spasticity better than control NDT (-1 Risk of Bias; -1 Imprecision). Status update since 2013: Newly listed indication.	Low	Weak +	Yellow
	358 Reduced symptoms [BS]	Li 2014[230]	All CP<br>1-18 yrs	1-3x mo	7 small RCTS combined in a meta-analysis showed scalp acupuncture probably conferred symptom reduction better than control medication or rehabilitation. However, this systematic review has a very serious risk of bias, in that non-responders were excluded from the meta-analysis. (-2 Risk of Bias; -1 Imprecision). Status update since 2013: Newly listed indication.	Very low	Weak +	Yellow
		Li 2018[227]	CP<br>0.5-14 yrs	1-5x wk	21 small RCTs some published and unpublished in Chinese. Authors conclude acupuncture confers symptom reduction better than rehabilitation (-1 Risk of Bias; -1 Publication Bias). Status update since 2013: Newly listed indication.	Low	Weak +	Yellow
**174 Animal Assisted Therapy**	359 Improved socialisation [P]	–	–	–	Status update since 2013: No new primary source data, no change in gradings.	Very low	Weak +	Yellow
	360 Improved independence via service dogs [P]	–	–	–	Status update since 2013: No new primary source data, no change in gradings.	Very low	Weak +	Yellow
	361 Improved gross motor function [BS & A]	Charry-Sánchez 2018[102]	CP mixed types<br>3-16 yrs<br>CP mixed types<br>3-16 yrs	Daily<br>Daily<br>4 hrs a day, 5x wk for 3	7 Controlled Trials, 3 observational studies in CP. Pooled analysis showed animal assisted therapy may confer improved gross motor skills on GMFM-66 [MD 1.61, 95%CI -2.00-5.23], crosses line of no effect. (-1 Risk of Bias; -1 Imprecision). Evidence to Decision Considerations: Costs. High acceptability. Status update since 2013: Newly	Low	Weak +	Yellow

			CP mixed types 3-6 yrs	wks	listed indication.			
	362 Reduced pain [BS]				7 Controlled Trials, 3 observational studies in CP. Pooled analysis showed animal assisted therapy may confer reduced pain [MD -0.81, 95%CI -1.32-0.30], crosses line of no effect. (-1 Risk of Bias; -1 Imprecision; -1 Indirectness). Evidence to Decision Considerations: Costs. High acceptability. Status update since 2013: Newly listed indication.	Very low	Weak +	Yellow
**175 Conductive Education [CE]**	363 Improved gross motor function ('orthofunction') [BS & A]	Myrhaug 2018[231]<br>Myrhaug 2019[232]	CP mixed types 3-6yrs	4hrs a day, 5 x week for 3 wks	1 RCT (Myrhaug 2018), with an 8-12 mo long-term follow up (Myrhaug 2019). RCT showed no between group differences in motor function for conductive education using structured training and child-parent-trainer set goals compared to conventional care. (-1 Risk of Bias; -1 Imprecision). Evidence to Decision Considerations: Multiple effective substitute interventions exist. Status update since 2013: No change in quality or recommendation for use grading since 2013 review.	Low	Weak -	Yellow
	364 Improved function [A]	Myrhaug 2018[231]<br>Myrhaug 2019[232]	CP mixed types 3-6 yrs	4 hrs a day, 5x wk for 3 wks	1 RCT, with an 8-12 mo long-term follow up (Myrhaug 2019). RCT showed no between group differences in function for conductive education using structured training and child-parent-trainer set goals compared to conventional care. (-1 Risk of Bias; -1 Imprecision). Evidence to Decision Considerations: Multiple effective substitute interventions exist. Status update since 2013: No change in quality or recommendation for use grading since 2013 review.	Low	Weak -	Yellow
	365 Child quality of life & social function [P]	Myrhaug 2018[231]	CP mixed types 3-6 yrs	4 hrs a day, 5x wk for 3 wks	1 RCT. RCT showed no between group differences in social function short term, but a gain at 4 mo with no long term follow up. (-1 Risk of Bias; -1 Imprecision). Status update since 2013: Newly listed indication.	Low	Weak +	Yellow
	366 Parent quality of life [EF]	Myrhaug 2018[231]	CP mixed types 3-6 yrs	4 hrs a day, 5x wk for 3 wks	1 RCT. RCT showed no between group differences in parent quality of life. (-1 Risk of Bias; -1 Imprecision). Status update since 2013: Newly listed indication.	Low	Weak +	Yellow
	367 Parent perception of family centered [EF]	Myrhaug 2018[231]	CP mixed types 3-6 yrs	4 hrs a day, 5x wk for 3 wks	1 RCT. Parents preferred having conductive education to no services, and perceived that having conductive education was more family centred than no services (-1 Risk of Bias; -1 Imprecision; -1 Indirectness). Status update since 2013: Newly listed indication.	Very Low	Weak +	Yellow
**176 Herbal Medicine**	368 Improved function [A]	Lee 2018[233]	CP	Not reported	5 RCTs. Oriental herbal medicine + rehabilitation conferred better function than control rehabilitation. Unable to obtain primary data in Chinese, Lee et al. coded the RCTs as low quality. (-1 Risk of Bias; -1 Indirectness; -1 Imprecision). Status update since 2013: Newly listed intervention.	Low	Weak +	Yellow
**177 Hyperbaric Oxygen [HBO2]**	369 Improved gross motor function [BS & A]	Novak 2013[234]	CP	Varied	3 RCTs. 1 meta-analysis of 2 RCTs. Hyperbaric air improved gross motor function better than hyperbaric oxygen, with 1 RCT crossing the line of no effect [MD -8.93, 95% CI -11.40 to -6.46]. (-1 Imprecision). Evidence to Decision Considerations: Costs, risks of harm exist including hearing loss and pain. Status update since 2013: Newly listed indication.	Moderate	Strong -	Red
	370 Improved self-care [A]	Novak 2013[234]	CP	Varied	3 RCTs. 1 meta-analysis of 2 RCTs. Hyperbaric air improved self-care better than hyperbaric oxygen, with 1 RCT crossing the line of no effect [MD-2.42, 95%CI -4.29 to -0.55]. (-1 Imprecision). Evidence to Decision Considerations: Costs, risks of harm exist including hearing loss and pain. Status update since 2013: Quality downgraded from strong to moderate. Recommendations for use unchanged.	Moderate	Strong -	Red
	371 Improved sleep [BS]	Long 2017[235]	CP 2-6 yrs	10 & 20 mins HBO_2 compression 15-20x	1 Controlled Trial, groups allocated based on age. HBO_2 conferred improved sleep onset, easy awakening and prolonged sleeping time in younger children after 10 sessions. Authors proposed the treatment was safe and feasible. However, confounders exist including excluding participants with adverse events form the analysis and excluding	Low	Strong -	Red

					participants with missing data from analysis, meaning the results must be interpreted cautiously. Furthermore, gains were not observed in older participants (-2 Risk of Bias). Evidence to Decision Considerations: Harms occurred including a seizure during decompression, ear discomfort, and crying. Status update since 2013: Newly listed indication.			
**178 Massage**	372 Reduced pain [BS]	–	–	–	Status update since 2013: No new primary source data, no change in quality or recommendations for use gradings.	Low	Weak +	Yellow
	373 Reduced spasticity [BS]	Rasool 2017[236]	CP diplegia	30 mins a day, 5x wk for 6 wks	1 RCT. No between group significant differences for massage + physiotherapy v physiotherapy alone for reducing spasticity after 6 wks on MAS. (-1 Risk of Bias: no baseline equivalence). Evidence to Decision Considerations: Effective substitute spasticity management options exist therefore as massage cannot be recommended as a frontline treatment for spasticity management. Status update since 2013: Quality upgraded from low to moderate. Recommendations for use downgraded from weak positive to weak negative.	Moderate	Weak -	Yellow
	374 Improved function [A]	–	–	–	Status update since 2013: No new primary source data, no change in quality or recommendations for use gradings.	Low	Weak +	Yellow
	375 Improved sleep [BS]	Galland 2012[29]<br>Rigney 2018[150]	CP <16 yrs	Not reported	2 observational studies. Massage conferred improved parent perceptions of their child's sleep. (-1 Risk of Bias; -1 Indirectness; -1 Imprecision). Evidence to Decision Considerations: Benefits outweigh harms. High acceptability. Status update since 2013: Newly listed indication.	Very low	Weak +	Yellow
	376 Improved defecation frequency [BS]	Orhan 2018[237]	CP & chronic constipation <18 yrs	15-20 mins 3x wk for 4 wks	1 RCT. Connective tissue manipulation conferred improved defecation frequency compared to control taping at the same dose. (-1 Risk of Bias). Status update since 2013: Newly listed indication.	Moderate	Strong +	Green
	377 Improved gross motor function [BS & A]	Silva 2012[238]	CP or Downs Syndrome <4 yrs	15 mins daily for 5 mo	1 small RCT. Qigong Massage conferred improved gross motor skills compared to wait list control. However this study has a number of serious confounders and the results must be interpreted very cautiously: n=28 participants, 50% CP 50% Downs Syndrome, 30% dropouts in control group. (-2 Risk of Bias; -1 Indirectness). Evidence to Decision Considerations: Multiple effective substitute interventions exist for improving gross motor. Status update since 2013: Newly listed indication.	Very low	Weak -	Yellow
	378 Improved sensory function [BS]	Silva 2012[238]	CP or Downs Syndrome <4 yrs	15 mins daily for 5 mo	1 small RCT. No between group differences for Qigong Massage and wait list control for sensory function. However this study has a number of serious confounders and the results must be interpreted very cautiously: n=28 participants, 50% CP 50% Downs Syndrome, 30% dropouts in control group. (-2 Risk of Bias; -1 Indirectness). Status update since 2013: Newly listed indication.	Very low	Weak -	Yellow
**179 Osteopathy [Incl. Cranial Sacral Osteopathy]**	379 Improved gross motor function [BS & A]	Cerritelli 2016[239]<br>Posadzki 2013[240]<br>Prevost 2019[241]	CP 0-12 yrs	6-12 sessions 0.3-1 hr x fortnight	3 systematic reviews have studied the same 3 RCTs for CP. All 3 RCTs showed no between group differences (-1 Risk of Bias). Evidence to Decision Considerations: Effective substitute interventions exist for improving motor function. Status update since 2013: No change in quality or recommendation for use grading since 2013 review.	Moderate	Strong -	Red
	380 Reduced constipation [BS]	Prevost 2019[241]	CP 2-16 yrs	Not reported	1 small observational study showing osteopathy conferred better reduction in constipation than control (-1 Risk of Bias; -1 Indirectness; -1 Imprecision). Status update since 2013: Newly listed indication.	Very low	Weak +	Yellow
	381 Improved sleep [BS]	Galland 2012[29]	CP	Not reported	2 RCTs. No between group differences for sleep time or sleep onset time for osteopathy compared to control. (-1 Risk of Bias; -1 Indirectness; -1 Imprecision). Status update	Very low	Weak +	Yellow

					since 2013: Newly listed indication.			
**180 Reflexology**	382 Reduced spasticity [BS]	Ozkan 2017[242]	CP spastic 2-18 yrs	45 mins sessions 1x wk	1 RCT. Reflexology conferred decreased spasticity on Tardieu Scale, compared to placebo or control. However there was selective reporting: 25% of sample were excluded with no explanation and unclear reporting of spasticity scale. Unclear what concomitant medications for spasticity management were in use. The results should be interpreted cautiously (-2 Risk of Bias; -1 Imprecision). Evidence to Decision Considerations: Reflexology was described by the authors as an adjunctive intervention. Effective spasticity management substitutes exist. Status update since 2013: Newly listed intervention.	Very low	Weak -	Yellow
	383 Improved gross motor function [BS & A]	Elbasan 2018[243]	CP 3-15 yrs	Reflexology 20 mins for 8 wks	1 RCT. No between group differences for Reflexology + NDT v NDT alone for gross motor skills. Some children received concomitant BoNT, which was a confounder. (-2 Risk of Bias: not a pure control group). Evidence to Decision Considerations: Effective gross motor training substitutes exist. Status update since 2013: Newly listed intervention.	Low	Weak -	Yellow
	384 Reduced constipation [BS]	Elbasan 2018[243]	CP 3-15 yrs	Reflexology 20 mins for 8 wks	1 RCT. Reflexology + NDT conferred reduced constipation compared to NDT alone. (-2 Risk of Bias: not a pure control group). Status update since 2013: Newly listed intervention.	Low	Weak +	Yellow
**181 Vojta**	385 Improved gross motor function [BS & A]	Sun-Young 2018[244]	CP GMFCS I-III 3-6 yrs	30 mins 3x wk, for 6 wks	1 RCT. No between group differences for Vojta v trunk strengthening + gait training for gross motor function. (-2 Risk of Bias). Evidence to Decision Considerations: Effective gross motor training substitutes exist. Status update since 2013: No change in quality or recommendation for use grading since 2013 review.	Low	Weak -	Yellow
		Franki 2012[245]	CP <1 yrs		1 Controlled Trial, non-randomised. (-2 Risk of Bias). Authors conclude Vojta conferred improved gross motor function. However this study has a number of serious confounders and the results must be interpreted very cautiously: the group allocation were assigned by how much therapy the child did retrospectively ie the high intensity was Vojta, the control was low-dose Vojta or no therapy; the children were ex-preterm and had risks for CP not a confirmed diagnosis or known severity at baseline. Franki 2012 concluded the effectiveness of Vojta remains unknown. Status update since 2013: No change in quality or recommendation for use grading since 2013 review.			
		Myrhaug 2014[246]	–	–	Superseded by Franki 2012.			
	386 Improved diaphragm size during inspiration [BS]	Sun-Young 2018[244]	CP GMFCS I-III 3-6 yrs	30 mins 3x wk, for 6 wks	1 RCT. Vojta conferred improved diaphragm size during inspiration compared to control muscle strengthening and gait training. (-2 Risk of Bias). Evidence to Decision Considerations: Effective gross motor training substitutes exist. Status update since 2013: Newly listed indication.	Low	Weak +	Yellow
**182 Yoga**	387 Improved muscle strength, flexibility, and balance [BS]	Veneri 2018 [247]	CP Diplegia GMFCS II 9 yrs	1x 1 hr/wk for 6 wks plus PT 2x1 hr/wk	1 observational study. Yoga plus physical therapy may confer small improvements in muscle strength, flexibility, and balance. (-1 Risk of bias). Status update since 2013: Newly listed intervention.	Very Low	Weak +	Yellow
	388 Improved attention [BS]	Mak 2018[248]	CP unilateral & bilateral GMFCS I-III 6-16 yrs	8 wks, 6x 1.5 hr + 20 mins daily home program	1 RCT. Yoga conferred better attention, less impulsivity immediately after intervention compared to waitlist control, on the Conners' Continuous Performance Test 2nd Ed. (-1 Risk of Bias). Status update since 2013: Newly listed intervention.	Moderate	Weak +	Yellow
	389 Improved mindfulness	Mak 2018[248]	CP unilateral & bilateral	8 wks, 6x 1.5 hr + 20	1 RCT. No between group differences for yoga or waitlist control for mindfulness of the Child and Adolescent Mindfulness Measure. (-1 Risk of bias). Evidence to Decision	Moderate	Weak -	Yellow

	[BS]		GMFCS I-III<br>6-16 yrs	mins daily home program	Considerations: No evidence for this indication, but benefits outweigh harms. Status update since 2013: Newly listed intervention.			
	390 Improved executive functioning [BS]	Mak 2018[248]	CP unilateral & bilateral<br>GMFCS I-III<br>6-16 yrs	8 wks, 6x 1.5 hr + 20 mins daily home program	1 RCT. No between group differences for yoga or waitlist control for executive function. (-1 Risk of bias). Evidence to Decision Considerations: Effective substitute interventions exist for targeting executive function. Status update since 2013: Newly listed intervention.	Moderate	Weak -	Yellow
	391 Improved behaviour [BS]	Mak 2018[248]	CP unilateral & bilateral<br>GMFCS I-III<br>6-16 yrs	8 wks, 6x 1.5 hr + 20 mins daily home program	1 RCT. No between group differences for yoga or waitlist control for behaviour. (-1 Risk of bias). Evidence to Decision Considerations: Effective substitute interventions exist for improving behaviour. Status update since 2013: Newly listed intervention.	Moderate	Weak -	Yellow
	392 Improved gross motor [BS & A]	Mak 2018[248]	CP unilateral & bilateral<br>GMFCS I-III<br>6-16 yrs	8 wks, 6x 1.5 hr + 20 mins daily home program	1 RCT. No between group differences for yoga or waitlist control for gross motor function. Authors conclude that no change may be attributed to the participant's focus on mindfulness training and not physical training at home. (-1 Risk of Bias). Evidence to Decision Considerations: Effective substitute interventions exist for improving gross motor function. Status update since 2013: Newly listed intervention.	Moderate	Weak -	Yellow
	393 Reduced pain [BS]	Mak 2018[248]	CP unilateral & bilateral<br>GMFCS I-III<br>6-16 yrs	8 wks, 6x 1.5 hr + 20 mins daily home program	1 RCT. No between group differences for yoga or waitlist control for pain, however the participants did not have pain at baseline. Therefore this is not a clinically relevant outcome to measure in this study. (-1 Risk of Bias; -1 Indirectness). Evidence to Decision Considerations: Effective substitute interventions exist for reducing pain. Status update since 2013: Newly listed intervention.	Moderate	Weak -	Yellow

Abbreviations:
A – activities; AAC – Augmentative and Alternative Communication; ACT – Acceptance & Commitment Therapy; AdSS – adaptive seating systems; AFOs – ankle foot orthoses; ASD – Autism Spectrum Disorder; B body structures and function; BoNT - Botulinum Toxin A; CBT – Cognitive Behaviour Therapy; CE – Conductive Education; CFCS – Communication Function Classification System; CI – confidence interval; CIM Constraint Induced Movement Therapy; CO-OP – Cognitive Orientation to Occupational Performance; COPCA – Coping with and Caring for Infants with Special Needs; COPM – Canadian Occupational Performa Measure; CP – cerebral palsy; CTG – cardiotocography; DBS – deep brain stimulation; EF – environmental factors; GA – gestational age; GAME – Goals Activity Motor Enrichment; GAS – Goal Attainment Scaling CSF – Granulocyte-colony stimulating factor; GMFCS – Gross Motor Function Classification System; GMFM – Gross Motor Function Measure; HABIT-ILE – hand-arm bimanual intensive therapy including lo extremities; HBO2 – hyperbaric oxygen; hr/s – hour/s; ID – intellectual disability; ITB – Baclofen, intrathecal; MAS – Modified Ashworth scale; MD – mean difference; mins – minutes; mo – months; ND Neurodevelopmental Therapy; NPC – neural progenitor cell; NSC – neural stem cell; OEC – olfactory ensheathing cell; OR – odds ratio; OT – occupational therapy; P – participation; PBWSTT – partial body we support treadmill training; PF – personal factors; PT – physical therapy; RCT – randomized controlled trial; RF-DRG – radio-frequency lesion of the dorsal root ganglion; rhEPO – recombinant human Erythropoietin; – Relative Risk; SDR – selective dorsal rhizotomy; sec – seconds; SMD - Standardised Mean Difference; SPP – sleep positioning systems; tDCS – transcranial direct current stimulation; v – versus; VR – virtual rea wk/s – week/s; yr/s – year/s
